# Predicting attitudinal and behavioral responses to COVID-19 pandemic using machine learning

**DOI:** 10.1093/pnasnexus/pgac093

**Published:** 2022-07-05

**Authors:** Tomislav Pavlović, Flavio Azevedo, Koustav De, Julián C Riaño-Moreno, Marina Maglić, Theofilos Gkinopoulos, Patricio Andreas Donnelly-Kehoe, César Payán-Gómez, Guanxiong Huang, Jaroslaw Kantorowicz, Michèle D Birtel, Philipp Schönegger, Valerio Capraro, Hernando Santamaría-García, Meltem Yucel, Agustin Ibanez, Steve Rathje, Erik Wetter, Dragan Stanojević, Jan-Willem van Prooijen, Eugenia Hesse, Christian T Elbaek, Renata Franc, Zoran Pavlović, Panagiotis Mitkidis, Aleksandra Cichocka, Michele Gelfand, Mark Alfano, Robert M Ross, Hallgeir Sjåstad, John B Nezlek, Aleksandra Cislak, Patricia Lockwood, Koen Abts, Elena Agadullina, David M Amodio, Matthew A J Apps, John Jamir Benzon Aruta, Sahba Besharati, Alexander Bor, Becky Choma, William Cunningham, Waqas Ejaz, Harry Farmer, Andrej Findor, Biljana Gjoneska, Estrella Gualda, Toan L D Huynh, Mostak Ahamed Imran, Jacob Israelashvili, Elena Kantorowicz-Reznichenko, André Krouwel, Yordan Kutiyski, Michael Laakasuo, Claus Lamm, Jonathan Levy, Caroline Leygue, Ming-Jen Lin, Mohammad Sabbir Mansoor, Antoine Marie, Lewend Mayiwar, Honorata Mazepus, Cillian McHugh, Andreas Olsson, Tobias Otterbring, Dominic Packer, Jussi Palomäki, Anat Perry, Michael Bang Petersen, Arathy Puthillam, Tobias Rothmund, Petra C Schmid, David Stadelmann, Augustin Stoica, Drozdstoy Stoyanov, Kristina Stoyanova, Shruti Tewari, Bojan Todosijević, Benno Torgler, Manos Tsakiris, Hans H Tung, Radu Gabriel Umbreș, Edmunds Vanags, Madalina Vlasceanu, Andrew J Vonasch, Yucheng Zhang, Mohcine Abad, Eli Adler, Hamza Alaoui Mdarhri, Benedict Antazo, F Ceren Ay, Mouhamadou El Hady Ba, Sergio Barbosa, Brock Bastian, Anton Berg, Michał Białek, Ennio Bilancini, Natalia Bogatyreva, Leonardo Boncinelli, Jonathan E Booth, Sylvie Borau, Ondrej Buchel, Chrissie Ferreira de Carvalho, Tatiana Celadin, Chiara Cerami, Hom Nath Chalise, Xiaojun Cheng, Luca Cian, Kate Cockcroft, Jane Conway, Mateo A Córdoba-Delgado, Chiara Crespi, Marie Crouzevialle, Jo Cutler, Marzena Cypryańska, Justyna Dabrowska, Victoria H Davis, John Paul Minda, Pamala N Dayley, Sylvain Delouvée, Ognjan Denkovski, Guillaume Dezecache, Nathan A Dhaliwal, Alelie Diato, Roberto Di Paolo, Uwe Dulleck, Jānis Ekmanis, Tom W Etienne, Hapsa Hossain Farhana, Fahima Farkhari, Kristijan Fidanovski, Terry Flew, Shona Fraser, Raymond Boadi Frempong, Jonathan Fugelsang, Jessica Gale, E Begoña García-Navarro, Prasad Garladinne, Kurt Gray, Siobhán M Griffin, Bjarki Gronfeldt, June Gruber, Eran Halperin, Volo Herzon, Matej Hruška, Matthias F C Hudecek, Ozan Isler, Simon Jangard, Frederik Jørgensen, Oleksandra Keudel, Lina Koppel, Mika Koverola, Anton Kunnari, Josh Leota, Eva Lermer, Chunyun Li, Chiara Longoni, Darragh McCashin, Igor Mikloušić, Juliana Molina-Paredes, César Monroy-Fonseca, Elena Morales-Marente, David Moreau, Rafał Muda, Annalisa Myer, Kyle Nash, Jonas P Nitschke, Matthew S Nurse, Victoria Oldemburgo de Mello, Maria Soledad Palacios-Galvez, Yafeng Pan, Zsófia Papp, Philip Pärnamets, Mariola Paruzel-Czachura, Silva Perander, Michael Pitman, Ali Raza, Gabriel Gaudencio Rêgo, Claire Robertson, Iván Rodríguez-Pascual, Teemu Saikkonen, Octavio Salvador-Ginez, Waldir M Sampaio, Gaia Chiara Santi, David Schultner, Enid Schutte, Andy Scott, Ahmed Skali, Anna Stefaniak, Anni Sternisko, Brent Strickland, Jeffrey P Thomas, Gustav Tinghög, Iris J Traast, Raffaele Tucciarelli, Michael Tyrala, Nick D Ungson, Mete Sefa Uysal, Dirk Van Rooy, Daniel Västfjäll, Joana B Vieira, Christian von Sikorski, Alexander C Walker, Jennifer Watermeyer, Robin Willardt, Michael J A Wohl, Adrian Dominik Wójcik, Kaidi Wu, Yuki Yamada, Onurcan Yilmaz, Kumar Yogeeswaran, Carolin-Theresa Ziemer, Rolf A Zwaan, Paulo Sergio Boggio, Ashley Whillans, Paul A M Van Lange, Rajib Prasad, Michal Onderco, Cathal O'Madagain, Tarik Nesh-Nash, Oscar Moreda Laguna, Emily Kubin, Mert Gümren, Ali Fenwick, Arhan S Ertan, Michael J Bernstein, Hanane Amara, Jay Joseph Van Bavel

**Affiliations:** Institute of Social Sciences Ivo Pilar, Zagreb, Croatia; Department of Psychology, Cambridge University, Cambridge, UK; Department of Finance and Quantitative Methods, Gatton College of Business and Economics, University of Kentucky, Lexington, KY, USA; Faculty of Medicine, Cooperative University of Colombia, Villavicencio, Meta, Colombia and Department of bioethics, El Bosque University, Bogotá D.C. Colombia; Institute of Social Sciences Ivo Pilar, Zagreb, Croatia; Department of Philosophy and Social Studies, University of Crete, Rethymnon, Crete, Greece; Department of Research and Development, Kozaca SA, Rosario, Santa Fe, Argentina; Direccion Academica Sede la Paz,Universidad Nacional de Colombia - Sede de La Paz, Cesar, Colombia; Department of Media and Communication, City University of Hong Kong, Kowloon Tong, Hong Kong; Department of Economics, Institute of Security and Global Affairs, Leiden University, The Hague, Netherlands; School of Human Sciences, University of Greenwich, London, UK; Department of Philosophy, School of Economics and Finance, University of St Andrews, St Andrews, UK; Department of Economics, Middlesex University London, London, UK; Department of Psychiatry, Pontifical Xavierian University, Bogotá, Colombia; Department of Psychology and Neuroscience, Duke University, Durham, NC, USA; Latin American Brain Health Institute (BrainLat), Universidad Adolfo Ibañez, Santiago, Peñalolén, Chile; Cognitive Neuroscience Center (CNC), University of San Andrés and CONICET, Buenos Aires, Argentina; Global Brain Health Institute, University of California - San Francisco, San Francisco, CA, USA; Global Brain Health Institute, Trinity College, Dublin, Ireland; Department of Psychology, University of Cambridge, Cambridge, UK; Department of Business Administration, Stockholm School of Economics, Stockholm, Sweden; Department of Sociology, Faculty of Philosophy, University of Belgrade, Belgrade, Serbia; Department of Experimental and Applied Psychology, Vrije Universiteit Amsterdam, Amsterdam, Netherlands; Departamento de Matemática y Ciencias, Universidad de San Andres, Victoria, Buenos Aires, Argentina; Department of Management, Aarhus University, Aarhus, Denmark; Institute of Social Sciences Ivo Pilar, Zagreb, Croatia; Department of Psychology, Faculty of Philosophy, University of Belgrade, Belgrade, Serbia; Department of Management, Aarhus University, Aarhus, Denmark; School of Psychology, University of Kent, Canterbury, UK; Stanford Graduate School of Business, Stanford University, Stanford, California, USA; Department of Philosophy, Macquarie University, Macquarie Park, New South Wales, Australia; Department of Philosophy, Macquarie University, Macquarie Park, New South Wales, Australia; Department of Strategy and Management, Norwegian School of Economics, Bergen, Norway; Institute of Psychology, Center for Climate Action and Social Transformations, SWPS, University of Social Sciences and Humanities, Warsaw, Poland; Institute of Psychology, SWPS University of Social Sciences and Humanities, Warsaw, Poland; Centre for Human Brain Health,School of Psychology, University of Birmingham, Birmingham, UK; Department of Experimental Psychology, University of Oxford, Oxford, UK; Centre for Sociological Research, Katholieke Universiteit Leuven, Leuven, Belgium; Faculty of Psychology, Higher School of Economics University, Moscow, Russia; Department of Psychology, University of Amsterdam, Amsterdam, Netherlands; Centre for Human Brain Health,School of Psychology, University of Birmingham, Birmingham, UK; Department of Psychology, Sunway University, Petaling Jaya, Selangor, Malaysia; Department of Psychology, University of the Witwatersrand, Johannesburg, Republic of South Africa; Department of Political Science, Aarhus University, Aarhus, Denmark; Department of Psychology, Toronto Metropolitan University, Toronto, Ontario, Canada; Department of Psychology, University of Toronto, Toronto, Ontario, Canada; Department of Mass Communication, National University of Science and Technology (NUST), Islamabad, Islamabad Capital Territory, Pakistan; Department of Psychology, University of Greenwich, London, UK; Institute of European Studies and International Relations, Faculty of Social and Economic Sciences, Comenius University, Bratislava, Slovakia; Macedonian Academy of Sciences and Arts, Skopje, Republic of North Macedonia; Department of Sociology, Social Work and Public Health, University of Huelva, Huelva, Spain; Department of Decision Analytics and Risk, University of Southampton, Southampton, UK; Department of Educational and Counselling Psychology, BRAC Institute of Educational and Development, BRAC University, Dhaka, Bangladesh; Department of Psychology, Hebrew University of Jerusalem, Jerusalem, Israel; Rotterdam Institute of Law and Economics (RILE), Erasmus University Rotterdam, Rotterdam, Netherlands; Department of Communication Science, Vrije Universiteit Amsterdam, Amsterdam, Netherlands; Kieskompas (Election Compass), Amsterdam, Netherlands; Department of Digital Humanities, University of Helsinki, Helsinki, Finland; Department of Cognition, Emotion, and Methods in Psychology, University of Vienna, Wien, Austria; Department of Neuroscience and Biomedical Engineering, Aalto University, Espoo, Finland; Baruch Ivcher School of Psychology, Reichman University, Herzliya, Israel; School of Psychology, Universidad Nacional Autónoma de Mexico, Mexico City, Mexico; Department of Economics, National Taiwan University, Taipei, Taiwan; HEMS School, Kathmandu, Nepal; Department of Political Science, Aarhus University, Aarhus, Denmark; Department of Leadership and Organizational Behaviour, BI Norwegian Business School, Oslo, Norway; Institute of Security and Global Affairs, Leiden University, The Hague, Netherlands; Department of Psychology, University of Limerick, Limerick, Ireland; Department of Clinical Neuroscience, Karolinska Institutet, Stockholm, Sweden; Department of Management, University of Agder, Kristiansand, Norway; Department of Psychology, Lehigh University, Bethlehem, PA, USA; Department of Digital Humanities, University of Helsinki, Helsinki, Finland; Department of Psychology, Hebrew University of Jerusalem, Jerusalem, Israel; Department of Political Science, Aarhus University, Aarhus, Denmark; Department of Psychology, Monk Prayogshala, Powai, Mumbai, Maharashtra, India; Department of Social and Behavioral Science, Friedrich Schiller University Jena, Jena, Germany; Department of Management, Technology, and Economics, Swiss Federal Institute of Technology in Zürich, Zürich, Switzerland; Chair of Development Economics,University of Bayreuth, Bayreuth, Germany; Department of Sociology, National School for Political and Administrative Studies (SNSPA), Bucharest, Romania; Department of Psychiatry and Medical Psychology, Medical University, Plovdiv, Bulgaria; Division of Translational Neuroscience, Medical University, Plovdiv, Bulgaria; Humanities and Social Sciences, Indian Institute of Management, Indore, Madhya Pradesh, India; Institute of Social Sciences, Belgrade, Serbia; School of Economics and Finance and Centre for Behavioural Economics, Society and Technology (BEST), Queensland University of Technology, Brisbane City, Queensland, Australia; Department of Psychology, Royal Holloway, University of London, Egham, UK; Centre for the Politics of Feelings, School of Advanced Study, University of London, London, UK; Department of Political Science, National Taiwan University, Taipei, Taiwan; Faculty of Political Science, National University of Political Studies and Public Administration, Bucharest, Romania; Psychology Department, University of Latvia, Riga, Latvia; Department of Psychology, New York University, New York, NY, USA; School of Psychology, Speech, and Hearing, University of Canterbury, Christchurch, New Zealand; School of Economics and Management, Hebei University of Technology, Tianjin, China; School of Collective Intelligence, Mohammed VI Polytechnic University, Ben Guerir, Morocco; Department of Psychology, Hebrew University of Jerusalem, Jerusalem, Israel; Department of Neuroscience and Biomedical Engineering, Aalto University, Espoo, Finland; School of Collective Intelligence, Mohammed VI Polytechnic University, Ben Guerir, Morocco; Department of Psychology, Jose Rizal University, Mandaluyong, Metro Manila, Philippines; Department of Economics, Telenor Research, Fornebu, Norway; Cheikh Anta Diop University, Dakar, Senegal; School of Medicine and Health Sciences, Universidad del Rosario, Bogotá, Colombia; Melbourne School of Psychological Sciences, University of Melbourne, Melbourne, Victoria, Australia; Department of Digital Humanities, University of Helsinki, Helsinki, Finland; Faculty of Historical and Pedagogical Sciences, University of Wroclaw, Wroclaw, Poland; IMT School for Advanced Studies, Lucca, Italy; Laboratory for Psychology of Social Inequality, Higher School of Economics University, Moscow, Russia; Department of Economics and Management, University of Florence, Florence, Italy; Department of Management, London School of Economics and Political Science, London, UK; Department of Marketing, TBS Education, Toulouse, France; The Institute for Sociology, Slovak Academy of Sciences, Bratislava, Slovakia; Social Policy Institute, Ministry of Labor, Family and Social Affairs of the Slovak Republic, Bratislava, Slovakia; Department of Psychology, Federal University of Santa Catarina, Florianopolis, Brazil; Department of Economics, University of Bologna, Bologna, Italy; IUSS Cognitive Neuroscience Center, University School for Advanced Studies, Pavia, Italy; Central Department of Population Studies, Tribhuvan University, Kathmandu, Nepal; School of Psychology, Shenzhen University, Shenzhen, China; Department of Marketing, Darden School of Business, University of Virginia, Charlottesville, VA, USA; Department of Psychology, University of the Witwatersrand, Johannesburg, Republic of South Africa; MRC Social, Genetic and Developmental Psychiatry Centre,Institute for Advanced Study in Toulouse, Université Toulouse 1 Capitole, Toulouse Cedex 6, France; Faculty of Medicine, Pontifical Xavierian University, Bogotá, Colombia; U.O. Rho, Fondazione Luigi Clerici, Rho, Italy; Department of Management, Technology, and Economics, Swiss Federal Institute of Technology in Zürich, Zürich, Switzerland; School of Psychology, University of Birmingham, Birmingham, UK; School of Psychology, University of Oxford, Oxford, UK; Institute of Psychology, Center for Climate Action and Social Transformations, SWPS, University of Social Sciences and Humanities, Warsaw, Poland; Department of Trade and Market Institutions, Cracow University of Economics, Kraków, Poland; Institute of Health Policy, Management and Evaluation, Dalla Lana School of Public Health, University of Toronto, Toronto, Ontario, Canada; Department of Psychology, Western University, London, Ontario, Canada; Department of Psychology, University of California - Los Angeles, Los Angeles, CA, USA; Department of Psychology, Université Rennes 2, Rennes, France; Department of Communication Science, University of Amsterdam, Amsterdam, Netherlands; Université Clermont Auvergne LAPSCO, CNRS, Clermont-Ferrand, France; UBC Sauder School of Business, University of British Columbia, Vancouver, British Columbia, Canada; Teacher Education Department, Cavite State University, General Trias, Cavite, Philippines; IMT School for Advanced Studies, Lucca, Italy; School of Economics and Finance, Queensland University of Technology, Brisbane City, Queensland, Australia; Faculty of Education, Psychology and Art, University of Latvia, Riga, Latvia; Kieskompas (Election Compass), Amsterdam, Netherlands and Department of Political Science & Annenberg School for Communication, University of Pennsylvania, PA, USA; National Institute for the Intellectually Disabled and Autistic (NIIDA), Society for the Welfare of the Intellectually Disabled (SWID Bangladesh), Dhaka, Bangladesh; Department of Psychology, University of Münster, Münster, Germany; Department of Communication and Media Use, Friedrich Schiller University Jena, Jena, Germany; Department of Social Policy and Intervention, University of Oxford, Oxford, UK; Department of Media and Communications, University of Sydney, Sydney, New South Wales, Australia; Medical School, Department of Psychiatry, University of the Witwatersrand, Johannesburg, Republic of South Africa; Chair of Development Economics,University of Bayreuth, Bayreuth, Germany; Department of Psychology, University of Waterloo, Waterloo, Ontario, Canada; School of Psychology, Speech, and Hearing, University of Canterbury, Christchurch, New Zealand; Department of Nursing, University of Huelva, Huelva, Spain; Humanities and Social Sciences, Indian Institute of Management, Indore, Madhya Pradesh, India; Department of Psychology, University of North Carolina at Chapel Hill, Chapel Hill, NC, USA; Department of Psychology, University of Limerick, Limerick, Ireland; School of Psychology, University of Kent, Canterbury, UK; Department of Psychology and Neuroscience, College of Arts and Sciences, University of Colorado Boulder, Boulder, CO, USA; Department of Psychology, Hebrew University of Jerusalem, Jerusalem, Israel; Department of Digital Humanities, University of Helsinki, Helsinki, Finland; Institute of European Studies and International Relations, Faculty of Social and Economic Sciences, Comenius University, Bratislava, Slovakia; Department of Experimental Psychology, University of Regensburg, Regensburg, Germany; School of Economics and Finance, Queensland University of Technology, Brisbane City, Queensland, Australia; Department of Clinical Neuroscience, Karolinska Institutet, Stockholm, Sweden; Department of Political Science, Aarhus University, Aarhus, Denmark; Kyiv School of Economics, Kyiv, Ukraine; Department of Management and Engineering, Linköping University, Linköping, Sweden; Department of Digital Humanities, University of Helsinki, Helsinki, Finland; University of Helsinki, Helsinki, Finland; Turner Institute for Brain and Mental Health, Monash University, Clayton, Victoria, Australia; Department of Business and Media Psychology, Ansbach University of Applied Sciences, Ansbach, Germany; Center for Leadership and People Management, Ludwig Maximilian University of Munich, Munich, Germany; Department of Management, London School of Economics and Political Science, London, UK; Department of Marketing, Boston University, Questrom School of Business, Boston, MA, USA; School of Psychology, Dublin City University, Dublin, Ireland; Institute of Social Sciences Ivo Pilar, Zagreb, Croatia; Pontifical Xavierian University, Bogotá, Colombia; Seele Neuroscience, Mexico City, Mexico; COIDESO-Research Center of Contemporary Thinking and Innovation for Social Development, University of Huelva, Huelva, Spain; School of Psychology, University of Auckland, Auckland, New Zealand; Faculty of Economics, Maria Curie Sklodowska University, Lublin, Poland; Department of Psychology, City University of New York (CUNY) Graduate Center, New York, NY, USA; Department of Psychology, University of Alberta, Edmonton, Alberta, Canada; Department of Cognition, Emotion, and Methods in Psychology, University of Vienna, Wien, Austria; Australian National Centre for the Public Awareness of Science,Australian National University, Canberra ACT, Australia; Department of Psychology, University of Toronto, Toronto, Ontario, Canada; COIDESO-Research Center of Contemporary Thinking and Innovation for Social Development, University of Huelva, Huelva, Spain; Department of Psychology and Behavioral Sciences, Zhejiang University, Hangzhou, China; Department for Political Behavior, Centre for Social Sciences, Budapest, Hungary; Department of Clinical Neuroscience, Karolinska Institutet, Stockholm, Sweden; Institute of Psychology, University of Silesia in Katowice, Katowice, Poland; Facultad de Psicología,Complutense University of Madrid, Madrid, Spain; Department of Computer Science, University of Helsinki, Helsinki, Finland; Department of Psychology, University of the Witwatersrand, Johannesburg, Republic of South Africa; Department of Computer Science, Institute of Cognitive Science, University of Colorado Boulder, Boulder, CO, USA; Centro de Ciências Biológicas e da Saúde,Mackenzie Presbyterian University, São Paul, Brazil; Department of Psychology & Neural Science, New York University, New York, NY, USA; COIDESO-Research Center of Contemporary Thinking and Innovation for Social Development, University of Huelva, Huelva, Spain; Department of Biology, Biodiversity Unit, University of Turku, Turku, Finland; School of Psychology, Environmental Psychology Department, National Autonomous University of Mexico, Mexico City, Mexico; Centro de Ciências Biológicas e da Saúde,Mackenzie Presbyterian University, São Paul, Brazil; Department of Humanities and Life Sciences, University School for Advanced Studies, Pavia, Italy; Department of Social Psychology, University of Amsterdam, Amsterdam, Netherlands; Department of Psychology, University of the Witwatersrand, Johannesburg, Republic of South Africa; Department of Psychology, City University of New York (CUNY) Graduate Center, New York, NY, USA; Department of Global Economics and Management, University of Groningen, Groningen, Netherlands; Department of Psychology, Carleton University, Ottawa, Ontario, Canada; Department of Psychology & Neural Science, New York University, New York, NY, USA; PLS, ENS-Ulm, Department d’Etudes Cognitives, Paris, France; Africa Business School and The School of Collective Intelligence, UM6P, Rabat, Morocco; Department of Management, London School of Economics and Political Science, London, UK; Kyiv School of Economics, Kyiv, Ukraine; Social Psychology Department, University of Amsterdam, Amsterdam, Netherlands; Institute of Cognitive Neuroscience, University College London, London, UK; Department of Asian and International Studies, City University of Hong Kong, Kowloon Tong, Hong Kong; Department of Psychology, Susquehanna University, Selinsgrove, PA, USA; Department of Social Psychology, Friedrich Schiller University Jena, Jena, Germany; Department of Design, University of Antwerp, Antwerp, Belgium; Department of Behavioral Sciences and Learning, Linköping University, Linköping, Sweden; Department of Clinical Neuroscience, Karolinska Institutet, Stockholm, Sweden; Department of Psychology, University of Exeter, Exeter, UK; Department of Psychology, University of Koblenz-Landau, Landau, Germany; Medical School, Department of Psychiatry, University of the Witwatersrand, Johannesburg, Republic of South Africa; Department of Speech Pathology and Audiology, University of the Witwatersrand, Johannesburg, Republic of South Africa; Department of Management, Technology, and Economics, Swiss Federal Institute of Technology in Zürich, Zürich, Switzerland; Department of Psychology, Carleton University, Ottawa, Ontario, Canada; Institute of Psychology, Nicolaus Copernicus University, Torun, Poland; Rady School of Management, University of California, San Diego, CA, USA; Faculty of Arts and Science, Kyushu University, Fukuoka, Japan; Department of Psychology, Kadir Has University, Fatih, Istanbul, Turkey; School of Psychology, Speech, and Hearing, University of Canterbury, Christchurch, New Zealand; Department of Communication and Media Use, Friedrich Schiller University Jena, Jena, Germany; Department of Psychology, Erasmus University Rotterdam, Rotterdam, Netherlands; Centro de Ciências Biológicas e da Saúde,Mackenzie Presbyterian University, São Paul, Brazil; Faculty of Negotiations, Organizations and Markets, Harvard Business School, Boston, MA, USA; Department of Experimental and Applied Psychology, Vrije Universiteit Amsterdam, Amsterdam, Netherlands; Department of Economics, Vidyasagar College For Women, Kolkata, West Bengal, India; Department of Public Administration and Sociology, Erasmus University Rotterdam, Rotterdam, Netherlands; School of Collective Intelligence, Mohammed VI Polytechnic University, Ben Guerir, Morocco; Impact For Development, Casablanca, Morocco; Kieskompas (Election Compass), Amsterdam, Netherlands; Department of Psychology, University of Koblenz-Landau, Landau, Germany; Department of Economics, Koc University, Sarıyer, Istanbul, Turkey; Hult International Business School, Dubai, United Arab Emirates; Department of International Trade, Bogazici University, Besiktas, Istanbul, Turkey; Department of Psychological and Social Sciences, Penn State University Abington College, Abington, PA, USA; Department of Economics, Koc University, Sarıyer, Istanbul, Turkey; Department of Psychology & Neural Science, New York University, New York, NY, USA

**Keywords:** COVID-19, social distancing, hygiene, policy support, public health measures

## Abstract

At the beginning of 2020, COVID-19 became a global problem. Despite all the efforts to emphasize the relevance of preventive measures, not everyone adhered to them. Thus, learning more about the characteristics determining attitudinal and behavioral responses to the pandemic is crucial to improving future interventions. In this study, we applied machine learning on the multinational data collected by the International Collaboration on the Social and Moral Psychology of COVID-19 (*N* = 51,404) to test the predictive efficacy of constructs from social, moral, cognitive, and personality psychology, as well as socio-demographic factors, in the attitudinal and behavioral responses to the pandemic. The results point to several valuable insights. Internalized moral identity provided the most consistent predictive contribution—individuals perceiving moral traits as central to their self-concept reported higher adherence to preventive measures. Similar results were found for morality as cooperation, symbolized moral identity, self-control, open-mindedness, and collective narcissism, while the inverse relationship was evident for the endorsement of conspiracy theories. However, we also found a non-neglible variability in the explained variance and predictive contributions with respect to macro-level factors such as the pandemic stage or cultural region. Overall, the results underscore the importance of morality-related and contextual factors in understanding adherence to public health recommendations during the pandemic.

Significance Statement Outcomes of this study suggest that morality-related factors, along with prosociality and individual characteristics related to information processing and self-control, play an important role in determining attitudinal and behavioral responses to the COVID-19 pandemic. However, a substantial variation in the predictive contribution of included variables was observed. Therefore, the role of context (both in terms of culture and stage of the pandemic) should not be underestimated. Nevertheless, this study highlighted multiple factors relevant to the prevention of COVID-19 in different stages of the pandemic and cultures, which makes it a good starting point for more complex and causal research designs.

## Introduction

The COVID-19 pandemic has caused significant loss of life, commodities, jobs, and disruption of communities worldwide. As of March 2022, over 450 million infections and more than 6 million deaths have been reported globally (1). As we write this paper, the daily number of new cases worldwide exceeds one million. Given the lack of vaccination and treatment options, controlling the spread of the SARS-CoV-2 virus in its early stages depended on preventive behaviors, such as physical distancing (2) or hand and object disinfection (3, 4). While governments across the globe rushed to implement the proposed measures, many citizens resisted such change (5, 6). This is indicative of the vital role of individual characteristics in the form of attitudes, abilities, traits, and perceptions in compliance with preventive measures. Thus, decision-makers may benefit from insights from the social and behavioral sciences that could explain who will adhere to or ignore advised measures (7).

Furthermore, nations vary in the strictness of preventive measures enacted by local governments and the severity of the consequences of COVID-19: some countries report more than 100 deaths (e.g. Croatia and the UK), while others count less than one death (e.g. Bhutan and China) per 100,000 citizens (8). A recent cross-national analysis suggests that many of these excess deaths in countries like the United States are the result of weak public health infrastructure and a decentralized, inconsistent response to the pandemic (9). This raises questions of how macro-level cultural variables might be associated with citizens’ health attitudes and behaviors across nations.

The scientific community responded with numerous international research collaborations aimed at explaining adherence to preventive measures from different perspectives. One group of researchers (10) focused on cultural dimensions, self-awareness emotions, trust in governmental actions, and political orientation as predictors of compliance in the United States, Italy, and Korea. They found that horizontal collectivism was the only predictor of compliance significant in all three countries. Similarly, other scholars (11) identified collectivism's role in promoting preventive behaviors. In terms of adherence to preventive measures, prosocial tendencies emerged as a significant positive predictor, while perceiving others as violating preventive measures was the most consistent negative predictor (12). Results from another study across 70 countries showed that trust in government, conscientiousness, and agreeableness predicted engaging in preventive measures, with other variables having a negligible practical impact (13). As research accumulates, interpreting and integrating findings from diverse research streams with a variety of measures and samples presents another challenge for both scholars and practitioners.

Due to their freedom of theoretical constraints, data-driven approaches might offer solutions to “grand challenges” of existing theories, defined as complex problems with intertwined and evolving underlying mechanisms (14) as they allow the effective use of highly dimensional data (15). For instance, network analysis was used on data from the UK and the Netherlands to explore the relationship between multiple constructs relevant for COVID-19 attitudes and behaviors (16). The perceived level of adherence to norms and efficacy of preventive measures and support for these measures exhibited the strongest relationships with COVID-19 preventive behaviors. On the other hand, applying random forests on more than 100 potentially relevant variables established different descriptive and injunctive norms and prosociality as some of the most relevant predictors of behaviors (17). Overall, the relevance of prosociality and collectivism, both of which imply a willingness to make sacrifices for the benefit of the community, has been emphasized throughout the literature (10–12, 17–19). However, this does not eliminate the role of other individual differences and capacities in adherence to preventive measures (13, 17).

Our study expands on and contributes to the existing literature in three important ways. Firstly, we brought together a diverse team of experts to select several key constructs from social, moral, cognitive, and personality psychology that might be relevant to supporting public health recommendations. Despite a proliferation of studies on predictors of attitudinal and behavioral responses to the COVID-19 pandemic (see, for example, (20)), research in this field is still warranted. Hence, we sought to investigate attitudinal and behavioral responses in the first pandemic wave, when uncertainty regarding the spread of the virus dominated societies. In conjunction with the existing findings, our study provides valuable evidence which can be utilized to compare pandemic responses from different time points during the pandemic. Moreover, we sought to statistically test the association between the three related but distinct outcomes—maintaining physical hygiene, avoiding physical contact, and supporting governmental policies related to COVID-19. This distinguishes our approach from prior research that employed a general factor of preventive behaviors as it allowed us to gain insights both into attitudinal and behavioral responses to the measures aimed at mitigating the spread of the virus. Second, we consider potential cultural differences in the meaning of the studied constructs by establishing equivalence of factor scores through (partial) strong invariance (see (21)). Finally, to determine the efficacy of our independent variables in explaining contact avoidance, hygiene maintenance, and COVID-19 policy support in each country, we applied random forest-based regression algorithms appropriate for complex data sets with possible nonlinear and interactive relationships between variables (22, 23).

## Overview

We focused on two specific research questions utilizing a large international sample of 51,404 participants from 69 countries from all continents except Antarctica. First, we tested how precisely (in terms of the explained variance) avoiding contact, maintaining hygiene, and policy support could be predicted using a combination of variables from moral, social, personality, and cognitive psychology, as well as socio-demographic variables. Second, we tested which of the included variables provided a substantial contribution to the accuracy of our predictions. Descriptions of the expected effects (of all study variables) based on theories or earlier studies are available as Supplementary Materials A.(All supplementary files can be found online in the following OSF folder: https://osf.io/cvkyr/?view_only=c88c0431224c4f878750875e599d2983.) Additionally, to evaluate the robustness of our findings, we conducted additional analyses that took cultural differences and the pandemic stage during data collection into account.

## Materials and Methods

### International collaboration on the social and moral psychology of COVID-19 project

The aim of the International Collaboration on the Social and Moral Psychology of COVID-19 (ICSMP COVID-19) project is to examine and understand psychological factors related to the COVID-19 pandemic response. We launched the project in April 2020 via a social media call for national teams that could collect samples in their own country. Over 230 scholars responded to the call. The main questionnaire, created in English, was disseminated to each national team, responsible for translating it to their local language (using the standard forward–backwards method). Each team collected the data in their own country. The resulting datasets were then collated and analyzed altogether, and are available online (19, 24). The study received an umbrella ethics approval from the University of Kent.

### Participants

The analyzed sample consisted of 51,404 participants from 69 countries and territories, 25 of which collected samples representative of their respective nations regarding age and gender (*n* = 22,064). The remaining data were drawn from convenience samples. Following exclusion criteria set for the purposes of this study (we excluded participants providing inaccurate response to the attention check, participants who did not provide responses to more than one quarter of items, participants providing the same response more than ten times in a row on the items of our predictors, participants who chose “other” as their gender* and participants completing the questionnaire unusually fast or unusually slow, see Supplementary Materials B and C), 7,615 participants were removed, resulting in a sample of 43,789 (*M*_age_ = 43, SD_age_ = 16; 52% females) participants for our analyses (The number of participants who chose “other” as their gender was too low in the context of planned analyses).

### Measures

Unless otherwise indicated, participants responded on an 11-point scale with higher values indicating higher levels of the measured concepts (after reversing the appropriate items). Prior to conducting analyses that presumed grouping of participants, we achieved partial strong invariance for all of the included multi-item scales. This was important to ensure that we measured the same constructs with similar efficacy in each group (see (21)). Detailed output on how the fit was achieved can be found in Supplementary Material C.

#### Individual-level variables

##### Criteria

Avoidance of physical contact during the coronavirus (COVID-19) pandemic was measured via five items. Adequate fit (CFI = 0.979, RMSEA (95% CI upper limit) = 0.086, SRMR = 0.024, and ω2 = 0.69) was achieved after correlating the residuals of the last two items (keeping distance and avoiding handshakes).

Maintaining physical hygiene was measured via five items related to washing hands and other behaviors related to personal hygiene. A single factor structure was retained (CFI = 0.999, RMSEA (95% CI upper limit) = 0.037, SRMR = 0.007, and ω2 = 0.74) with correlated residuals of the first two items (washing hands longer and more thoroughly).

Support for COVID-19-related policy decisions was measured with five items relating to restrictive policies affecting five areas of everyday life. A single factor structure was retained (CFI = 0.989, RMSEA (95% CI upper limit) = 0.098, SRMR = 0.016, and ω2 = 0.86) after correlating residuals of support for forbidding public gatherings and unnecessary travel, and closing parks.

#### Predictors

##### Morality

Moral identity was measured using the 10-item moral identity scale (25). The original paper reports a two-factor model (Internalization and Symbolization), with acceptable internal consistency. The two-factor structure was confirmed in our study after correlating residuals of items 8 and 9, and 4 and 7 (CFI = 0.939, RMSEA (95% CI upper limit) = 0.084, SRMR = 0.067, ω2_internalization_ = 0.68, and ω2_symbolization_ = 0.75).

The moral circle scale (26) assesses the moral expansiveness across 16 different entities (human and nonhuman) deemed worthy of moral concern. Participants indicated the extent of their moral circle, i.e. the circle for which they are concerned about right and wrong done towards them, ranging from immediate family to all things in existence.

Morality as cooperation was measured using the Relevance subscale of the Morality-as-Cooperation Questionnaire (MAC-Q; (27)), which measures the extent to which each of the seven dimensions of cooperation is relevant when making moral judgments. One item per each of its seven dimensions was used in this study. After excluding the items of fairness and property and correlating residuals between *helping a family member* and *showing courage* and *helping a family member* and *uniting a community*, a general factor of the relevance of cooperation in morality (CFI = 0.991, RMSEA (95% CI upper limit) = 0.066, SRMR = 0.014, and ω2 = 0.73) was extracted.

##### (Pro)social identification and attitudes

National identity was assessed with two items combined into a scale: “I identify as [nationality]” (28) and “Being a [nationality] is an important reflection of who I am” (see (29)). The correlation among items was *r* = 0.69, and a single score was extracted using PAF.

Social belonging was measured using a four-item single-factor scale with excellent internal consistency (30). A single factor structure (CFI = 0.988, RMSEA (95% CI upper limit) = 0.115, SRMR = 0.017, and ω2 = 0.78) was confirmed in this study after correlating the residuals between first and third item.

Collective (national) narcissism was measured using three items of the original, single-factor Collective Narcissism scale (31). Invariance of this scale was tested along with the endorsement of COVID-19 conspiracy theories (32), which was measured using a single item for a denial conspiracy and three items for deflection conspiracies (e.g. “a hoax invented by interest groups for financial gains”). The three items related to collective narcissism and the four items related to belief in conspiracy theories were modeled together and yielded a clear two-factor structure (CFI = 0.988, RMSEA (95% CI upper limit) = 0.069, SRMR = 0.021, ω2_Conspiracies_ = 0.92, and ω2_Collective narcissism_ = 0.87).

Political orientation was measured using a single item, “Overall, what would be the best description of your political views?,” on a scale ranging from very left-leaning (“0”) to very right-leaning (“10”).

COVID-19 risk perception was measured with two items asking participants to rate how likely it was for them and for the average person to get infected with COVID-19 by 2021 April 30, on a slider scale ranging from 0 (“impossible”) to 100 (“certain”). Based on their high correlation (*r* = 0.66), a single component was extracted using PAF.

##### Individual dispositions

Individual grandiose narcissism was measured using the brief version of the Narcissistic Admiration and Rivalry Questionnaire (33), comprising two subcomponents, rivalry (R) and admiration (A), which exhibited acceptable internal consistency. The scale achieved acceptable fit after correlating residuals between items 3 and 6 reflecting rivalry (CFI = 0.986, RMSEA (95% CI upper limit) = 0.068, SRMR = 0.020, ω2 = 0.69 for admiration, and ω2 = 0.55 for rivalry).

Trait self-control was measured as a single-factor four-item scale (34), with the last two items being negatively worded. However, an adequate fit was not obtained even after correlating residuals of the first two items (CFI = 0.988, RMSEA (95% CI upper limit) = 0.115, SRMR = 0.017, and ω^2^ = 0.78).

Self-esteem was measured using the Single-Item Self-Esteem-Scale (SISE), which achieved good test–retest reliability and was established as a viable alternative of longer self-esteem scales (35).

Trait optimism was measured using two items from the three-item optimism subscale of the Life Orientation Test-Revised (36). Based on their high correlation (*r* = 0.71), a single factor was retrieved using PAF.

Open-mindedness, reflecting the acceptance of limitation of one's knowledge and willingness to gain new knowledge, was measured with a six-item scale of the Multidimensional measure of intellectual humility (37). The originally proposed single-factor structure achieved an acceptable fit in our study (CFI = 0.998, RMSEA (95% CI upper limit) = 0.025, and SRMR = 0.007) and was retained. It exhibited questionable internal consistency (ω2 = 0.50).

Cognitive reflection was measured with a three-item test that measures the ability to inhibit intuitive answers and engage in reflection to provide correct ones, adapted from Frederick (38). Correct answers were coded as “1” and incorrect as “0,” with a total scale ranging from 0 to 3.

##### Demographic factors and experiences

The MacArthur Scale of Subjective Social Status (39) was used to measure subjective socio-economic status by asking participants to place themselves on an 11-rung ladder, with the top rung representing individuals who are best off (in terms of education, jobs, and wealth), and the bottom rung the ones worst off.

Participants were asked whether they had (coded as “1”) tested positive for COVID-19 and/or had a close relative or acquaintance (friend, partner, family, colleague, and so on) who had tested positive for COVID-19 (“1”) or not (“0”) by the time of data collection.

Multiple demographic factors were also collected. Participants were asked to indicate whether they identify as “male,” “female,” or “other” and enter their age (in years). Additionally, participants’ marital status had the following three options: married, single, in a relationship (recoded into married or in a relationship (“1”) or other (“0”)), after which they indicated the number of children they had. Participants were also asked to indicate their employment status (recoded into the employed, students, or retired (“1”) or other (“0”)). Finally, participants indicated whether they lived in an urban (coded as “1”) or rural setting (coded as “0”).

### Analytical procedure

This study was not preregistered. Our analytical approach consisted of multiple steps (see Supplementary Materials B–F) conducted in R (40). A detailed description of data cleaning is presented in Supplementary Material B, while used packages are listed at the beginning of every Supplementary Material in which they were used.

The psychometric properties of the applied measures were tested on the imputed data (see Supplementary Material C). We focused on testing the applied measures’ factor structure and internal consistency. As the majority of the multi-item measures were taken from previously validated instruments, CFAs with robust maximum likelihood estimator (MLR; (41, 42)) and countries as clusters were applied using lavaan (43) to test whether the proposed structures fit to the overall data. Modification indices were consulted when theoretical models did not fit the data well.

We tested whether the obtained results were stable concerning the pandemic stage during data collection. In the absence of any specific criterion, we initially attempted to group countries according to the total number of COVID-19 cases per million inhabitants during the period of data collection, calculated as the average of the number of cases per million at the start date of data collection and the number of new cases per million at the end date of data collection.(https://github.com/CSSEGISandData/COVID-19.) In samples where only one date was provided, we used the available information for that date. However, we noticed an unwanted regularity in the grouping process—most countries with the total number of cases above the median were European countries, and no countries from Africa were in this group. Thus, to minimize potential cultural biases, we grouped the countries according to the Inglehart–Welzel cultural map (44) and selected the countries with the lowest and highest total number of cases per million from each cultural region (Orthodox European countries, Protestant European countries, Catholic European countries, English-Speaking countries, West and South Asian countries, Confucian countries, African–Islamic countries, and Latin American countries) as representative. This resulted in a group of countries in the early stage of the pandemic consisting of participants from Nigeria, Slovakia, Australia, Bulgaria, the Philippines, and Nepal. On the other hand, a group of countries in the advanced pandemic stage included participants from United Arab Emirates, Spain, Ireland, Serbia, Brazil, and Singapore. Regarding Latin American countries, we considered only countries with more than 150 participants as candidates, while no Protestant European countries were included due to all of them being in the advanced stage of the pandemic during the data collection period (with a total number of citizens infected per million exceeding 1,000). Our two groups were highly distinctive with respect to the total number of cases per million during the data collection (*M*_early stage_ = 154.14; *M*_advanced stage_ = 3520.87). In our attempt to further balance the analysis, we randomly selected the same number of participants from each selected country, equal to the size of the smallest included sample after the data cleaning (*n*_UAE_ = 176).

Then, we checked the cross-group invariance of our multi-item measures.(We also conducted analyses with groups reflecting regions of the Inglehart–Welzel cultural map (2020), which followed the described procedure. Due to space limitations, outputs of these analyses can be found in Supplementary Materials G.) After achieving an adequate fit by introducing changes suggested by modification indices, the cross-group invariance of each obtained theoretical model was tested. Stepwise tests were further conducted. First, configural models were formed for each construct, followed by models with constrained item loadings to test weak invariance, and ultimately models with constrained item loadings and intercepts to test strong invariance. If the configural model achieved adequate fit, successive changes in fit indices with respect to imposing restriction were used as a criterion for invariance. A CFI change of −0.015 accompanied by a change in RMSEA or SRMR of +0.015 was considered as an indication of achieving a higher level of invariance. If invariance was not achieved on the first attempt, modification indices were consulted to achieve partial invariance. Finally, we extracted factor scores from models reflecting strong invariance (where loadings and intercepts were constrained to form comparable scores across countries) using the ten Berge correction to use them in further analyses.

Because two-item measures cannot be tested using CFA, factor analyses using principal axis factoring were conducted to extract latent dimensions. In line with the factor scores based on strong invariance, the analyses were conducted on the entire dataset used in a specific analysis.

Socio-demographic characteristics and moral circle were not scaled. Variables absent from a specific national data set were replaced with a constant (i.e. the number of children in the Ghanaian data set was set to median of other countries, while the residence data was coded as urban for participants from Canada and Bulgaria).

The rest of the procedure was similar to the procedure applied by Van Lissa et al. (17). After data preparations, random forests were applied. Ranger function (45) was used to apply random forests that served as a basis for partial dependence plots and permutation importance metrics (see (46)), which were used to interpret the relationships (see Supplementary Material D). Regarding the hyperparameters, the number of trees was set to 1,000 and 2,000, *R*^2^ was chosen as the accuracy metric, permutation importance metrics were extracted as estimated variable importance, the number of variables to test at each split ranged from five to twenty with an increment of one, splitting was based on variance, while the minimum node sizes ranged from 3 to 99 with an increment of three. Holdout sample was used to ensure the robustness of findings: 20% of the sample from each country formed a test set on which *R*^2^ and variable importance metrics (see Supplementary Materials E and F) were calculated.

## Results

Obtained *R*^2^ values of optimally tuned models were of weak to moderate magnitude both on the complete data (*R*^2^_contact_ = 0.134, *R*^2^_hygiene_ = 0.200, and *R*^2^_policy_ = 0.146) and data consisting of samples nationally representative regarding age and gender (*R*^2^_contact_ = 0.172, *R*^2^_hygiene_ = 0.256, and *R*^2^_policy_ = 0.124). In the early stage of the pandemic, prediction of contact avoidance was negligible (*R^2^*_contact_ = 0.045, *R*^2^_hygiene_ = 0.272, and *R*^2^_policy_ = 0.138). On the other hand, in the advanced stage of the pandemic, our models led to a very imprecise prediction of maintaining hygiene (*R*^2^_contact_ = 0.129, *R*^2^_hygiene_ = −0.043, *R*^2^_policy_ = 0.173). Therefore, we decided not to interpret the predictive contributions in models with maintaining hygiene as the criterion on the sample reflecting the advanced stage of the pandemic. Nevertheless, they are presented in the following paragraphs.

**Fig. 1.**
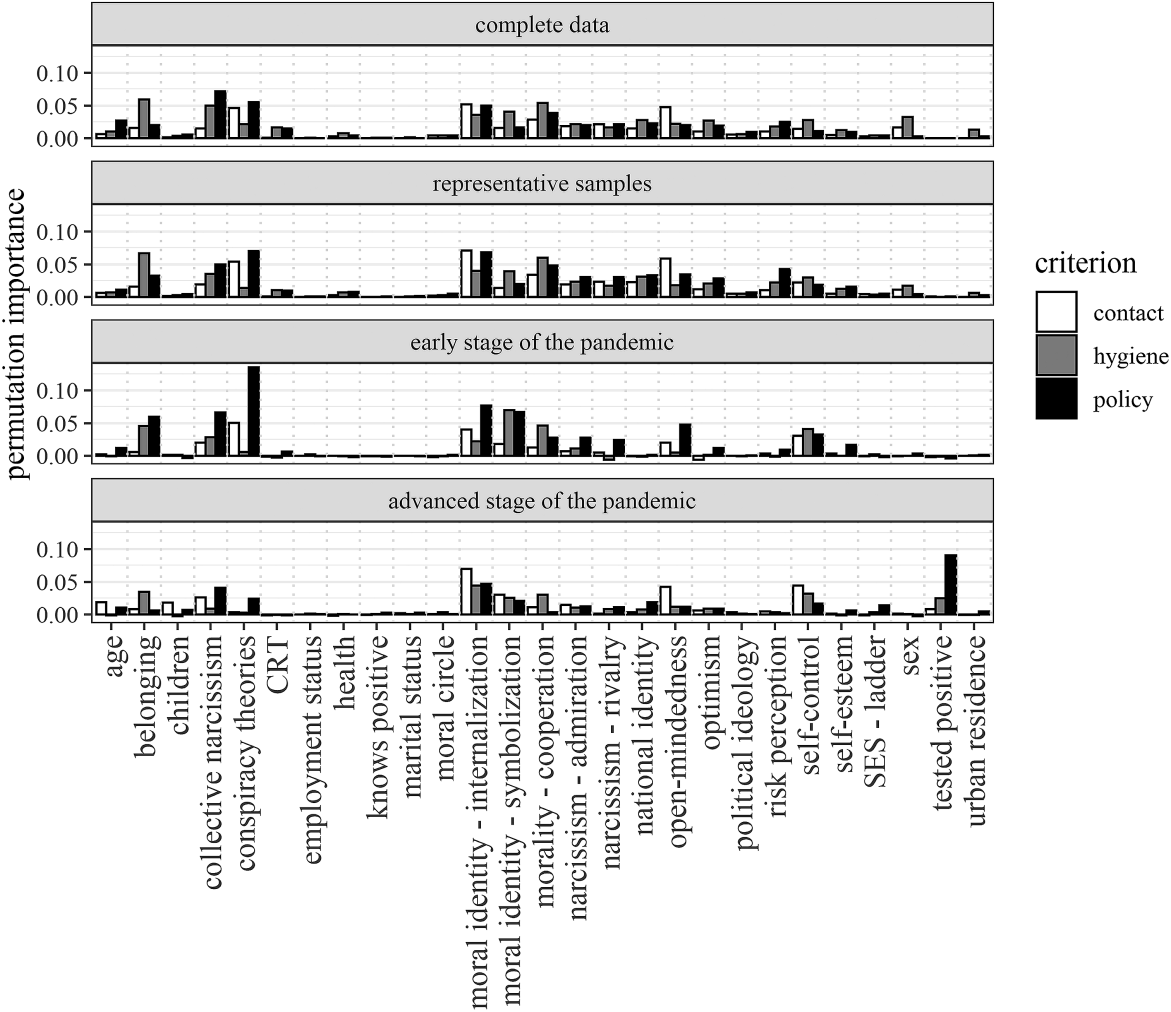
Permutation variable importance calculated with respect to representativeness of the samples and stage of the pandemic.

Results in Fig. 1 show the importance metrics based on the models that yielded the highest *R*^2^ per analysis. As permutation importance reflects a reduction in error, these plots are not directly comparable. However, some common patterns can be observed.

**Fig. 2.**
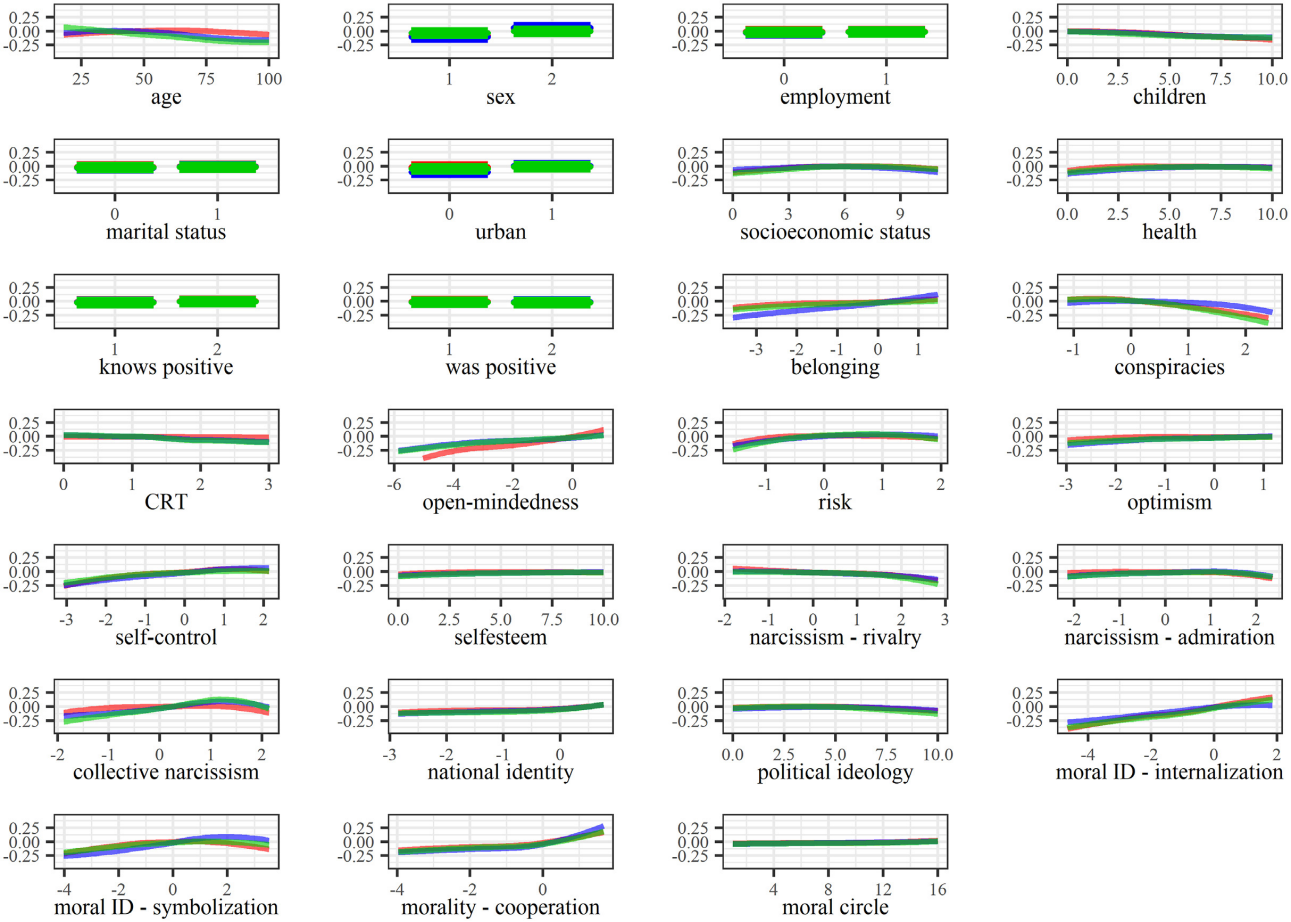
Partial dependence plots depicting the relationships between our predictors and criteria based on the complete data. Note: red, blue, and green represent avoiding contact, maintaining hygiene, and policy support, respectively.

In terms of avoiding contact (Fig. 2), internalized moral identity provided the most consistent contribution across analyses, followed by open-mindedness, collective narcissism, morality as cooperation, symbolized moral identity, and self-control. Endorsement of conspiracy theories seems to have exhibited a stronger relationship with our criteria in the early stage of the pandemic than in the advanced stage. In general, participants achieving higher scores on avoiding contact also achieved higher scores on internalized moral identity, morality as cooperation, self-control, and open-mindedness, respectively. These participants also exhibited lower endorsement of conspiracy theories. Regarding collective narcissism and symbolized moral identity, it seems that the individuals scoring around the midpoint reported higher contact avoidance compared to individuals scoring high and those scoring low on the scale.

**Fig. 3.**
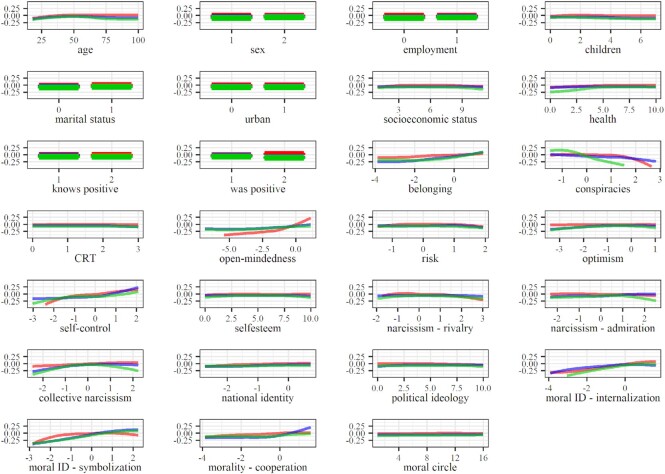
Partial dependence plots based on the data reflecting the early stage of pandemic. Note: red, blue, and green represent avoiding contact, maintaining hygiene, and policy support, respectively.

Regarding hygiene maintenance (Fig. 3), the most invariable contribution was found for social belonging and morality as cooperation, followed by internalized and symbolized moral identity, collective narcissism, and self-control. Gender differences in hygiene maintenance found on the complete data and data based on representative samples were not detected in data organized according to the stage of pandemic. Participants scoring higher on social belonging, internalized and symbolized moral identity, collective narcissism, and self-control also scored higher on maintaining hygiene. However, only the relationship between belonging and hygiene maintenance seemed linear—all other lines reached a plateau at some point (usually around the midpoint), indicating participants achieving the lowest scores on these factors also achieved the lowest scores on maintaining hygiene. On the other hand, higher scores were related to higher reported hygiene maintenance among participants scoring above the mean of morality as cooperation.

**Fig. 4.**
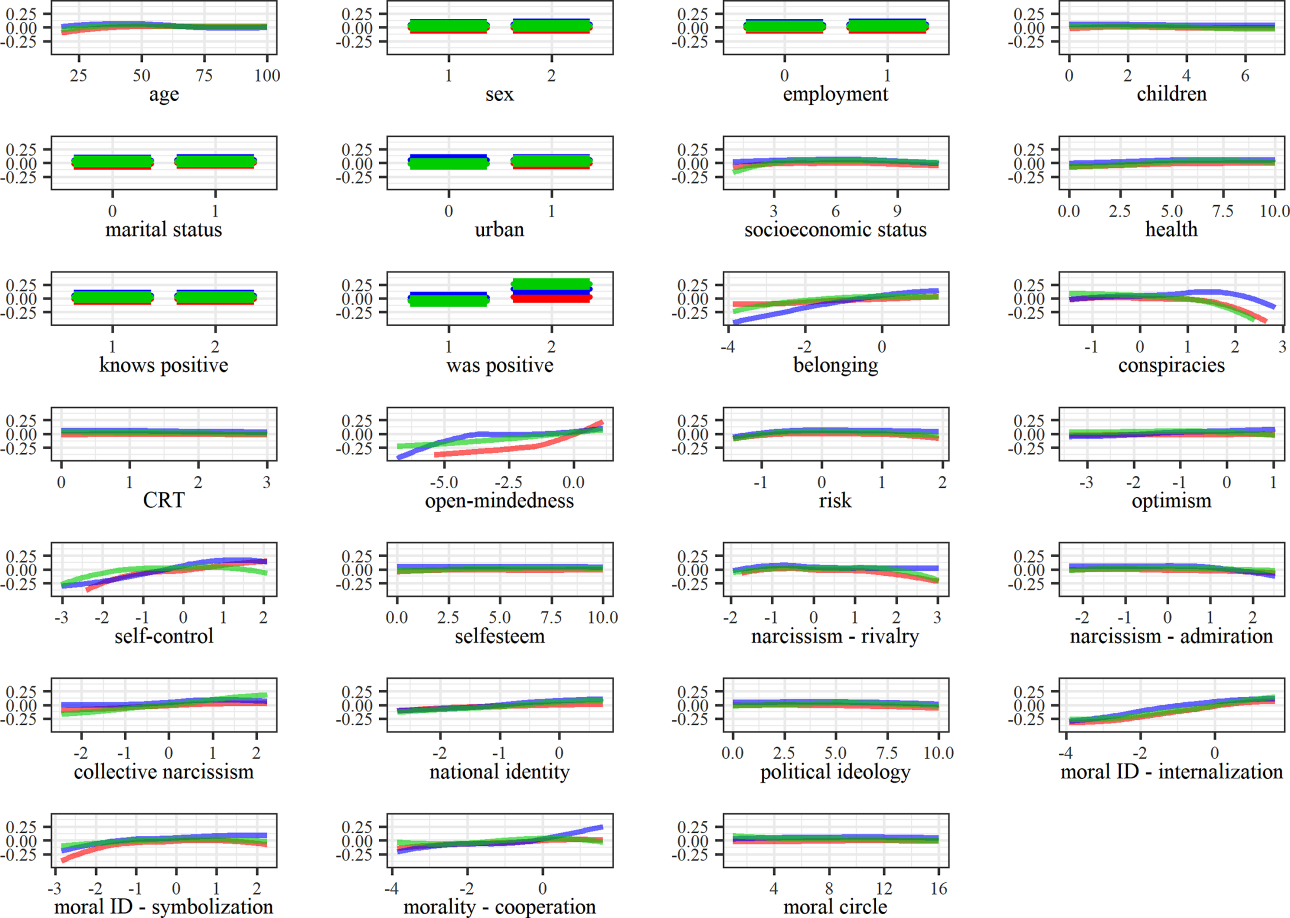
Partial dependence plots based on the data reflecting the advanced stage of pandemic. Note: red, blue, and green represent avoiding contact, maintaining hygiene, and policy support, respectively.

The most invariable predictors of policy support (Fig. 4) were collective narcissism, internalized moral identity, and self-control. Endorsement of conspiracy theories, symbolized moral identity, possibly even morality as cooperation, and open-mindedness, seem to have exhibited a stronger relationship with policy support in the early stages of the pandemic compared to the advanced stage. Participants scoring higher on internalized moral identity and self-control generally were also more supportive of policy measures. However, the relationships were not linear in the early pandemic stage (and in the advanced stage in the context of self-control). The relationship between policy support and collective narcissism was also complex—it was close to linear and positive in the advanced pandemic stage, but in the early stages and on the complete data, it resembled an inverted-U-curve with a peak around the mean. This indicates that participants scoring around the mean were most supportive of restrictive policies, while those high and the ones low on collective narcissism were less supportive. Participants showing more endorsement for COVID-19 conspiracy theories were less supportive, while participants scoring higher on open-mindedness were more supportive of restrictive COVID-19 policies. The relationship between morality as cooperation and policy support was established only on the complete data and indicated that only among those above the mean higher morality as cooperation was related to higher policy support. The opposite was found for symbolized moral identity—only among those lowest on this trait, the relationship between morality as cooperation and policy support was linear and positive. No relationships were established around the mean or above the mean.

## Discussion

Taking the machine learning approach, we provided several insights into social, psychological, personality, and cognitive factors in predicting COVID-19 responses. Although the nature of the analyses (i.e. the dependence of importance estimates on error estimates, which changes across models) prevents us from direct comparisons of results across models, some consistent patterns were observed.

Internalized moral identity was the most consistent predictor of COVID-19 attitudinal and behavioral responses—the extent to which people perceived moral traits as central to their self-concept was positively associated with their intentions to avoid physical contact, maintain hygiene, and support policy measures aimed at mitigating the spread of the virus. Morality-as-Cooperation was also associated with the attitudinal and behavioral responses, most consistently in predicting hygiene maintenance. These results suggest that maintaining hygiene, but also physical distancing and policy support, were perceived as collective actions that benefit the group more than they benefit the self. Symbolized moral identity was also associated with the criteria, but, interestingly, the relationship was nonlinear and strongest among participants scoring below the average of symbolized moral identity. These findings may reflect the fact that individuals characterized by moderate or high symbolization of moral identity prefer to be perceived as aligned with social norms, rather than actually adhering to them (47). However, the threshold at which the relationship becomes linear seems to change with respect to the pandemic stage and specific criteria, indicating the need for further research into these relationships.

Overall, these findings are in line with previous research suggesting that internalized moral identity is a relevant predictor of prosocial and cooperative intentions and behavior, with more inconsistent results when examining the symbolization dimension of moral identity (for a review, see (47, 48)). The only variable related to morality that did not substantially contribute to our criteria's prediction was the moral circle. Altogether, these results indicate that morality represents an important factor in adherence to preventive measures. Nevertheless, different aspects of morality provide different contributions to the prediction of adherence to these measures.

Open-mindedness and self-control were positively associated with avoiding contact and supporting policy, while self-control also exhibited a relatively steady, albeit weak, contribution to the prediction of hygiene maintenance. Open-mindedness was conceptualized as a part of cognitive humility, which reflects the virtue of being able to accept one's fallibility and the willingness to accept information contrary to one's initial beliefs (37, 49), with some authors treating it as a moral virtue (50, 51). Self-control is typically conceptualized as the capacity to work effectively to reach goals, resisting short-term temptations (34, 52). Some authors have suggested that self-control goals are often moralized (53). The relationship between open-mindedness and morality and between self-control and morality may underlie the predictive contribution of open-mindedness and self-control established in this study.

Social belonging was also established as a relevant predictor predominantly in terms of maintaining hygiene, while collective narcissism also provided a substantial contribution to predicting policy support and a less substantial contribution to predicting contact avoidance. On the one hand, ingroup identification promotes acceptance of group norms (54), implying that findings on social belonging could also reflect morality. On the other hand, the relationship between collective narcissism and our criteria seems to be more complex, in line with the mixed evidence of previous studies on the role of collective narcissism concerning various types of preventive behaviors such as handwashing, physical distancing, and limiting leaving home (32, 55). Namely, the evidence of a curvilinear relationship between collective narcissism and contact avoidance and policy support might reflect the need of individuals high in collective narcissism to establish and maintain a positive national image for the outside world (e.g. as model citizens, or morally superior) (56, 57). However, at even higher levels of collective narcissism, the need to assert and signal grandiosity and superiority in relation to various threats (in this case, the virus) might manifest in lower support for restrictive preventive measures, even at the cost of possible negative consequences for ingroup members (58, 59). This is also evident from the inverted-U curve in the context of policy support, usually appearing slightly above the mean (except in the late stage of the pandemic, see Figs. 2 and 3). Overall, this suggests that while believing in ingroup potential may motivate individuals to adhere to prosocial norms, irrational belief in superiority can undermine the support for preventive measures that bring about short-term disturbance in the everyday ingroup dynamics.

Additionally, conspiracy beliefs seem to be linked to contact avoidance and policy support, especially in the early stage of the pandemic. Namely, endorsement of COVID-19 conspiracy theories was associated with lower intentions to engage in physical distancing and lower policy support. Given that conspiracy believers were found to be more self-centered (60) and less generous (61) during the COVID-19 pandemic, this finding speaks in favor of viewing contact avoidance as a form of prosocial action.

The presented findings suggest that prosociality and morality are relevant factors for understanding physical distancing. This is in line with previous work on the role of prosociality on physical distancing (e.g. (10, 12, 17); see (18) for a review) and with the idea that personal norms, internal standards on what is right or wrong in a given situation, play an important role in driving prosocial behavior ((62, 63); see (64), for a review). However, our results indicated a substantial contextual variability, as well. While we focused on several most dependable and most substantial predictors only to describe general patterns, it should be noted that multiple other factors provided a contribution limited to a specific stage of the pandemic or specific culture (see Supplementary Materials G). This also implies that campaigns for increasing compliance with preventive measures in future crises should be tailored to both the pandemic phase and the specifics of the culture in which they plan to be implemented. Additionally, the results of our study suggest that psychological factors are more relevant than demographics in the context of health-related crises and should not be neglected when tailoring preventive measures.

Generally, the obtained *R*^2^ values were lower than those reported by Van Lissa et al. (17) in similar analyses. In their study, injunctive norms and support for COVID-19 restrictive measures were found to be two clearly dominant predictors of preventive behaviors, which may roughly approximate two aspects of the Theory of Planned Behavior (65)—subjective norms and attitudes on the specific behavior. We did not include these variables in our study, although policy support could broadly be considered as attitudes regarding preventive measures. Conversely, we treated policy support as one of the criteria rather than as one of the predictors, with contact avoidance, hygiene maintenance, and policy support being moderately correlated (*r* = approx. 0.40). Thus, the simplest explanation of the difference in the explained variance in our study compared to Van Lissa et al. (17) may reflect the difference in the extent to which the Theory of Planned Behavior has been represented among predictors. Considered together, the two studies provide evidence in favor of the Theory of Planned Behavior in the context of a global crisis.

Several limitations should be considered when interpreting our findings. First, not all the national samples were representative, and even the representative samples were not based on probabilistic sampling, and consequently, some segments of society may have been underrepresented. Furthermore, as the study was conducted online, our sample over-represents people with greater access to internet-enabled technology, which may be a particularly important consideration in less-developed countries (e.g. dissemination of conspiracy theories and fake news). Second, variability in our criteria was heavily skewed in many countries (i.e. the vast majority of participants reported high adherence to and support for preventive measures), which can be attributed to the first wave of the pandemic during which the data were collected. Nevertheless, in some countries, data collection was conducted during the peak of the first wave of the COVID-19 pandemic, while in other countries, it was carried out at its beginning. Although we tried to operationalize the pandemic stage according to the total number of infected individuals per million and took culture and sample size into account, even such operationalization may not have eliminated all the potential sources of bias. The rough similarity of the results based on representative and nonrepresentative data, as well as data from countries in different pandemic stages and data from countries grouped according to cultural zones (see Supplementary Materials G), provide arguments in favor of the validity of our findings; nevertheless, the robustness of these more specific findings needs to be corroborated utilizing different (i.e. longitudinal and nationally representative) samples. Furthermore, Morality-as-Cooperation had to be modeled differently than proposed in the original papers to achieve invariance. Additionally, as there are no conventional methods of testing the invariance of two-item and single-item measures across cultures, scores on such items may be less precisely calculated than in the case of multi-item measures. Finally, we focused on explaining variation in COVID-19 responses without testing causality. It should be noted that we used cross-sectional, self-reported data which may entail the desirability bias risk (66). However, there is evidence that such desirability bias does not play a key role, especially in self-reported measures like self-esteem, control, or optimism (67).

## Conclusion

Findings of our study indicate that the most effective predictors of COVID-19 responses, such as avoiding physical contact, maintaining hygiene, and supporting restrictive COVID-19 policies, were related to morality, prosociality, and traits and attitudes operationalizing self-control and information processing. However, the predictive contribution of even the most invariant predictors substantially varied with respect to the predicted type of response and cultural characteristics. While the research design of this study prevents any causal conclusions, the results suggest that the interplay between individual and contextual characteristics is relevant for understanding individual COVID-19 responses. Ultimately, our findings can serve as a starting point for future, more nuanced, research on the variables highlighted within our study. Hopefully, the growing body of research and accumulated insights should lead to informed and efficient prevention and intervention programs for health-related crises.

## Supplementary Material

pgac093_Supplemental_Files

## Data Availability

The data that support the findings of this study are openly available in OSF at http://doi.org/10.17605/osf.io/tfsza. These data have been used in multiple other manuscripts, including the “National identity predicts public health support during a global pandemic” manuscript, where Professor John Nezlek conducted the main analyses.
