# Supplementary material for: Predicting attitudinal and behavioral responses to COVID-19 pandemic using machine learning
Source: PNAS Nexus. 2022 Jul 5;1(3):pgac093. doi: 10.1093/pnasnexus/pgac093 (PMC9381137; doi:10.1093/pnasnexus/pgac093)
Supplement: pgac093_Supplemental_Files [file pgac093_supplemental_files.zip › Supplementary_materials_G._Analyses_with_respect_to_cultural_zones/Supplementary materials G. Outputs.html]

Cultural map


Code 

- Show All Code
- Hide All Code

# Cultural map

#### Deadpool

#### 27 02 2022

# Brief description

This file contains the outputs of random forests on the predictors of COVID-19 attitudes and behaviors. As such, it represents a follow-up to the file *Supplementary materials B. Data Cleaning*, also included in the supplementary materials. The quantity of data and analyses motivated us to split the procedure in several steps. This file contains all the steps relevant for analyses on countries grouped in line with Inglehart-Welzel cultural map. Therefore, first the information on **data preparations** are presented, followed by **random forests**, **variable importance plots** and **partial dependence plots**.

In order to reduce the size of the document, the codes are initially hidden. However, they can be shown by clicking on the specific line of code or by clicking on the button in the top right corner that shows/hides all codes.

# Data preparations

## Activating packages

In the following paragraph, packages with functions relevant for our analyses are activated.

```
sapply(c("dplyr", "psych", "lavaan", "semTools", "semPlot", "readr", "seminr"), library, character.only = T)
```

```
## 
## Attaching package: 'dplyr'
```

```
## The following objects are masked from 'package:stats':
## 
##     filter, lag
```

```
## The following objects are masked from 'package:base':
## 
##     intersect, setdiff, setequal, union
```

```
## This is lavaan 0.6-9
## lavaan is FREE software! Please report any bugs.
```

```
## 
## Attaching package: 'lavaan'
```

```
## The following object is masked from 'package:psych':
## 
##     cor2cov
```

```
##
```

```
## ###############################################################################
```

```
## This is semTools 0.5-5
```

```
## All users of R (or SEM) are invited to submit functions or ideas for functions.
```

```
## ###############################################################################
```

```
## 
## Attaching package: 'semTools'
```

```
## The following objects are masked from 'package:psych':
## 
##     reliability, skew
```

```
## 
## Attaching package: 'readr'
```

```
## The following object is masked from 'package:semTools':
## 
##     clipboard
```

```
## $dplyr
## [1] "dplyr"     "stats"     "graphics"  "grDevices" "utils"     "datasets" 
## [7] "methods"   "base"     
## 
## $psych
## [1] "psych"     "dplyr"     "stats"     "graphics"  "grDevices" "utils"    
## [7] "datasets"  "methods"   "base"     
## 
## $lavaan
##  [1] "lavaan"    "psych"     "dplyr"     "stats"     "graphics"  "grDevices"
##  [7] "utils"     "datasets"  "methods"   "base"     
## 
## $semTools
##  [1] "semTools"  "lavaan"    "psych"     "dplyr"     "stats"     "graphics" 
##  [7] "grDevices" "utils"     "datasets"  "methods"   "base"     
## 
## $semPlot
##  [1] "semPlot"   "semTools"  "lavaan"    "psych"     "dplyr"     "stats"    
##  [7] "graphics"  "grDevices" "utils"     "datasets"  "methods"   "base"     
## 
## $readr
##  [1] "readr"     "semPlot"   "semTools"  "lavaan"    "psych"     "dplyr"    
##  [7] "stats"     "graphics"  "grDevices" "utils"     "datasets"  "methods"  
## [13] "base"     
## 
## $seminr
##  [1] "seminr"    "readr"     "semPlot"   "semTools"  "lavaan"    "psych"    
##  [7] "dplyr"     "stats"     "graphics"  "grDevices" "utils"     "datasets" 
## [13] "methods"   "base"
```

## Basic description

In the following step, participants who were non-binary with respect to gender were excluded due to their low prevalence in our sample. After this exclusion, basic descriptive statistics of our sample are presented.

```
pls2 <- read_csv("finaldata_pmm.csv")
```

```
## New names:
## * `` -> ...1
```

```
## Rows: 43789 Columns: 92
```

```
## -- Column specification --------------------------------------------------------
## Delimiter: ","
## chr  (2): country, sample_coding
## dbl (90): ...1, age, att_check_nobots, children, cnarc1, cnarc2, cnarc3, con...
```

```
## 
## i Use `spec()` to retrieve the full column specification for this data.
## i Specify the column types or set `show_col_types = FALSE` to quiet this message.
```

```
pls2 <- pls2[order(pls2$country), ]
round(prop.table(table(pls2$sex1)), 2)
```

```
## 
##    1    2    3 
## 0.47 0.52 0.00
```

```
pls2 <- subset(pls2, sex1 < 3)
table(pls2$sex1)
```

```
## 
##     1     2 
## 20763 22888
```

```
describe(pls2$age)
```

```
##    vars     n  mean    sd median trimmed   mad min max range skew kurtosis   se
## X1    1 43651 43.33 16.03     42   42.69 19.27  18 100    82  0.3     -0.9 0.08
```

```
describe(pls2, IQR = T)
```

```
##                      vars     n     mean       sd median  trimmed      mad min
## ...1                    1 43651 21909.49 12641.26  21921 21912.68 16238.92   1
## age                     2 43651    43.33    16.03     42    42.69    19.27  18
## att_check_nobots        3 42967     1.00     0.00      1     1.00     0.00   1
## children                4 43651     1.11     1.30      1     0.93     1.48   0
## cnarc1                  5 43651     4.59     3.25      5     4.49     4.45   0
## cnarc2                  6 43651     5.32     3.03      5     5.39     2.97   0
## cnarc3                  7 43651     4.72     3.22      5     4.65     4.45   0
## contact1                8 43651     8.53     2.15      9     9.00     1.48   0
## contact2                9 43651     2.25     3.03      1     1.67     1.48   0
## contact3               10 43651     8.02     2.59      9     8.54     1.48   0
## contact4               11 43651     8.58     2.15      9     9.06     1.48   0
## contact5               12 43651     9.19     1.98     10     9.73     0.00   0
## CRT1                   13 43651     1.47     0.68      2     1.59     0.00   0
## CRT2                   14 43651     1.08     0.73      1     1.10     1.48   0
## CRT3                   15 43651     1.20     0.80      1     1.25     1.48   0
## ctheory1               16 43651     3.87     3.47      4     3.60     4.45   0
## ctheory2               17 43651     2.82     3.20      1     2.36     1.48   0
## ctheory3               18 43651     2.52     3.13      1     2.01     1.48   0
## ctheory4               19 43651     2.76     3.16      1     2.30     1.48   0
## duration               20 43651  1783.29  1553.60   1390  1497.29   652.34 434
## employ_status1         21 43651     2.72     1.86      2     2.53     1.48   1
## generosity1            22 43651    48.53    35.53     50    48.17    44.48   0
## generosity2            23 43651    34.13    29.44     30    30.54    29.65   0
## generosity3            24 43651    17.33    22.31     10    13.24    14.83   0
## happy                  25 43651     6.01     2.32      6     6.13     2.97   0
## health_cond            26 43651     7.03     2.09      7     7.18     1.48   0
## hygiene1               27 43651     8.16     2.31      9     8.59     1.48   0
## hygiene2               28 43651     8.29     2.25      9     8.72     1.48   0
## hygiene3               29 43651     8.84     2.01     10     9.31     0.00   0
## hygiene4               30 43651     6.44     3.34      7     6.79     4.45   0
## hygiene5               31 43651     8.15     2.55      9     8.67     1.48   0
## know_tested_positive   32 43651     1.20     0.40      1     1.13     0.00   1
## ladder                 33 43651     5.46     1.86      6     5.41     1.48   0
## marital1               34 43651     2.13     0.89      2     2.16     1.48   1
## mcoop1                 35 43651     6.97     2.51      7     7.27     2.97   0
## mcoop2                 36 43651     6.75     2.47      7     6.99     2.97   0
## mcoop3                 37 43651     7.75     2.26      8     8.06     2.97   0
## mcoop4                 38 43651     7.30     2.25      8     7.53     2.97   0
## mcoop5                 39 43651     5.69     2.69      6     5.81     2.97   0
## mcoop6                 40 43651     5.70     2.91      6     5.85     2.97   0
## mcoop7                 41 43651     5.68     3.45      6     5.85     4.45   0
## mor_circle             42 43651     9.44     5.24     10     9.66     7.41   1
## moralid1               43 43651     8.41     1.93      9     8.75     1.48   0
## moralid10              44 43651     7.38     2.49      8     7.70     2.97   0
## moralid2               45 43651     7.86     2.04      8     8.12     1.48   0
## moralid3               46 43651     4.63     2.91      5     4.59     2.97   0
## moralid4               47 43651     1.46     2.39      0     0.91     0.00   0
## moralid5               48 43651     5.99     2.58      6     6.15     2.97   0
## moralid6               49 43651     5.55     2.78      6     5.68     2.97   0
## moralid7               50 43651     2.84     2.98      2     2.43     2.97   0
## moralid8               51 43651     4.99     3.00      5     5.03     2.97   0
## moralid9               52 43651     5.36     2.92      6     5.48     2.97   0
## narc1                  53 43651     3.94     2.84      4     3.80     2.97   0
## narc2                  54 43651     4.71     2.87      5     4.71     2.97   0
## narc3                  55 43651     3.23     2.96      3     2.91     2.97   0
## narc4                  56 43651     5.53     2.87      6     5.66     2.97   0
## narc5                  57 43651     4.40     2.79      5     4.36     2.97   0
## narc6                  58 43651     2.97     2.87      2     2.64     2.97   0
## nidentity1             59 43651     8.42     2.46     10     8.96     0.00   0
## nidentity2             60 43651     7.23     3.05      8     7.71     2.97   0
## omind1                 61 43651     3.83     2.89      3     3.62     2.97   0
## omind2                 62 43651     8.72     2.09     10     9.21     0.00   0
## omind3                 63 43651     8.69     1.87      9     9.08     1.48   0
## omind4                 64 43651     8.67     1.86      9     9.05     1.48   0
## omind5                 65 43651     1.59     2.62      0     0.96     0.00   0
## omind6                 66 43651     2.54     2.72      2     2.14     2.97   0
## optim1                 67 43651     7.07     2.41      8     7.34     2.97   0
## optim2                 68 43651     7.28     2.33      8     7.54     2.97   0
## political_ideology     69 43651     4.93     2.30      5     4.92     1.48   0
## psupport1              70 43651     7.86     2.83      9     8.41     1.48   0
## psupport2              71 43651     8.23     2.62     10     8.80     0.00   0
## psupport3              72 43651     6.42     3.53      7     6.78     4.45   0
## psupport4              73 43651     8.64     2.30     10     9.19     0.00   0
## psupport5              74 43651     8.41     2.53     10     8.99     0.00   0
## riskperc1              75 43651    38.91    28.43     40    37.36    29.65   0
## riskperc2              76 43651    49.81    27.43     50    49.45    29.65   0
## sbelong1               77 43651     6.85     2.37      7     7.07     2.97   0
## sbelong2               78 43651     6.93     2.35      7     7.16     2.97   0
## sbelong3               79 43651     7.29     2.19      8     7.53     1.48   0
## sbelong4               80 43651     7.95     2.21      9     8.30     1.48   0
## self_esteem            81 43651     6.60     2.46      7     6.80     2.97   0
## sex1                   82 43651     1.52     0.50      2     1.53     0.00   1
## slf_ladder             83 43651     6.16     2.04      6     6.26     1.48   0
## slfcont1               84 43651     6.38     2.45      7     6.51     2.97   0
## slfcont2               85 43651     7.49     2.16      8     7.74     1.48   0
## slfcont3               86 43651     5.21     2.75      5     5.28     2.97   0
## slfcont4               87 43651     4.00     2.99      4     3.85     4.45   0
## tested_positive        88 43651     1.05     0.22      1     1.00     0.00   1
## urban                  89 43651     1.22     0.42      1     1.14     0.00   1
## revision_coding        90 43651     0.67     0.47      1     0.71     0.00   0
## country*               91 43651    27.37    16.61     27    27.21    22.24   1
## sample_coding*         92 43651     1.94     1.05      1     1.83     0.00   1
##                        max range  skew kurtosis    se   IQR
## ...1                 43789 43788  0.00    -1.20 60.51 21906
## age                    100    82  0.30    -0.90  0.08    26
## att_check_nobots         1     0   NaN      NaN  0.00     0
## children                10    10  1.38     3.31  0.01     2
## cnarc1                  10    10  0.03    -1.07  0.02     5
## cnarc2                  10    10 -0.25    -0.81  0.01     5
## cnarc3                  10    10 -0.01    -1.05  0.02     5
## contact1                10    10 -1.96     3.77  0.01     2
## contact2                10    10  1.27     0.35  0.01     4
## contact3                10    10 -1.48     1.41  0.01     3
## contact4                10    10 -2.07     4.30  0.01     2
## contact5                10    10 -3.16    10.08  0.01     1
## CRT1                     2     2 -0.91    -0.39  0.00     1
## CRT2                     2     2 -0.13    -1.13  0.00     1
## CRT3                     2     2 -0.38    -1.34  0.00     1
## ctheory1                10    10  0.35    -1.23  0.02     7
## ctheory2                10    10  0.83    -0.59  0.02     5
## ctheory3                10    10  1.01    -0.25  0.01     5
## ctheory4                10    10  0.85    -0.53  0.02     5
## duration             17846 17412  4.55    28.91  7.44   952
## employ_status1           6     5  0.52    -1.29  0.01     4
## generosity1            100   100 -0.02    -1.34  0.17    70
## generosity2            100   100  0.79    -0.20  0.14    40
## generosity3            100   100  1.69     2.94  0.11    25
## happy                   10    10 -0.49    -0.27  0.01     3
## health_cond             10    10 -0.73     0.15  0.01     2
## hygiene1                10    10 -1.56     2.31  0.01     3
## hygiene2                10    10 -1.69     2.84  0.01     3
## hygiene3                10    10 -2.24     5.19  0.01     2
## hygiene4                10    10 -0.62    -0.87  0.02     6
## hygiene5                10    10 -1.61     2.01  0.01     3
## know_tested_positive     2     1  1.47     0.15  0.00     0
## ladder                  11    11  0.25     0.28  0.01     2
## marital1                 3     2 -0.25    -1.68  0.00     2
## mcoop1                  10    10 -0.88     0.48  0.01     4
## mcoop2                  10    10 -0.74     0.29  0.01     4
## mcoop3                  10    10 -1.23     1.49  0.01     3
## mcoop4                  10    10 -0.92     0.83  0.01     3
## mcoop5                  10    10 -0.31    -0.47  0.01     4
## mcoop6                  10    10 -0.34    -0.71  0.01     4
## mcoop7                  10    10 -0.36    -1.18  0.02     6
## mor_circle              16    15 -0.16    -1.36  0.03    10
## moralid1                10    10 -1.56     2.87  0.01     2
## moralid10               10    10 -0.95     0.53  0.01     4
## moralid2                10    10 -1.16     1.61  0.01     3
## moralid3                10    10 -0.03    -0.85  0.01     5
## moralid4                10    10  1.85     2.69  0.01     2
## moralid5                10    10 -0.49    -0.21  0.01     3
## moralid6                10    10 -0.37    -0.55  0.01     4
## moralid7                10    10  0.87    -0.38  0.01     5
## moralid8                10    10 -0.24    -0.92  0.01     4
## moralid9                10    10 -0.34    -0.79  0.01     5
## narc1                   10    10  0.25    -0.90  0.01     4
## narc2                   10    10 -0.09    -0.86  0.01     5
## narc3                   10    10  0.60    -0.68  0.01     5
## narc4                   10    10 -0.37    -0.65  0.01     4
## narc5                   10    10  0.00    -0.86  0.01     4
## narc6                   10    10  0.66    -0.60  0.01     5
## nidentity1              10    10 -1.79     2.66  0.01     2
## nidentity2              10    10 -0.97    -0.10  0.01     5
## omind1                  10    10  0.45    -0.71  0.01     4
## omind2                  10    10 -2.40     6.11  0.01     2
## omind3                  10    10 -2.16     5.72  0.01     2
## omind4                  10    10 -1.97     4.66  0.01     2
## omind5                  10    10  1.86     2.52  0.01     2
## omind6                  10    10  0.98     0.04  0.01     5
## optim1                  10    10 -0.87     0.33  0.01     3
## optim2                  10    10 -0.89     0.51  0.01     3
## political_ideology      10    10  0.02    -0.14  0.01     3
## psupport1               10    10 -1.35     0.84  0.01     3
## psupport2               10    10 -1.62     1.83  0.01     3
## psupport3               10    10 -0.57    -1.07  0.02     6
## psupport4               10    10 -2.08     4.01  0.01     2
## psupport5               10    10 -1.82     2.60  0.01     2
## riskperc1              100   100  0.34    -0.90  0.14    50
## riskperc2              100   100  0.03    -0.90  0.13    40
## sbelong1                10    10 -0.77     0.23  0.01     4
## sbelong2                10    10 -0.82     0.34  0.01     4
## sbelong3                10    10 -0.96     0.80  0.01     3
## sbelong4                10    10 -1.31     1.51  0.01     3
## self_esteem             10    10 -0.70     0.01  0.01     3
## sex1                     2     1 -0.10    -1.99  0.00     1
## slf_ladder              10    10 -0.51     0.03  0.01     3
## slfcont1                10    10 -0.45    -0.44  0.01     3
## slfcont2                10    10 -0.94     0.67  0.01     3
## slfcont3                10    10 -0.23    -0.81  0.01     4
## slfcont4                10    10  0.26    -1.04  0.01     5
## tested_positive          2     1  3.98    13.88  0.00     0
## urban                    3     2  1.55     0.94  0.00     0
## revision_coding          1     1 -0.73    -1.46  0.00     1
## country*                55    54  0.08    -1.32  0.08    31
## sample_coding*           4     3  0.51    -1.25  0.01     2
```

## Forming groups and functions

The code chunks presented in this section allowed us to group countries with respect to the cultural map. Also, several functions from the package *seminr* were modified to allow multi-group extraction of ten Berge factor scores.

```
pls2$cgr <- ifelse(pls2$country == "MAR"| pls2$country == "ZAF"| pls2$country == "GHA"| pls2$country == "IRQ"| pls2$country == "NGA"| pls2$country == "PAK"| pls2$country == "SEN"| pls2$country == "ARE"| pls2$country == "TUR", "AFIS", ifelse(pls2$country == "AUT"| pls2$country == "BEL"| pls2$country == "HRV"| pls2$country == "FRA"| pls2$country == "HUN"| pls2$country == "ITA"| pls2$country == "ESP"| pls2$country == "POL"| pls2$country == "SVK", "CATEU", ifelse(pls2$country == "AUS"| pls2$country == "IRL"| pls2$country == "NZL"| pls2$country == "GBR"| pls2$country == "USA"| pls2$country == "CAN", "ES", ifelse(pls2$country == "ARG"| pls2$country == "BRA"| pls2$country == "BOL"| pls2$country == "CHL"| pls2$country == "COL"| pls2$country == "ECU"| pls2$country == "GTM"| pls2$country == "HND"| pls2$country == "MEX"| pls2$country == "PHL"| pls2$country == "PRI"| pls2$country == "CRI"| pls2$country == "NIC"| pls2$country == "CUB"| pls2$country == "PAN"| pls2$country == "PRY"| pls2$country == "URY"| pls2$country == "PER"| pls2$country == "SLV"| pls2$country == "VEN"| pls2$country == "DOM","LATAM", ifelse(pls2$country == "MKD"| pls2$country == "RUS"| pls2$country == "ROU"| pls2$country == "LVA"| pls2$country == "GRC"| pls2$country == "SRB"| pls2$country == "UKR"| pls2$country == "BGR", "ORT", ifelse(pls2$country =="DNK" | pls2$country == "FIN" | pls2$country =="DEU" | pls2$country == "NLD" | pls2$country =="NOR" | pls2$country =="SWE" | pls2$country =="CHE", "PROTEU", ifelse(pls2$country ==  "CHN"|pls2$country =="KOR"|pls2$country =="JPN"|pls2$country =="TWN", "CONF","SA")))))))
pls2 <- arrange(pls2, cgr)
pls2$fejk <- pls2$contact1

"%^%" <- function(S, power) with(eigen(S), vectors %*% (values ^ power * t(vectors)))

# ten Berge factor score calculator
calc_ten_berge_scores <- function(X, Lambda, Phi, i.means, i.sds) {
  if (any(is.na(X))) {
    # if any missing, impute using person average
    p.means <- rowMeans(X, na.rm = TRUE)
    missings <- which(is.na(X), arr.ind = TRUE)
    X[is.na(X)] <- p.means[missings[, 1]]
    X <- scale(X)
  } else {
    X <- t((t(X) - i.means) / i.sds)
  }
  R <- stats::cor(X, use = "pairwise")
  R.sqrt.i <- R %^% -0.5
  Phi.sqrt <- Phi %^% 0.5
  L <- Lambda %*% Phi.sqrt
  C <- R.sqrt.i %*% L %*% ((t(L) %*% chol2inv(chol(R)) %*% L) %^% -0.5)
  W <- R.sqrt.i %*% C %*% Phi.sqrt
  colnames(W) <- colnames(Lambda)
  rownames(W) <- rownames(Lambda)
  scores <- X %*% W
  colnames(scores) <- colnames(Lambda)
  list(scores = as.data.frame(scores))
}

estimate_lavaan_ten_berge_per_group <- function (fit) {
  Lambda_mats <- function(fit){
    longlist <- lavaan::lavInspect(fit, "std.lv")
    outputs <- list()
    for(i in seq_along(names(longlist))){
      outputs[[i]] <- eval(parse(text = paste0("longlist$", names(longlist)[i], "$lambda")))
    }
    outputs
  }
  Phi_mats <- function(fit){
    longlist2 <- lavaan::lavInspect(fit, what = "cor.lv")
    outputs <- list()
    for(i in seq_along(names(longlist2))){
      outputs[[i]] <- eval(parse(text = paste0("matrix(longlist2$", names(longlist2)[i], ", ncol(lambda_mat[[1]]))")))
    }
    outputs
  }
  X <- lavaan::lavInspect(fit, "data")
  i.means <- fit@SampleStats@mean
  i.sds <- lapply(fit@SampleStats@var, sqrt)
  lambda_mat <- Lambda_mats(fit)
  phi_mat <- Phi_mats(fit)
  ouut <- list()
  for(i in seq_along(X)){
    ouut[[i]] <- calc_ten_berge_scores(X[[i]], lambda_mat[[i]], phi_mat[[i]], i.means[[i]], i.sds[[i]])
  } 
  ouut
}
```

## Factor structure of multi-item measures

In the following subsection, we checked the factor structure of multi-item measures used in this study. Firstly the factor structure of our three criteria was evaluated (*contact*, *hygiene*, and *policy*), followed by the factor structure of our multi-item predictors.

After confirming the factor structure, we tested its invariance across cultural regions. We did this in a stepwise manner: firstly we formed a model reflecting *configural invariance* (i.e., all the items reflect the same constructs across groups), followed by models reflecting *weak* (i.e., factor loadings of items are similar across groups + configural invariance), and *strong* invariance (i.e., item intercepts are similar across groups + weak invariance). We compared these models in order to evaluate whether misfit introduced by placing constraints on the specific elements of the model was negligible. In case of non-begligible misfit, modification indices were consulted and theoretically plausible correlations among residuals or item intercepts were released of constraints in order to establish *partial invariance*. Interested readers can find out more about invariance here and here.

### Contact

```
pls2$contact2r <- 10-pls2$contact2
phc <- 'phc =~ contact1 + contact2r + contact3 + contact4 + contact5'
phymod <- cfa(phc, estimator = "MLR", data = pls2, std.lv = T, cluster = "country")
fitmeasures(phymod, fit.measures = c("chisq.scaled", "df.scaled", "cfi.robust", "rmsea.ci.upper.robust", "srmr"))
```

```
##          chisq.scaled             df.scaled            cfi.robust 
##             19673.454                 5.000                 0.912 
## rmsea.ci.upper.robust                  srmr 
##                 0.145                 0.047
```

```
miobj <- modindices(phymod)
top_n(miobj, 3, mi)
```

```
##        lhs op       rhs       mi   epc sepc.lv sepc.all sepc.nox
## 1 contact1 ~~ contact2r 1818.219 1.118   1.118    0.228    0.228
## 2 contact1 ~~  contact3 2120.006 1.005   1.005    0.290    0.290
## 3 contact4 ~~  contact5 3798.967 1.340   1.340    0.700    0.700
```

```
phc <- 'phc =~ contact3 + contact2r + contact1 + contact4 + contact5
        contact4 ~~ contact5'
phymod <- cfa(phc, estimator = "MLR", data = pls2, std.lv = T, cluster = "country")
fitmeasures(phymod, fit.measures = c("chisq.scaled", "df.scaled", "cfi.robust", "rmsea.ci.upper.robust", "srmr"))
```

```
##          chisq.scaled             df.scaled            cfi.robust 
##               237.970                 4.000                 0.979 
## rmsea.ci.upper.robust                  srmr 
##                 0.086                 0.024
```

```
semPaths(phymod, what = "std", edge.label.cex = 1.2, label.cex = 1.2)
```

```
reliability(phymod)
```

```
##              phc
## alpha  0.7161647
## omega  0.6854068
## omega2 0.6854068
## omega3 0.6881589
## avevar 0.3352971
```

```
phymodc <- cfa(phc, estimator = "MLR", data = pls2, std.lv = T, cluster = "country", group = "cgr")
```

```
## Warning in lav_model_vcov(lavmodel = lavmodel, lavsamplestats = lavsamplestats, : lavaan WARNING:
##     The variance-covariance matrix of the estimated parameters (vcov)
##     does not appear to be positive definite! The smallest eigenvalue
##     (= -1.841924e-15) is smaller than zero. This may be a symptom that
##     the model is not identified.
```

```
phymodw <- cfa(phc, estimator = "MLR", data = pls2, std.lv = T, cluster = "country", group = "cgr", group.equal = "loadings")
```

```
## Warning in lav_model_vcov(lavmodel = lavmodel, lavsamplestats = lavsamplestats, : lavaan WARNING:
##     The variance-covariance matrix of the estimated parameters (vcov)
##     does not appear to be positive definite! The smallest eigenvalue
##     (= -1.051124e-16) is smaller than zero. This may be a symptom that
##     the model is not identified.
```

```
phymods <- cfa(phc, estimator = "MLR", data = pls2, std.lv = T, cluster = "country", group = "cgr", group.equal = c("loadings", "intercepts"))
```

```
## Warning in lav_model_vcov(lavmodel = lavmodel, lavsamplestats = lavsamplestats, : lavaan WARNING:
##     The variance-covariance matrix of the estimated parameters (vcov)
##     does not appear to be positive definite! The smallest eigenvalue
##     (= 6.409785e-15) is close to zero. This may be a symptom that the
##     model is not identified.
```

```
summary(compareFit(phymodc, phymodw, phymods, nested = T))
```

```
## ################### Nested Model Comparison #########################
## Scaled Chi-Squared Difference Test (method = "satorra.bentler.2001")
## 
## lavaan NOTE:
##     The "Chisq" column contains standard test statistics, not the
##     robust test that should be reported per model. A robust difference
##     test is a function of two standard (not robust) statistics.
##  
##         Df    AIC    BIC  Chisq Chisq diff Df diff Pr(>Chisq)    
## phymodc 32 932883 933995 1188.6                                  
## phymodw 60 933713 934581 2074.1     114.06      28  2.385e-12 ***
## phymods 88 935967 936592 4384.5     109.23      28  1.540e-11 ***
## ---
## Signif. codes:  0 '***' 0.001 '**' 0.01 '*' 0.05 '.' 0.1 ' ' 1
## 
## ####################### Model Fit Indices ###########################
##         chisq.scaled df.scaled pvalue.scaled rmsea.robust cfi.robust tli.robust
## phymodc     710.502         32          .000        .081       .978†      .944 
## phymodw     459.338         60          .000        .074†      .965       .953†
## phymods     446.986†        88          .000        .086       .931       .937 
##          srmr         aic         bic
## phymodc .025† 932883.190† 933994.739†
## phymodw .046  933712.721  934581.119 
## phymods .066  935967.093  936592.340 
## 
## ################## Differences in Fit Indices #######################
##                   df.scaled rmsea.robust cfi.robust tli.robust  srmr      aic
## phymodw - phymodc        28       -0.006     -0.013      0.008 0.021  829.531
## phymods - phymodw        28        0.011     -0.034     -0.016 0.020 2254.372
##                        bic
## phymodw - phymodc  586.380
## phymods - phymodw 2011.221
```

```
miobj <- modindices(phymodw, free.remove = F)
miobj %>% filter(op == "=~") %>% group_by(lhs, rhs) %>% summarize(mi = mean(mi)) %>% top_n(3, mi)
```

```
## `summarise()` has grouped output by 'lhs'. You can override using the `.groups` argument.
```

```
## # A tibble: 3 x 3
## # Groups:   lhs [1]
##   lhs   rhs          mi
##   <chr> <chr>     <dbl>
## 1 phc   contact2r  14.5
## 2 phc   contact3   13.5
## 3 phc   contact5   31.7
```

```
phymodw <- cfa(phc, estimator = "MLR", data = pls2, std.lv = T, cluster = "country", group = "cgr", group.equal = "loadings", group.partial = c("phc =~ contact5"))
```

```
## Warning in lav_model_vcov(lavmodel = lavmodel, lavsamplestats = lavsamplestats, : lavaan WARNING:
##     The variance-covariance matrix of the estimated parameters (vcov)
##     does not appear to be positive definite! The smallest eigenvalue
##     (= -1.694018e-16) is smaller than zero. This may be a symptom that
##     the model is not identified.
```

```
phymods <- cfa(phc, estimator = "MLR", data = pls2, std.lv = T, cluster = "country", group = "cgr", group.equal = c("loadings", "intercepts"), group.partial = c("phc =~ contact5"))
```

```
## Warning in lav_model_vcov(lavmodel = lavmodel, lavsamplestats = lavsamplestats, : lavaan WARNING:
##     The variance-covariance matrix of the estimated parameters (vcov)
##     does not appear to be positive definite! The smallest eigenvalue
##     (= 6.850428e-15) is close to zero. This may be a symptom that the
##     model is not identified.
```

```
summary(compareFit(phymodc, phymodw, phymods, nested = T))
```

```
## ################### Nested Model Comparison #########################
## Scaled Chi-Squared Difference Test (method = "satorra.bentler.2001")
## 
## lavaan NOTE:
##     The "Chisq" column contains standard test statistics, not the
##     robust test that should be reported per model. A robust difference
##     test is a function of two standard (not robust) statistics.
##  
##         Df    AIC    BIC  Chisq Chisq diff Df diff Pr(>Chisq)    
## phymodc 32 932883 933995 1188.6                                  
## phymodw 53 933437 934366 1784.6     86.748      21  5.839e-10 ***
## phymods 81 935263 935949 3666.2     89.647      28  2.251e-08 ***
## ---
## Signif. codes:  0 '***' 0.001 '**' 0.01 '*' 0.05 '.' 0.1 ' ' 1
## 
## ####################### Model Fit Indices ###########################
##         chisq.scaled df.scaled pvalue.scaled rmsea.robust cfi.robust tli.robust
## phymodc     710.502         32          .000        .081       .978†      .944 
## phymodw     478.127         53          .000        .074†      .969       .953†
## phymods     378.045†        81          .000        .081       .944       .944 
##          srmr         aic         bic
## phymodc .025† 932883.190† 933994.739†
## phymodw .040  933437.228  934366.414 
## phymods .057  935262.877  935948.912 
## 
## ################## Differences in Fit Indices #######################
##                   df.scaled rmsea.robust cfi.robust tli.robust  srmr      aic
## phymodw - phymodc        21       -0.007     -0.009      0.009 0.014  554.038
## phymods - phymodw        28        0.007     -0.025     -0.009 0.017 1825.650
##                        bic
## phymodw - phymodc  371.674
## phymods - phymodw 1582.498
```

```
miobj <- modindices(phymods, free.remove = F)
miobj %>% filter(op == "~1") %>% group_by(lhs, rhs) %>% summarize(mi = mean(mi)) %>% top_n(3, mi)
```

```
## `summarise()` has grouped output by 'lhs'. You can override using the `.groups` argument.
```

```
## # A tibble: 6 x 3
## # Groups:   lhs [6]
##   lhs       rhs      mi
##   <chr>     <chr> <dbl>
## 1 contact1  ""     50.2
## 2 contact2r ""     50.7
## 3 contact3  ""     23.9
## 4 contact4  ""     32.3
## 5 contact5  ""     43.5
## 6 phc       ""     57.7
```

```
phymods <- cfa(phc, estimator = "MLR", data = pls2, std.lv = T, cluster = "country", group = "cgr", group.equal = c("loadings", "intercepts"), group.partial = c("phc =~ contact5", "contact2r ~ 1"))
```

```
## Warning in lav_model_vcov(lavmodel = lavmodel, lavsamplestats = lavsamplestats, : lavaan WARNING:
##     The variance-covariance matrix of the estimated parameters (vcov)
##     does not appear to be positive definite! The smallest eigenvalue
##     (= 2.661994e-15) is close to zero. This may be a symptom that the
##     model is not identified.
```

```
summary(compareFit(phymodc, phymodw, phymods, nested = T))
```

```
## ################### Nested Model Comparison #########################
## Scaled Chi-Squared Difference Test (method = "satorra.bentler.2001")
## 
## lavaan NOTE:
##     The "Chisq" column contains standard test statistics, not the
##     robust test that should be reported per model. A robust difference
##     test is a function of two standard (not robust) statistics.
##  
##         Df    AIC    BIC  Chisq Chisq diff Df diff Pr(>Chisq)    
## phymodc 32 932883 933995 1188.6                                  
## phymodw 53 933437 934366 1784.6     86.748      21  5.839e-10 ***
## phymods 74 934857 935604 3246.6    135.380      21  < 2.2e-16 ***
## ---
## Signif. codes:  0 '***' 0.001 '**' 0.01 '*' 0.05 '.' 0.1 ' ' 1
## 
## ####################### Model Fit Indices ###########################
##         chisq.scaled df.scaled pvalue.scaled rmsea.robust cfi.robust tli.robust
## phymodc     710.502         32          .000        .081       .978†      .944 
## phymodw     478.127†        53          .000        .074†      .969       .953†
## phymods     565.816         74          .000        .084       .945       .940 
##          srmr         aic         bic
## phymodc .025† 932883.190† 933994.739†
## phymodw .040  933437.228  934366.414 
## phymods .052  934857.252  935604.074 
## 
## ################## Differences in Fit Indices #######################
##                   df.scaled rmsea.robust cfi.robust tli.robust  srmr      aic
## phymodw - phymodc        21       -0.007     -0.009      0.009 0.014  554.038
## phymods - phymodw        21        0.010     -0.024     -0.013 0.013 1420.024
##                        bic
## phymodw - phymodc  371.674
## phymods - phymodw 1237.660
```

```
miobj <- modindices(phymods, free.remove = F)
miobj %>% filter(op == "~1") %>% group_by(lhs, rhs) %>% summarize(mi = mean(mi)) %>% top_n(3, mi)
```

```
## `summarise()` has grouped output by 'lhs'. You can override using the `.groups` argument.
```

```
## # A tibble: 6 x 3
## # Groups:   lhs [6]
##   lhs       rhs         mi
##   <chr>     <chr>    <dbl>
## 1 contact1  ""    5.99e+ 1
## 2 contact2r ""    6.56e-11
## 3 contact3  ""    2.40e+ 1
## 4 contact4  ""    2.79e+ 1
## 5 contact5  ""    4.28e+ 1
## 6 phc       ""    5.68e+ 1
```

```
phymods <- cfa(phc, estimator = "MLR", data = pls2, std.lv = T, cluster = "country", group = "cgr", group.equal = c("loadings", "intercepts"), group.partial = c("phc =~ contact5", "contact2r ~ 1", "contact1 ~ 1"))
```

```
## Warning in lav_model_vcov(lavmodel = lavmodel, lavsamplestats = lavsamplestats, : lavaan WARNING:
##     The variance-covariance matrix of the estimated parameters (vcov)
##     does not appear to be positive definite! The smallest eigenvalue
##     (= 7.789788e-16) is close to zero. This may be a symptom that the
##     model is not identified.
```

```
summary(compareFit(phymodc, phymodw, phymods, nested = T))
```

```
## ################### Nested Model Comparison #########################
## Scaled Chi-Squared Difference Test (method = "satorra.bentler.2001")
## 
## lavaan NOTE:
##     The "Chisq" column contains standard test statistics, not the
##     robust test that should be reported per model. A robust difference
##     test is a function of two standard (not robust) statistics.
##  
##         Df    AIC    BIC  Chisq Chisq diff Df diff Pr(>Chisq)    
## phymodc 32 932883 933995 1188.6                                  
## phymodw 53 933437 934366 1784.6     86.748      21  5.839e-10 ***
## phymods 67 934266 935074 2641.7     74.536      14  2.878e-10 ***
## ---
## Signif. codes:  0 '***' 0.001 '**' 0.01 '*' 0.05 '.' 0.1 ' ' 1
## 
## ####################### Model Fit Indices ###########################
##         chisq.scaled df.scaled pvalue.scaled rmsea.robust cfi.robust tli.robust
## phymodc     710.502         32          .000        .081       .978†      .944 
## phymodw     478.127†        53          .000        .074†      .969       .953†
## phymods     493.283         67          .000        .079       .955       .947 
##          srmr         aic         bic
## phymodc .025† 932883.190† 933994.739†
## phymodw .040  933437.228  934366.414 
## phymods .047  934266.300  935073.910 
## 
## ################## Differences in Fit Indices #######################
##                   df.scaled rmsea.robust cfi.robust tli.robust  srmr     aic
## phymodw - phymodc        21       -0.007     -0.009      0.009 0.014 554.038
## phymods - phymodw        14        0.005     -0.014     -0.006 0.008 829.072
##                       bic
## phymodw - phymodc 371.674
## phymods - phymodw 707.496
```

```
physicals <- bind_rows(estimate_lavaan_ten_berge_per_group(phymods))
```

### Hygiene

```
phg <- 'phg =~ hygiene1 + hygiene2 + hygiene3 + hygiene4 + hygiene5'
phgmod <- cfa(phg, estimator = "MLR", data = pls2, std.lv = T, cluster = "country")
fitmeasures(phgmod, fit.measures = c("chisq.scaled", "df.scaled", "cfi.robust", "rmsea.ci.upper.robust", "srmr"))
```

```
##          chisq.scaled             df.scaled            cfi.robust 
##               518.918                 5.000                 0.952 
## rmsea.ci.upper.robust                  srmr 
##                 0.148                 0.051
```

```
reliability(phgmod)
```

```
##              phg
## alpha  0.7846304
## omega  0.7750037
## omega2 0.7750037
## omega3 0.7378972
## avevar 0.4245873
```

```
miobj <- modindices(phgmod)
top_n(miobj, 3, mi)
```

```
##        lhs op      rhs       mi   epc sepc.lv sepc.all sepc.nox
## 1 hygiene1 ~~ hygiene2 4525.982 2.601   2.601    2.783    2.783
## 2 hygiene3 ~~ hygiene4 1815.034 1.023   1.023    0.213    0.213
## 3 hygiene4 ~~ hygiene5 1274.950 1.191   1.191    0.176    0.176
```

```
phg <- 'phg =~ hygiene1 + hygiene2 + hygiene3 + hygiene4 + hygiene5
        hygiene1 ~~ hygiene2'
phgmod <- cfa(phg, estimator = "MLR", data = pls2, std.lv = T, cluster = "country")
fitmeasures(phgmod, fit.measures = c("chisq.scaled", "df.scaled", "cfi.robust", "rmsea.ci.upper.robust", "srmr"))
```

```
##          chisq.scaled             df.scaled            cfi.robust 
##                17.696                 4.000                 0.999 
## rmsea.ci.upper.robust                  srmr 
##                 0.037                 0.007
```

```
semPaths(phgmod, what = "std", edge.label.cex = 1.2, label.cex = 1.2)
```

```
reliability(phgmod)
```

```
##              phg
## alpha  0.7846304
## omega  0.7389305
## omega2 0.7389305
## omega3 0.7383237
## avevar 0.4046403
```

```
phgmodc <- cfa(phg, estimator = "MLR", data = pls2, std.lv = T, cluster = "country", group = "cgr")
```

```
## Warning in lav_model_vcov(lavmodel = lavmodel, lavsamplestats = lavsamplestats, : lavaan WARNING:
##     The variance-covariance matrix of the estimated parameters (vcov)
##     does not appear to be positive definite! The smallest eigenvalue
##     (= -1.733697e-15) is smaller than zero. This may be a symptom that
##     the model is not identified.
```

```
phgmodw <- cfa(phg, estimator = "MLR", data = pls2, std.lv = T, cluster = "country", group = "cgr", group.equal = "loadings")
```

```
## Warning in lav_model_vcov(lavmodel = lavmodel, lavsamplestats = lavsamplestats, : lavaan WARNING:
##     The variance-covariance matrix of the estimated parameters (vcov)
##     does not appear to be positive definite! The smallest eigenvalue
##     (= 9.764892e-18) is close to zero. This may be a symptom that the
##     model is not identified.
```

```
phgmods <- cfa(phg, estimator = "MLR", data = pls2, std.lv = T, cluster = "country", group = "cgr", group.equal = c("loadings", "intercepts"))
```

```
## Warning in lav_model_vcov(lavmodel = lavmodel, lavsamplestats = lavsamplestats, : lavaan WARNING:
##     The variance-covariance matrix of the estimated parameters (vcov)
##     does not appear to be positive definite! The smallest eigenvalue
##     (= 6.191373e-15) is close to zero. This may be a symptom that the
##     model is not identified.
```

```
summary(compareFit(phgmodc, phgmodw, phgmods, nested = T))
```

```
## ################### Nested Model Comparison #########################
## Scaled Chi-Squared Difference Test (method = "satorra.bentler.2001")
## 
## lavaan NOTE:
##     The "Chisq" column contains standard test statistics, not the
##     robust test that should be reported per model. A robust difference
##     test is a function of two standard (not robust) statistics.
##  
##         Df    AIC    BIC  Chisq Chisq diff Df diff Pr(>Chisq)    
## phgmodc 32 909993 911104  421.5                                  
## phgmodw 60 910613 911482 1098.1     95.045      28  3.171e-09 ***
## phgmods 88 914999 915625 5540.2    188.535      28  < 2.2e-16 ***
## ---
## Signif. codes:  0 '***' 0.001 '**' 0.01 '*' 0.05 '.' 0.1 ' ' 1
## 
## ####################### Model Fit Indices ###########################
##         chisq.scaled df.scaled pvalue.scaled rmsea.robust cfi.robust tli.robust
## phgmodc     157.734†        32          .000        .044†     0.996†      .990†
## phgmodw     231.312         60          .000        .050       .990       .987 
## phgmods     516.160         88          .000        .098       .946       .951 
##          srmr         aic         bic
## phgmodc .013† 909992.737† 911104.286†
## phgmodw .036  910613.326  911481.724 
## phgmods .071  914999.478  915624.724 
## 
## ################## Differences in Fit Indices #######################
##                   df.scaled rmsea.robust cfi.robust tli.robust  srmr      aic
## phgmodw - phgmodc        28        0.006     -0.006     -0.003 0.023  620.589
## phgmods - phgmodw        28        0.048     -0.044     -0.036 0.035 4386.152
##                        bic
## phgmodw - phgmodc  377.438
## phgmods - phgmodw 4143.000
```

```
miobj <- modindices(phgmods, free.remove = F)
miobj %>% filter(op == "~1") %>% group_by(lhs, rhs) %>% summarize(mi = mean(mi)) %>% top_n(3, mi)
```

```
## `summarise()` has grouped output by 'lhs'. You can override using the `.groups` argument.
```

```
## # A tibble: 6 x 3
## # Groups:   lhs [6]
##   lhs      rhs        mi
##   <chr>    <chr>   <dbl>
## 1 hygiene1 ""    9.32e+0
## 2 hygiene2 ""    1.02e+1
## 3 hygiene3 ""    1.22e+2
## 4 hygiene4 ""    2.88e+2
## 5 hygiene5 ""    1.11e+2
## 6 phg      ""    7.89e-9
```

```
phgmods <- cfa(phg, estimator = "MLR", data = pls2, std.lv = T, cluster = "country", group = "cgr", group.equal = c("loadings", "intercepts"), group.partial = c("hygiene4 ~ 1"))
```

```
## Warning in lav_model_vcov(lavmodel = lavmodel, lavsamplestats = lavsamplestats, : lavaan WARNING:
##     The variance-covariance matrix of the estimated parameters (vcov)
##     does not appear to be positive definite! The smallest eigenvalue
##     (= 1.827581e-15) is close to zero. This may be a symptom that the
##     model is not identified.
```

```
summary(compareFit(phgmodc, phgmodw, phgmods, nested = T))
```

```
## ################### Nested Model Comparison #########################
## Scaled Chi-Squared Difference Test (method = "satorra.bentler.2001")
## 
## lavaan NOTE:
##     The "Chisq" column contains standard test statistics, not the
##     robust test that should be reported per model. A robust difference
##     test is a function of two standard (not robust) statistics.
##  
##         Df    AIC    BIC  Chisq Chisq diff Df diff Pr(>Chisq)    
## phgmodc 32 909993 911104  421.5                                  
## phgmodw 60 910613 911482 1098.1     95.045      28  3.171e-09 ***
## phgmods 81 912216 912902 2742.5     88.812      21  2.585e-10 ***
## ---
## Signif. codes:  0 '***' 0.001 '**' 0.01 '*' 0.05 '.' 0.1 ' ' 1
## 
## ####################### Model Fit Indices ###########################
##         chisq.scaled df.scaled pvalue.scaled rmsea.robust cfi.robust tli.robust
## phgmodc     157.734†        32          .000        .044†     0.996†      .990†
## phgmodw     231.312         60          .000        .050       .990       .987 
## phgmods     329.755         81          .000        .068       .976       .976 
##          srmr         aic         bic
## phgmodc .013† 909992.737† 911104.286†
## phgmodw .036  910613.326  911481.724 
## phgmods .049  912215.763  912901.798 
## 
## ################## Differences in Fit Indices #######################
##                   df.scaled rmsea.robust cfi.robust tli.robust  srmr      aic
## phgmodw - phgmodc        28        0.006     -0.006     -0.003 0.023  620.589
## phgmods - phgmodw        21        0.019     -0.015     -0.011 0.014 1602.437
##                        bic
## phgmodw - phgmodc  377.438
## phgmods - phgmodw 1420.074
```

```
miobj <- modindices(phgmods, free.remove = F)
miobj %>% filter(op == "~1") %>% group_by(lhs, rhs) %>% summarize(mi = mean(mi)) %>% top_n(3, mi)
```

```
## `summarise()` has grouped output by 'lhs'. You can override using the `.groups` argument.
```

```
## # A tibble: 6 x 3
## # Groups:   lhs [6]
##   lhs      rhs        mi
##   <chr>    <chr>   <dbl>
## 1 hygiene1 ""    1.06e+1
## 2 hygiene2 ""    3.94e+0
## 3 hygiene3 ""    1.16e+2
## 4 hygiene4 ""    1.41e-8
## 5 hygiene5 ""    7.80e+1
## 6 phg      ""    9.44e-9
```

```
phgmods <- cfa(phg, estimator = "MLR", data = pls2, std.lv = T, cluster = "country", group = "cgr", group.equal = c("loadings", "intercepts"), group.partial = c("hygiene4 ~ 1", "hygiene3 ~ 1"))
```

```
## Warning in lav_model_vcov(lavmodel = lavmodel, lavsamplestats = lavsamplestats, : lavaan WARNING:
##     The variance-covariance matrix of the estimated parameters (vcov)
##     does not appear to be positive definite! The smallest eigenvalue
##     (= 4.744489e-16) is close to zero. This may be a symptom that the
##     model is not identified.
```

```
summary(compareFit(phgmodc, phgmodw, phgmods, nested = T))
```

```
## ################### Nested Model Comparison #########################
## Scaled Chi-Squared Difference Test (method = "satorra.bentler.2001")
## 
## lavaan NOTE:
##     The "Chisq" column contains standard test statistics, not the
##     robust test that should be reported per model. A robust difference
##     test is a function of two standard (not robust) statistics.
##  
##         Df    AIC    BIC  Chisq Chisq diff Df diff Pr(>Chisq)    
## phgmodc 32 909993 911104  421.5                                  
## phgmodw 60 910613 911482 1098.1     95.045      28  3.171e-09 ***
## phgmods 74 911002 911749 1515.0     24.971      14    0.03486 *  
## ---
## Signif. codes:  0 '***' 0.001 '**' 0.01 '*' 0.05 '.' 0.1 ' ' 1
## 
## ####################### Model Fit Indices ###########################
##         chisq.scaled df.scaled pvalue.scaled rmsea.robust cfi.robust tli.robust
## phgmodc     157.734†        32          .000        .044†     0.996†      .990†
## phgmodw     231.312         60          .000        .050       .990       .987 
## phgmods     216.186         74          .000        .050       .988       .987 
##          srmr         aic         bic
## phgmodc .013† 909992.737† 911104.286†
## phgmodw .036  910613.326  911481.724 
## phgmods .041  911002.256  911749.079 
## 
## ################## Differences in Fit Indices #######################
##                   df.scaled rmsea.robust cfi.robust tli.robust  srmr     aic
## phgmodw - phgmodc        28        0.006     -0.006     -0.003 0.023 620.589
## phgmods - phgmodw        14        0.000     -0.002      0.000 0.005 388.930
##                       bic
## phgmodw - phgmodc 377.438
## phgmods - phgmodw 267.355
```

```
hygienes <- bind_rows(estimate_lavaan_ten_berge_per_group(phgmods))
```

### Policy

```
phs <- 'phs =~ psupport1 + psupport2 + psupport3 + psupport4 + psupport5'
phsmod <- cfa(phs, estimator = "MLR", data = pls2, std.lv = T, cluster = "country")
fitmeasures(phsmod, fit.measures = c("chisq.scaled", "df.scaled", "cfi.robust", "rmsea.ci.upper.robust", "srmr"))
```

```
##          chisq.scaled             df.scaled            cfi.robust 
##               234.924                 5.000                 0.970 
## rmsea.ci.upper.robust                  srmr 
##                 0.137                 0.026
```

```
miobj <- modindices(phsmod)
top_n(miobj, 3, mi)
```

```
##         lhs op       rhs       mi    epc sepc.lv sepc.all sepc.nox
## 1 psupport1 ~~ psupport2  947.762  0.631   0.631    0.270    0.270
## 2 psupport1 ~~ psupport5  931.350 -0.586  -0.586   -0.194   -0.194
## 3 psupport4 ~~ psupport5 2252.441  0.741   0.741    0.302    0.302
```

```
phs <- 'phs =~ psupport1 + psupport2 + psupport3 + psupport4 + psupport5
        psupport4 ~~ psupport5'
phsmod <- cfa(phs, estimator = "MLR", data = pls2, std.lv = T, cluster = "country")
fitmeasures(phsmod, fit.measures = c("chisq.scaled", "df.scaled", "cfi.robust", "rmsea.ci.upper.robust", "srmr"))
```

```
##          chisq.scaled             df.scaled            cfi.robust 
##               103.615                 4.000                 0.989 
## rmsea.ci.upper.robust                  srmr 
##                 0.098                 0.016
```

```
semPaths(phsmod, what = "std", edge.label.cex = 1.2, label.cex = 1.2)
```

```
reliability(phsmod)
```

```
##              phs
## alpha  0.8673143
## omega  0.8559081
## omega2 0.8559081
## omega3 0.8534035
## avevar 0.5679966
```

```
phsmodc <- cfa(phs, estimator = "MLR", data = pls2, std.lv = T, cluster = "country", group = "cgr")
```

```
## Warning in lav_model_vcov(lavmodel = lavmodel, lavsamplestats = lavsamplestats, : lavaan WARNING:
##     The variance-covariance matrix of the estimated parameters (vcov)
##     does not appear to be positive definite! The smallest eigenvalue
##     (= -1.159119e-15) is smaller than zero. This may be a symptom that
##     the model is not identified.
```

```
phsmodw <- cfa(phs, estimator = "MLR", data = pls2, std.lv = T, cluster = "country", group = "cgr", group.equal = "loadings")
```

```
## Warning in lav_model_vcov(lavmodel = lavmodel, lavsamplestats = lavsamplestats, : lavaan WARNING:
##     The variance-covariance matrix of the estimated parameters (vcov)
##     does not appear to be positive definite! The smallest eigenvalue
##     (= -1.626908e-16) is smaller than zero. This may be a symptom that
##     the model is not identified.
```

```
phsmods <- cfa(phs, estimator = "MLR", data = pls2, std.lv = T, cluster = "country", group = "cgr", group.equal = c("loadings", "intercepts"))
```

```
## Warning in lav_model_vcov(lavmodel = lavmodel, lavsamplestats = lavsamplestats, : lavaan WARNING:
##     The variance-covariance matrix of the estimated parameters (vcov)
##     does not appear to be positive definite! The smallest eigenvalue
##     (= 2.526425e-14) is close to zero. This may be a symptom that the
##     model is not identified.
```

```
summary(compareFit(phsmodc, phsmodw, phsmods, nested = T))
```

```
## ################### Nested Model Comparison #########################
## Scaled Chi-Squared Difference Test (method = "satorra.bentler.2001")
## 
## lavaan NOTE:
##     The "Chisq" column contains standard test statistics, not the
##     robust test that should be reported per model. A robust difference
##     test is a function of two standard (not robust) statistics.
##  
##         Df    AIC    BIC  Chisq Chisq diff Df diff Pr(>Chisq)    
## phsmodc 32 929997 931109 1269.8                                  
## phsmodw 60 931423 932291 2751.6     617.86      28  < 2.2e-16 ***
## phsmods 88 937153 937778 8537.6     146.31      28  < 2.2e-16 ***
## ---
## Signif. codes:  0 '***' 0.001 '**' 0.01 '*' 0.05 '.' 0.1 ' ' 1
## 
## ####################### Model Fit Indices ###########################
##         chisq.scaled df.scaled pvalue.scaled rmsea.robust cfi.robust tli.robust
## phsmodc     416.395†        32          .000        .082†      .989†      .972†
## phsmodw    1002.177         60          .000        .089       .976       .968 
## phsmods     590.640         88          .000        .123       .932       .938 
##          srmr         aic         bic
## phsmodc .016† 929997.148† 931108.698†
## phsmodw .055  931422.898  932291.296 
## phsmods .089  937152.950  937778.196 
## 
## ################## Differences in Fit Indices #######################
##                   df.scaled rmsea.robust cfi.robust tli.robust  srmr      aic
## phsmodw - phsmodc        28        0.007     -0.013     -0.005 0.038 1425.750
## phsmods - phsmodw        28        0.034     -0.044     -0.030 0.034 5730.052
##                        bic
## phsmodw - phsmodc 1182.599
## phsmods - phsmodw 5486.900
```

```
miobj <- modindices(phsmodw, free.remove = F)
miobj %>% filter(op == "=~") %>% group_by(lhs, rhs) %>% summarize(mi = mean(mi)) %>% top_n(3, mi)
```

```
## `summarise()` has grouped output by 'lhs'. You can override using the `.groups` argument.
```

```
## # A tibble: 3 x 3
## # Groups:   lhs [1]
##   lhs   rhs          mi
##   <chr> <chr>     <dbl>
## 1 phs   psupport3  40.5
## 2 phs   psupport4  33.0
## 3 phs   psupport5  48.9
```

```
phsmodw <- cfa(phs, estimator = "MLR", data = pls2, std.lv = T, cluster = "country", group = "cgr", group.equal = "loadings", group.partial = c("phs =~ psupport5"))
```

```
## Warning in lav_model_vcov(lavmodel = lavmodel, lavsamplestats = lavsamplestats, : lavaan WARNING:
##     The variance-covariance matrix of the estimated parameters (vcov)
##     does not appear to be positive definite! The smallest eigenvalue
##     (= -4.916157e-16) is smaller than zero. This may be a symptom that
##     the model is not identified.
```

```
phsmods <- cfa(phs, estimator = "MLR", data = pls2, std.lv = T, cluster = "country", group = "cgr", group.equal = c("loadings", "intercepts"), group.partial = c("phs =~ psupport5"))
```

```
## Warning in lav_model_vcov(lavmodel = lavmodel, lavsamplestats = lavsamplestats, : lavaan WARNING:
##     The variance-covariance matrix of the estimated parameters (vcov)
##     does not appear to be positive definite! The smallest eigenvalue
##     (= 1.100390e-14) is close to zero. This may be a symptom that the
##     model is not identified.
```

```
summary(compareFit(phsmodc, phsmodw, phsmods, nested = T))
```

```
## ################### Nested Model Comparison #########################
## Scaled Chi-Squared Difference Test (method = "satorra.bentler.2001")
## 
## lavaan NOTE:
##     The "Chisq" column contains standard test statistics, not the
##     robust test that should be reported per model. A robust difference
##     test is a function of two standard (not robust) statistics.
##  
##         Df    AIC    BIC  Chisq Chisq diff Df diff Pr(>Chisq)    
## phsmodc 32 929997 931109 1269.8                                  
## phsmodw 53 931020 931949 2334.4     199.44      21  < 2.2e-16 ***
## phsmods 81 936325 937011 7695.7     146.25      28  < 2.2e-16 ***
## ---
## Signif. codes:  0 '***' 0.001 '**' 0.01 '*' 0.05 '.' 0.1 ' ' 1
## 
## ####################### Model Fit Indices ###########################
##         chisq.scaled df.scaled pvalue.scaled rmsea.robust cfi.robust tli.robust
## phsmodc     416.395†        32          .000        .082†      .989†      .972†
## phsmodw     590.055         53          .000        .086       .980       .970 
## phsmods     504.273         81          .000        .121       .939       .940 
##          srmr         aic         bic
## phsmodc .016† 929997.148† 931108.698†
## phsmodw .046  931019.748  931948.934 
## phsmods .079  936325.002  937011.036 
## 
## ################## Differences in Fit Indices #######################
##                   df.scaled rmsea.robust cfi.robust tli.robust  srmr      aic
## phsmodw - phsmodc        21        0.004     -0.009     -0.003 0.029 1022.600
## phsmods - phsmodw        28        0.035     -0.041     -0.030 0.034 5305.253
##                        bic
## phsmodw - phsmodc  840.237
## phsmods - phsmodw 5062.102
```

```
miobj <- modindices(phsmodw, free.remove = F)
miobj %>% filter(op == "=~") %>% group_by(lhs, rhs) %>% summarize(mi = mean(mi)) %>% top_n(3, mi)
```

```
## `summarise()` has grouped output by 'lhs'. You can override using the `.groups` argument.
```

```
## # A tibble: 3 x 3
## # Groups:   lhs [1]
##   lhs   rhs          mi
##   <chr> <chr>     <dbl>
## 1 phs   psupport2  24.8
## 2 phs   psupport3  42.8
## 3 phs   psupport4  48.5
```

```
phsmodw <- cfa(phs, estimator = "MLR", data = pls2, std.lv = T, cluster = "country", group = "cgr", group.equal = "loadings", group.partial = c("phs =~ psupport5", "phs =~ psupport4"))
```

```
## Warning in lav_model_vcov(lavmodel = lavmodel, lavsamplestats = lavsamplestats, : lavaan WARNING:
##     The variance-covariance matrix of the estimated parameters (vcov)
##     does not appear to be positive definite! The smallest eigenvalue
##     (= -6.792731e-16) is smaller than zero. This may be a symptom that
##     the model is not identified.
```

```
phsmods <- cfa(phs, estimator = "MLR", data = pls2, std.lv = T, cluster = "country", group = "cgr", group.equal = c("loadings", "intercepts"), group.partial = c("phs =~ psupport5", "phs =~ psupport4"))
```

```
## Warning in lav_model_vcov(lavmodel = lavmodel, lavsamplestats = lavsamplestats, : lavaan WARNING:
##     The variance-covariance matrix of the estimated parameters (vcov)
##     does not appear to be positive definite! The smallest eigenvalue
##     (= 1.331279e-15) is close to zero. This may be a symptom that the
##     model is not identified.
```

```
summary(compareFit(phsmodc, phsmodw, phsmods, nested = T))
```

```
## ################### Nested Model Comparison #########################
## Scaled Chi-Squared Difference Test (method = "satorra.bentler.2001")
## 
## lavaan NOTE:
##     The "Chisq" column contains standard test statistics, not the
##     robust test that should be reported per model. A robust difference
##     test is a function of two standard (not robust) statistics.
##  
##         Df    AIC    BIC  Chisq Chisq diff Df diff Pr(>Chisq)    
## phsmodc 32 929997 931109 1269.8                                  
## phsmodw 46 930494 931484 1794.4     125.44      14  < 2.2e-16 ***
## phsmods 74 935100 935847 6457.1     133.66      28  9.967e-16 ***
## ---
## Signif. codes:  0 '***' 0.001 '**' 0.01 '*' 0.05 '.' 0.1 ' ' 1
## 
## ####################### Model Fit Indices ###########################
##         chisq.scaled df.scaled pvalue.scaled rmsea.robust cfi.robust tli.robust
## phsmodc     416.395†        32          .000        .082       .989†      .972 
## phsmodw     528.677         46          .000        .081†      .985       .973†
## phsmods     421.770         74          .000        .115       .950       .946 
##          srmr         aic         bic
## phsmodc .016† 929997.148† 931108.698†
## phsmodw .033  930493.709  931483.683 
## phsmods .070  935100.406  935847.229 
## 
## ################## Differences in Fit Indices #######################
##                   df.scaled rmsea.robust cfi.robust tli.robust  srmr      aic
## phsmodw - phsmodc        14       -0.001     -0.004      0.001 0.017  496.561
## phsmods - phsmodw        28        0.034     -0.035     -0.027 0.037 4606.698
##                        bic
## phsmodw - phsmodc  374.985
## phsmods - phsmodw 4363.546
```

```
miobj <- modindices(phsmods, free.remove = F)
miobj %>% filter(op == "~1") %>% group_by(lhs, rhs) %>% summarize(mi = mean(mi)) %>% top_n(3, mi)
```

```
## `summarise()` has grouped output by 'lhs'. You can override using the `.groups` argument.
```

```
## # A tibble: 6 x 3
## # Groups:   lhs [6]
##   lhs       rhs      mi
##   <chr>     <chr> <dbl>
## 1 phs       ""     34.8
## 2 psupport1 ""     33.4
## 3 psupport2 ""    108. 
## 4 psupport3 ""    402. 
## 5 psupport4 ""     24.9
## 6 psupport5 ""     20.0
```

```
phsmods <- cfa(phs, estimator = "MLR", data = pls2, std.lv = T, cluster = "country", group = "cgr", group.equal = c("loadings", "intercepts"), group.partial = c("phs =~ psupport5", "phs =~ psupport4", "psupport3 ~ 1"))
```

```
## Warning in lav_model_vcov(lavmodel = lavmodel, lavsamplestats = lavsamplestats, : lavaan WARNING:
##     The variance-covariance matrix of the estimated parameters (vcov)
##     does not appear to be positive definite! The smallest eigenvalue
##     (= 4.473004e-16) is close to zero. This may be a symptom that the
##     model is not identified.
```

```
summary(compareFit(phsmodc, phsmodw, phsmods, nested = T))
```

```
## ################### Nested Model Comparison #########################
## Scaled Chi-Squared Difference Test (method = "satorra.bentler.2001")
## 
## lavaan NOTE:
##     The "Chisq" column contains standard test statistics, not the
##     robust test that should be reported per model. A robust difference
##     test is a function of two standard (not robust) statistics.
##  
##         Df    AIC    BIC  Chisq Chisq diff Df diff Pr(>Chisq)    
## phsmodc 32 929997 931109 1269.8                                  
## phsmodw 46 930494 931484 1794.4    125.445      14    < 2e-16 ***
## phsmods 67 931385 932193 2728.0     31.342      21    0.06816 .  
## ---
## Signif. codes:  0 '***' 0.001 '**' 0.01 '*' 0.05 '.' 0.1 ' ' 1
## 
## ####################### Model Fit Indices ###########################
##         chisq.scaled df.scaled pvalue.scaled rmsea.robust cfi.robust tli.robust
## phsmodc     416.395         32          .000        .082       .989†      .972 
## phsmodw     528.677         46          .000        .081       .985       .973 
## phsmods     233.826†        67          .000        .073†      .982       .978†
##          srmr         aic         bic
## phsmodc .016† 929997.148† 931108.698†
## phsmodw .033  930493.709  931483.683 
## phsmods .043  931385.299  932192.909 
## 
## ################## Differences in Fit Indices #######################
##                   df.scaled rmsea.robust cfi.robust tli.robust  srmr     aic
## phsmodw - phsmodc        14       -0.001     -0.004      0.001 0.017 496.561
## phsmods - phsmodw        21       -0.008     -0.003      0.005 0.009 891.590
##                       bic
## phsmodw - phsmodc 374.985
## phsmods - phsmodw 709.226
```

```
policys <- bind_rows(estimate_lavaan_ten_berge_per_group(phsmods))
```

### Collective narcissism & conspiracy theories

```
cns <- 'cons =~ ctheory2 + ctheory1 + ctheory3 + ctheory4
        coln =~ cnarc1 + cnarc2 + cnarc3'
cnsmod <- cfa(cns, estimator = "MLR", data = pls2, std.lv = T, cluster = "country")
fitmeasures(cnsmod, fit.measures = c("chisq.scaled", "df.scaled", "cfi.robust", "rmsea.ci.upper.robust", "srmr"))
```

```
##          chisq.scaled             df.scaled            cfi.robust 
##               402.839                13.000                    NA 
## rmsea.ci.upper.robust                  srmr 
##                 0.069                 0.021
```

```
semPaths(cnsmod, what = "std", edge.label.cex = 1.2, label.cex = 1.2)
```

```
reliability(cnsmod)
```

```
##             cons      coln
## alpha  0.9146516 0.8665065
## omega  0.9150845 0.8682775
## omega2 0.9150845 0.8682775
## omega3 0.9157210 0.8682536
## avevar 0.7293901 0.6881130
```

```
cnsmodc <- cfa(cns, estimator = "MLR", data = pls2, std.lv = T, cluster = "country", group = "cgr")
```

```
## Warning in lav_model_vcov(lavmodel = lavmodel, lavsamplestats = lavsamplestats, : lavaan WARNING:
##     The variance-covariance matrix of the estimated parameters (vcov)
##     does not appear to be positive definite! The smallest eigenvalue
##     (= -2.938637e-15) is smaller than zero. This may be a symptom that
##     the model is not identified.
```

```
cnsmodw <- cfa(cns, estimator = "MLR", data = pls2, std.lv = T, cluster = "country", group = "cgr", group.equal = "loadings")
```

```
## Warning in lav_model_vcov(lavmodel = lavmodel, lavsamplestats = lavsamplestats, : lavaan WARNING:
##     The variance-covariance matrix of the estimated parameters (vcov)
##     does not appear to be positive definite! The smallest eigenvalue
##     (= -9.821987e-16) is smaller than zero. This may be a symptom that
##     the model is not identified.
```

```
cnsmods <- cfa(cns, estimator = "MLR", data = pls2, std.lv = T, cluster = "country", group = "cgr", group.equal = c("loadings", "intercepts"))
```

```
## Warning in lav_model_vcov(lavmodel = lavmodel, lavsamplestats = lavsamplestats, : lavaan WARNING:
##     The variance-covariance matrix of the estimated parameters (vcov)
##     does not appear to be positive definite! The smallest eigenvalue
##     (= 5.351242e-15) is close to zero. This may be a symptom that the
##     model is not identified.
```

```
summary(compareFit(cnsmodc, cnsmodw, cnsmods, nested = T))
```

```
## ################### Nested Model Comparison #########################
## Scaled Chi-Squared Difference Test (method = "satorra.bentler.2001")
## 
## lavaan NOTE:
##     The "Chisq" column contains standard test statistics, not the
##     robust test that should be reported per model. A robust difference
##     test is a function of two standard (not robust) statistics.
##  
##          Df     AIC     BIC  Chisq Chisq diff Df diff Pr(>Chisq)    
## cnsmodc 104 1365940 1367468 3104.7                                  
## cnsmodw 139 1366674 1367898 3908.8     93.754      35  2.892e-07 ***
## cnsmods 174 1368885 1369806 6190.0    131.892      35  3.683e-13 ***
## ---
## Signif. codes:  0 '***' 0.001 '**' 0.01 '*' 0.05 '.' 0.1 ' ' 1
## 
## ####################### Model Fit Indices ###########################
##         chisq.scaled df.scaled pvalue.scaled rmsea.scaled cfi.scaled tli.scaled
## cnsmodc    1642.528        104          .000        .052         NA         NA 
## cnsmodw    1093.732        139          .000        .035         NA         NA 
## cnsmods     977.269†       174          .000        .029†        NA         NA 
##          srmr          aic          bic
## cnsmodc .023† 1365939.888† 1367468.269†
## cnsmodw .034  1366673.980  1367898.421 
## cnsmods .040  1368885.110  1369805.612 
## 
## ################## Differences in Fit Indices #######################
##                   df.scaled rmsea.scaled cfi.scaled tli.scaled  srmr      aic
## cnsmodw - cnsmodc        35       -0.017         NA         NA 0.011  734.092
## cnsmods - cnsmodw        35       -0.006         NA         NA 0.006 2211.130
##                        bic
## cnsmodw - cnsmodc  430.152
## cnsmods - cnsmodw 1907.191
```

```
miobj <- modindices(cnsmods, free.remove = F)
miobj %>% filter(op == "=~") %>% group_by(lhs, rhs) %>% summarize(mi = mean(mi)) %>% top_n(3, mi)
```

```
## `summarise()` has grouped output by 'lhs'. You can override using the `.groups` argument.
```

```
## # A tibble: 6 x 3
## # Groups:   lhs [2]
##   lhs   rhs         mi
##   <chr> <chr>    <dbl>
## 1 coln  cnarc2    31.2
## 2 coln  cnarc3    29.2
## 3 coln  ctheory1  82.8
## 4 cons  cnarc3    34.8
## 5 cons  ctheory1  45.6
## 6 cons  ctheory3  31.1
```

```
cnsmodw <- cfa(cns, estimator = "MLR", data = pls2, std.lv = T, cluster = "country", group = "cgr", group.equal = "loadings", group.partial = c("cons =~ ctheory1"))
```

```
## Warning in lav_model_vcov(lavmodel = lavmodel, lavsamplestats = lavsamplestats, : lavaan WARNING:
##     The variance-covariance matrix of the estimated parameters (vcov)
##     does not appear to be positive definite! The smallest eigenvalue
##     (= -6.781017e-16) is smaller than zero. This may be a symptom that
##     the model is not identified.
```

```
cnsmods <- cfa(cns, estimator = "MLR", data = pls2, std.lv = T, cluster = "country", group = "cgr", group.equal = c("loadings", "intercepts"), group.partial = c("cons =~ ctheory1"))
```

```
## Warning in lav_model_vcov(lavmodel = lavmodel, lavsamplestats = lavsamplestats, : lavaan WARNING:
##     The variance-covariance matrix of the estimated parameters (vcov)
##     does not appear to be positive definite! The smallest eigenvalue
##     (= 2.558903e-15) is close to zero. This may be a symptom that the
##     model is not identified.
```

```
summary(compareFit(cnsmodc, cnsmodw, cnsmods, nested = T))
```

```
## ################### Nested Model Comparison #########################
## Scaled Chi-Squared Difference Test (method = "satorra.bentler.2001")
## 
## lavaan NOTE:
##     The "Chisq" column contains standard test statistics, not the
##     robust test that should be reported per model. A robust difference
##     test is a function of two standard (not robust) statistics.
##  
##          Df     AIC     BIC  Chisq Chisq diff Df diff Pr(>Chisq)    
## cnsmodc 104 1365940 1367468 3104.7                                  
## cnsmodw 132 1366555 1367840 3775.7     91.385      28  1.203e-08 ***
## cnsmods 167 1368504 1369485 5794.9    129.617      35  8.669e-13 ***
## ---
## Signif. codes:  0 '***' 0.001 '**' 0.01 '*' 0.05 '.' 0.1 ' ' 1
## 
## ####################### Model Fit Indices ###########################
##         chisq.scaled df.scaled pvalue.scaled rmsea.scaled cfi.scaled tli.scaled
## cnsmodc    1642.528        104          .000        .052         NA         NA 
## cnsmodw    1239.281        132          .000        .039         NA         NA 
## cnsmods    1021.482†       167          .000        .031†        NA         NA 
##          srmr          aic          bic
## cnsmodc .023† 1365939.888† 1367468.269†
## cnsmodw .032  1366554.847  1367840.076 
## cnsmods .038  1368504.027  1369485.317 
## 
## ################## Differences in Fit Indices #######################
##                   df.scaled rmsea.scaled cfi.scaled tli.scaled  srmr      aic
## cnsmodw - cnsmodc        28       -0.013         NA         NA 0.009  614.958
## cnsmods - cnsmodw        35       -0.009         NA         NA 0.006 1949.181
##                        bic
## cnsmodw - cnsmodc  371.807
## cnsmods - cnsmodw 1645.241
```

```
cnar <- bind_rows(estimate_lavaan_ten_berge_per_group(cnsmods))
collectivenar <- cnar$scores[2]
conspiracy <- cnar$scores[1]
```

### Open-mindedness

```
pls2$omind1 <- 10-pls2$omind1
pls2$omind5 <- 10-pls2$omind5
pls2$omind6 <- 10-pls2$omind6

om <- 'om =~ omind1 + omind2 + omind3 + omind4 + omind5 + omind6'
ommod <- cfa(om, estimator = "MLR", data = pls2, std.lv = T, cluster = "country")
fitmeasures(ommod, fit.measures = c("chisq.scaled", "df.scaled", "cfi.robust", "rmsea.ci.upper.robust", "srmr"))
```

```
##          chisq.scaled             df.scaled            cfi.robust 
##              3586.669                 9.000                 0.784 
## rmsea.ci.upper.robust                  srmr 
##                 0.170                 0.086
```

```
miobj <- modindices(ommod)
top_n(miobj, 3, mi)
```

```
##      lhs op    rhs       mi   epc sepc.lv sepc.all sepc.nox
## 1 omind1 ~~ omind5 2189.200 1.573   1.573    0.234    0.234
## 2 omind1 ~~ omind6 4506.275 2.355   2.355    0.334    0.334
## 3 omind5 ~~ omind6 4519.562 2.077   2.077    0.343    0.343
```

```
om <- 'om1 =~ omind2 + omind3 + omind4 + omind1 + omind5 + omind6
       omind5 ~~ omind6'
ommod <- cfa(om, estimator = "MLR", data = pls2, std.lv = T, cluster = "country")
fitmeasures(ommod, fit.measures = c("chisq.scaled", "df.scaled", "cfi.robust", "rmsea.ci.upper.robust", "srmr"))
```

```
##          chisq.scaled             df.scaled            cfi.robust 
##              2124.798                 8.000                 0.876 
## rmsea.ci.upper.robust                  srmr 
##                 0.138                 0.072
```

```
miobj <- modindices(ommod)
top_n(miobj, 3, mi)
```

```
##      lhs op    rhs       mi    epc sepc.lv sepc.all sepc.nox
## 1 omind3 ~~ omind1  402.334 -0.444  -0.444   -0.124   -0.124
## 2 omind1 ~~ omind5  808.758  0.908   0.908    0.131    0.131
## 3 omind1 ~~ omind6 3119.027  1.861   1.861    0.257    0.257
```

```
om <- 'om1 =~ omind2 + omind3 + omind4 + omind1 + omind5 + omind6
       omind5 ~~ omind6
       omind1 ~~ omind6'
ommod <- cfa(om, estimator = "MLR", data = pls2, std.lv = T, cluster = "country")
fitmeasures(ommod, fit.measures = c("chisq.scaled", "df.scaled", "cfi.robust", "rmsea.ci.upper.robust", "srmr"))
```

```
##          chisq.scaled             df.scaled            cfi.robust 
##               965.299                 7.000                 0.944 
## rmsea.ci.upper.robust                  srmr 
##                 0.101                 0.047
```

```
miobj <- modindices(ommod)
top_n(miobj, 3, mi)
```

```
##      lhs op    rhs       mi    epc sepc.lv sepc.all sepc.nox
## 1 omind3 ~~ omind4   74.385  0.265   0.265    0.171    0.171
## 2 omind3 ~~ omind1  165.828 -0.271  -0.271   -0.076   -0.076
## 3 omind1 ~~ omind5 2667.445  1.772   1.772    0.255    0.255
```

```
om <- 'om1 =~ omind2 + omind3 + omind4 + omind1 + omind5 + omind6
       omind5 ~~ omind6
       omind1 ~~ omind6
       omind1 ~~ omind5'
ommod <- cfa(om, estimator = "MLR", data = pls2, std.lv = T, cluster = "country")
fitmeasures(ommod, fit.measures = c("chisq.scaled", "df.scaled", "cfi.robust", "rmsea.ci.upper.robust", "srmr"))
```

```
##          chisq.scaled             df.scaled            cfi.robust 
##                32.460                 6.000                 0.998 
## rmsea.ci.upper.robust                  srmr 
##                 0.025                 0.007
```

```
semPaths(ommod, what = "std", edge.label.cex = 1.2, label.cex = 1.2)
```

```
reliability(ommod)
```

```
##              om1
## alpha  0.6821732
## omega  0.4951914
## omega2 0.4951914
## omega3 0.4953697
## avevar 0.2108656
```

```
ommodc <- cfa(om, estimator = "MLR", data = pls2, std.lv = T, cluster = "country", group = "cgr")
```

```
## Warning in lav_model_vcov(lavmodel = lavmodel, lavsamplestats = lavsamplestats, : lavaan WARNING:
##     The variance-covariance matrix of the estimated parameters (vcov)
##     does not appear to be positive definite! The smallest eigenvalue
##     (= -2.769915e-15) is smaller than zero. This may be a symptom that
##     the model is not identified.
```

```
ommodw <- cfa(om, estimator = "MLR", data = pls2, std.lv = T, cluster = "country", group = "cgr", group.equal = "loadings")
```

```
## Warning in lav_model_vcov(lavmodel = lavmodel, lavsamplestats = lavsamplestats, : lavaan WARNING:
##     The variance-covariance matrix of the estimated parameters (vcov)
##     does not appear to be positive definite! The smallest eigenvalue
##     (= -8.374138e-16) is smaller than zero. This may be a symptom that
##     the model is not identified.
```

```
ommods <- cfa(om, estimator = "MLR", data = pls2, std.lv = T, cluster = "country", group = "cgr", group.equal = c("loadings", "intercepts"))
```

```
## Warning in lav_model_vcov(lavmodel = lavmodel, lavsamplestats = lavsamplestats, : lavaan WARNING:
##     The variance-covariance matrix of the estimated parameters (vcov)
##     does not appear to be positive definite! The smallest eigenvalue
##     (= 1.807072e-15) is close to zero. This may be a symptom that the
##     model is not identified.
```

```
summary(compareFit(ommodc, ommodw, ommods, nested = T))
```

```
## ################### Nested Model Comparison #########################
## Scaled Chi-Squared Difference Test (method = "satorra.bentler.2001")
## 
## lavaan NOTE:
##     The "Chisq" column contains standard test statistics, not the
##     robust test that should be reported per model. A robust difference
##     test is a function of two standard (not robust) statistics.
##  
##         Df     AIC     BIC   Chisq Chisq diff Df diff Pr(>Chisq)    
## ommodc  48 1119124 1120583  233.92                                  
## ommodw  83 1119285 1120440  465.48     68.695      35  0.0005733 ***
## ommods 118 1120970 1121821 2219.71     77.135      35   5.25e-05 ***
## ---
## Signif. codes:  0 '***' 0.001 '**' 0.01 '*' 0.05 '.' 0.1 ' ' 1
## 
## ####################### Model Fit Indices ###########################
##        chisq.scaled df.scaled pvalue.scaled rmsea.robust cfi.robust tli.robust
## ommodc     142.246†        48          .000        .024†     0.997†      .992†
## ommodw     196.202         83          .000        .024       .995       .992 
## ommods     263.800        118          .000        .044       .976       .976 
##         srmr          aic          bic
## ommodc .010† 1119123.723† 1120582.631 
## ommodw .022  1119285.281  1120440.250†
## ommods .043  1120969.516  1121820.546 
## 
## ################## Differences in Fit Indices #######################
##                 df.scaled rmsea.robust cfi.robust tli.robust  srmr      aic
## ommodw - ommodc        35        0.000     -0.002      0.000 0.011  161.558
## ommods - ommodw        35        0.019     -0.019     -0.017 0.022 1684.235
##                      bic
## ommodw - ommodc -142.381
## ommods - ommodw 1380.296
```

```
miobj <- modindices(ommods, free.remove = F)
miobj %>% filter(op == "~1") %>% group_by(lhs, rhs) %>% summarize(mi = mean(mi)) %>% top_n(3, mi)
```

```
## `summarise()` has grouped output by 'lhs'. You can override using the `.groups` argument.
```

```
## # A tibble: 7 x 3
## # Groups:   lhs [7]
##   lhs    rhs         mi
##   <chr>  <chr>    <dbl>
## 1 om1    ""    5.89e-10
## 2 omind1 ""    5.77e+ 1
## 3 omind2 ""    1.92e+ 1
## 4 omind3 ""    2.26e+ 1
## 5 omind4 ""    3.46e+ 1
## 6 omind5 ""    4.56e+ 1
## 7 omind6 ""    1.97e+ 1
```

```
ommods <- cfa(om, estimator = "MLR", data = pls2, std.lv = T, cluster = "country", group = "cgr", group.equal = c("loadings", "intercepts"), group.partial = "omind1 ~ 1")
```

```
## Warning in lav_model_vcov(lavmodel = lavmodel, lavsamplestats = lavsamplestats, : lavaan WARNING:
##     The variance-covariance matrix of the estimated parameters (vcov)
##     does not appear to be positive definite! The smallest eigenvalue
##     (= 7.088118e-16) is close to zero. This may be a symptom that the
##     model is not identified.
```

```
summary(compareFit(ommodc, ommodw, ommods, nested = T))
```

```
## ################### Nested Model Comparison #########################
## Scaled Chi-Squared Difference Test (method = "satorra.bentler.2001")
## 
## lavaan NOTE:
##     The "Chisq" column contains standard test statistics, not the
##     robust test that should be reported per model. A robust difference
##     test is a function of two standard (not robust) statistics.
##  
##         Df     AIC     BIC   Chisq Chisq diff Df diff Pr(>Chisq)    
## ommodc  48 1119124 1120583  233.92                                  
## ommodw  83 1119285 1120440  465.48     68.695      35  0.0005733 ***
## ommods 111 1120503 1121415 1739.02     69.491      28  2.191e-05 ***
## ---
## Signif. codes:  0 '***' 0.001 '**' 0.01 '*' 0.05 '.' 0.1 ' ' 1
## 
## ####################### Model Fit Indices ###########################
##        chisq.scaled df.scaled pvalue.scaled rmsea.robust cfi.robust tli.robust
## ommodc     142.246†        48          .000        .024†     0.997†      .992†
## ommodw     196.202         83          .000        .024       .995       .992 
## ommods     271.853        111          .000        .041       .980       .978 
##         srmr          aic          bic
## ommodc .010† 1119123.723† 1120582.631 
## ommodw .022  1119285.281  1120440.250†
## ommods .038  1120502.823  1121414.641 
## 
## ################## Differences in Fit Indices #######################
##                 df.scaled rmsea.robust cfi.robust tli.robust  srmr      aic
## ommodw - ommodc        35        0.000     -0.002      0.000 0.011  161.558
## ommods - ommodw        28        0.017     -0.015     -0.014 0.016 1217.542
##                      bic
## ommodw - ommodc -142.381
## ommods - ommodw  974.391
```

```
miobj <- modindices(ommods, free.remove = F)
miobj %>% filter(op == "~1") %>% group_by(lhs, rhs) %>% summarize(mi = mean(mi)) %>% top_n(3, mi)
```

```
## `summarise()` has grouped output by 'lhs'. You can override using the `.groups` argument.
```

```
## # A tibble: 7 x 3
## # Groups:   lhs [7]
##   lhs    rhs         mi
##   <chr>  <chr>    <dbl>
## 1 om1    ""    5.87e-10
## 2 omind1 ""    5.60e-10
## 3 omind2 ""    1.94e+ 1
## 4 omind3 ""    2.19e+ 1
## 5 omind4 ""    3.47e+ 1
## 6 omind5 ""    5.78e+ 1
## 7 omind6 ""    1.78e+ 1
```

```
ommods <- cfa(om, estimator = "MLR", data = pls2, std.lv = T, cluster = "country", group = "cgr", group.equal = c("loadings", "intercepts"), group.partial = c("omind1 ~ 1", "omind5 ~ 1"))
```

```
## Warning in lav_model_vcov(lavmodel = lavmodel, lavsamplestats = lavsamplestats, : lavaan WARNING:
##     The variance-covariance matrix of the estimated parameters (vcov)
##     does not appear to be positive definite! The smallest eigenvalue
##     (= 5.940965e-16) is close to zero. This may be a symptom that the
##     model is not identified.
```

```
summary(compareFit(ommodc, ommodw, ommods, nested = T))
```

```
## ################### Nested Model Comparison #########################
## Scaled Chi-Squared Difference Test (method = "satorra.bentler.2001")
## 
## lavaan NOTE:
##     The "Chisq" column contains standard test statistics, not the
##     robust test that should be reported per model. A robust difference
##     test is a function of two standard (not robust) statistics.
##  
##         Df     AIC     BIC   Chisq Chisq diff Df diff Pr(>Chisq)    
## ommodc  48 1119124 1120583  233.92                                  
## ommodw  83 1119285 1120440  465.48     68.695      35  0.0005733 ***
## ommods 104 1120026 1120998 1247.72     43.467      21  0.0027395 ** 
## ---
## Signif. codes:  0 '***' 0.001 '**' 0.01 '*' 0.05 '.' 0.1 ' ' 1
## 
## ####################### Model Fit Indices ###########################
##        chisq.scaled df.scaled pvalue.scaled rmsea.robust cfi.robust tli.robust
## ommodc     142.246†        48          .000        .024†     0.997†      .992†
## ommodw     196.202         83          .000        .024       .995       .992 
## ommods     225.738        104          .000        .034       .987       .985 
##         srmr          aic          bic
## ommodc .010† 1119123.723† 1120582.631 
## ommodw .022  1119285.281  1120440.250†
## ommods .032  1120025.530  1120998.136 
## 
## ################## Differences in Fit Indices #######################
##                 df.scaled rmsea.robust cfi.robust tli.robust  srmr     aic
## ommodw - ommodc        35         0.00     -0.002      0.000 0.011 161.558
## ommods - ommodw        21         0.01     -0.008     -0.008 0.010 740.249
##                      bic
## ommodw - ommodc -142.381
## ommods - ommodw  557.886
```

```
ominds <- bind_rows(estimate_lavaan_ten_berge_per_group(ommods))
```

### Morality as cooperation

```
mcw <- 'mcoop =~ mcoop1 + mcoop2 + mcoop3 + mcoop4 + mcoop5 + mcoop6 + mcoop7'
mcwmod <- cfa(mcw, estimator = "MLR", data = pls2, std.lv = T, cluster = "country")
fitmeasures(mcwmod, fit.measures = c("chisq.scaled", "df.scaled", "cfi.robust", "rmsea.ci.upper.robust", "srmr"))
```

```
##          chisq.scaled             df.scaled            cfi.robust 
##             12600.405                14.000                 0.676 
## rmsea.ci.upper.robust                  srmr 
##                 0.214                 0.113
```

```
mcw <- 'mcoop =~ mcoop1 + mcoop2 + mcoop3 + mcoop4 + mcoop5'
mcwmod <- cfa(mcw, estimator = "MLR", data = pls2, std.lv = T, cluster = "country")
fitmeasures(mcwmod, fit.measures = c("chisq.scaled", "df.scaled", "cfi.robust", "rmsea.ci.upper.robust", "srmr"))
```

```
##          chisq.scaled             df.scaled            cfi.robust 
##               323.237                 5.000                 0.956 
## rmsea.ci.upper.robust                  srmr 
##                 0.117                 0.028
```

```
miobj <- modindices(mcwmod)
top_n(miobj, 3, mi)
```

```
##      lhs op    rhs       mi    epc sepc.lv sepc.all sepc.nox
## 1 mcoop1 ~~ mcoop2 2051.375  1.093   1.093    0.331    0.331
## 2 mcoop1 ~~ mcoop4 1468.696 -0.869  -0.869   -0.303   -0.303
## 3 mcoop3 ~~ mcoop4 1743.919  0.875   0.875    0.350    0.350
```

```
mcw <- 'mcoop =~ mcoop1 + mcoop2 + mcoop3 + mcoop4 + mcoop5
        mcoop1 ~~ mcoop2'

mcwmod <- cfa(mcw, estimator = "MLR", data = pls2, std.lv = T, cluster = "country")
fitmeasures(mcwmod, fit.measures = c("chisq.scaled", "df.scaled", "cfi.robust", "rmsea.ci.upper.robust", "srmr"))
```

```
##          chisq.scaled             df.scaled            cfi.robust 
##                81.609                 4.000                 0.991 
## rmsea.ci.upper.robust                  srmr 
##                 0.066                 0.014
```

```
reliability(mcwmod)
```

```
##            mcoop
## alpha  0.7638321
## omega  0.7317088
## omega2 0.7317088
## omega3 0.7288815
## avevar 0.3837902
```

```
semPaths(mcwmod, what = "std", edge.label.cex = 1.2, label.cex = 1.2)
```

```
mcwmodc <- cfa(mcw, estimator = "MLR", data = pls2, std.lv = T, cluster = "country", group = "cgr")
```

```
## Warning in lav_model_vcov(lavmodel = lavmodel, lavsamplestats = lavsamplestats, : lavaan WARNING:
##     The variance-covariance matrix of the estimated parameters (vcov)
##     does not appear to be positive definite! The smallest eigenvalue
##     (= -3.253700e-16) is smaller than zero. This may be a symptom that
##     the model is not identified.
```

```
mcwmodw <- cfa(mcw, estimator = "MLR", data = pls2, std.lv = T, cluster = "country", group = "cgr", group.equal = "loadings")
```

```
## Warning in lav_model_vcov(lavmodel = lavmodel, lavsamplestats = lavsamplestats, : lavaan WARNING:
##     The variance-covariance matrix of the estimated parameters (vcov)
##     does not appear to be positive definite! The smallest eigenvalue
##     (= 4.336372e-17) is close to zero. This may be a symptom that the
##     model is not identified.
```

```
mcwmods <- cfa(mcw, estimator = "MLR", data = pls2, std.lv = T, cluster = "country", group = "cgr", group.equal = c("loadings", "intercepts"))
```

```
## Warning in lav_model_vcov(lavmodel = lavmodel, lavsamplestats = lavsamplestats, : lavaan WARNING:
##     The variance-covariance matrix of the estimated parameters (vcov)
##     does not appear to be positive definite! The smallest eigenvalue
##     (= 5.791229e-15) is close to zero. This may be a symptom that the
##     model is not identified.
```

```
summary(compareFit(mcwmodc, mcwmodw, mcwmods, nested = T))
```

```
## ################### Nested Model Comparison #########################
## Scaled Chi-Squared Difference Test (method = "satorra.bentler.2001")
## 
## lavaan NOTE:
##     The "Chisq" column contains standard test statistics, not the
##     robust test that should be reported per model. A robust difference
##     test is a function of two standard (not robust) statistics.
##  
##         Df    AIC    BIC  Chisq Chisq diff Df diff Pr(>Chisq)    
## mcwmodc 32 944927 946038  717.7                                  
## mcwmodw 60 945283 946152 1130.1    126.177      28  2.014e-14 ***
## mcwmods 88 947890 948515 3793.1     74.695      28  4.006e-06 ***
## ---
## Signif. codes:  0 '***' 0.001 '**' 0.01 '*' 0.05 '.' 0.1 ' ' 1
## 
## ####################### Model Fit Indices ###########################
##         chisq.scaled df.scaled pvalue.scaled rmsea.robust cfi.robust tli.robust
## mcwmodc     157.232†        32          .000        .057       .990†      .975 
## mcwmodw     285.400         60          .000        .052†      .984       .979†
## mcwmods     270.097         88          .000        .073       .955       .959 
##          srmr         aic         bic
## mcwmodc .016† 944926.748† 946038.297†
## mcwmodw .031  945283.155  946151.554 
## mcwmods .052  947890.100  948515.346 
## 
## ################## Differences in Fit Indices #######################
##                   df.scaled rmsea.robust cfi.robust tli.robust  srmr      aic
## mcwmodw - mcwmodc        28       -0.005     -0.006      0.004 0.016  356.408
## mcwmods - mcwmodw        28        0.021     -0.029     -0.020 0.021 2606.944
##                        bic
## mcwmodw - mcwmodc  113.256
## mcwmods - mcwmodw 2363.793
```

```
miobj <- modindices(mcwmods, free.remove = F)
miobj %>% filter(op == "~1") %>% group_by(lhs, rhs) %>% summarize(mi = mean(mi)) %>% top_n(3, mi)
```

```
## `summarise()` has grouped output by 'lhs'. You can override using the `.groups` argument.
```

```
## # A tibble: 6 x 3
## # Groups:   lhs [6]
##   lhs    rhs        mi
##   <chr>  <chr>   <dbl>
## 1 mcoop  ""    1.38e-8
## 2 mcoop1 ""    3.29e+1
## 3 mcoop2 ""    1.43e+2
## 4 mcoop3 ""    1.52e+2
## 5 mcoop4 ""    1.96e+1
## 6 mcoop5 ""    3.84e+1
```

```
mcwmods <- cfa(mcw, estimator = "MLR", data = pls2, std.lv = T, cluster = "country", group = "cgr", group.equal = c("loadings", "intercepts"), group.partial = c("mcoop3 ~ 1"))
```

```
## Warning in lav_model_vcov(lavmodel = lavmodel, lavsamplestats = lavsamplestats, : lavaan WARNING:
##     The variance-covariance matrix of the estimated parameters (vcov)
##     does not appear to be positive definite! The smallest eigenvalue
##     (= 4.257793e-15) is close to zero. This may be a symptom that the
##     model is not identified.
```

```
summary(compareFit(ommodc, ommodw, ommods, nested = T))
```

```
## ################### Nested Model Comparison #########################
## Scaled Chi-Squared Difference Test (method = "satorra.bentler.2001")
## 
## lavaan NOTE:
##     The "Chisq" column contains standard test statistics, not the
##     robust test that should be reported per model. A robust difference
##     test is a function of two standard (not robust) statistics.
##  
##         Df     AIC     BIC   Chisq Chisq diff Df diff Pr(>Chisq)    
## ommodc  48 1119124 1120583  233.92                                  
## ommodw  83 1119285 1120440  465.48     68.695      35  0.0005733 ***
## ommods 104 1120026 1120998 1247.72     43.467      21  0.0027395 ** 
## ---
## Signif. codes:  0 '***' 0.001 '**' 0.01 '*' 0.05 '.' 0.1 ' ' 1
## 
## ####################### Model Fit Indices ###########################
##        chisq.scaled df.scaled pvalue.scaled rmsea.robust cfi.robust tli.robust
## ommodc     142.246†        48          .000        .024†     0.997†      .992†
## ommodw     196.202         83          .000        .024       .995       .992 
## ommods     225.738        104          .000        .034       .987       .985 
##         srmr          aic          bic
## ommodc .010† 1119123.723† 1120582.631 
## ommodw .022  1119285.281  1120440.250†
## ommods .032  1120025.530  1120998.136 
## 
## ################## Differences in Fit Indices #######################
##                 df.scaled rmsea.robust cfi.robust tli.robust  srmr     aic
## ommodw - ommodc        35         0.00     -0.002      0.000 0.011 161.558
## ommods - ommodw        21         0.01     -0.008     -0.008 0.010 740.249
##                      bic
## ommodw - ommodc -142.381
## ommods - ommodw  557.886
```

```
mcoops <- bind_rows(estimate_lavaan_ten_berge_per_group(mcwmods))
```

### Belonging

```
sb <- 'sb =~ sbelong1 + sbelong2 + sbelong3 + sbelong4'
sbmod <- cfa(sb, estimator = "MLR", data = pls2, std.lv = T, cluster = "country")
fitmeasures(sbmod, fit.measures = c("chisq.scaled", "df.scaled", "cfi.robust", "rmsea.ci.upper.robust", "srmr"))
```

```
##          chisq.scaled             df.scaled            cfi.robust 
##               198.459                 2.000                 0.988 
## rmsea.ci.upper.robust                  srmr 
##                 0.115                 0.016
```

```
miobj <- modindices(sbmod)
top_n(miobj, 3, mi)
```

```
##        lhs op      rhs      mi    epc sepc.lv sepc.all sepc.nox
## 1 sbelong1 ~~ sbelong3 877.423 -0.558  -0.558   -0.293   -0.293
## 2 sbelong2 ~~ sbelong4 877.427 -0.482  -0.482   -0.225   -0.225
## 3 sbelong3 ~~ sbelong4 292.729  0.256   0.256    0.122    0.122
```

```
sb <- 'sb =~ sbelong1 + sbelong2 + sbelong3 + sbelong4
       sbelong1 ~~ sbelong3'
sbmod <- cfa(sb, estimator = "MLR", data = pls2, std.lv = T, cluster = "country")
fitmeasures(sbmod, fit.measures = c("chisq.scaled", "df.scaled", "cfi.robust", "rmsea.ci.upper.robust", "srmr"))
```

```
##          chisq.scaled             df.scaled            cfi.robust 
##                 0.015                 1.000                 1.000 
## rmsea.ci.upper.robust                  srmr 
##                 0.000                 0.000
```

```
semPaths(sbmod, what = "std", edge.label.cex = 1.2, label.cex = 1.2)
```

```
reliability(sbmod)
```

```
##               sb
## alpha  0.8505697
## omega  0.8828070
## omega2 0.8828070
## omega3 0.8828221
## avevar 0.6193849
```

```
sbmodc <- cfa(sb, estimator = "MLR", data = pls2, std.lv = T, cluster = "country", group = "cgr")
```

```
## Warning in lav_model_vcov(lavmodel = lavmodel, lavsamplestats = lavsamplestats, : lavaan WARNING:
##     The variance-covariance matrix of the estimated parameters (vcov)
##     does not appear to be positive definite! The smallest eigenvalue
##     (= -7.237423e-16) is smaller than zero. This may be a symptom that
##     the model is not identified.
```

```
sbmodw <- cfa(sb, estimator = "MLR", data = pls2, std.lv = T, cluster = "country", group = "cgr", group.equal = "loadings")
```

```
## Warning in lav_model_vcov(lavmodel = lavmodel, lavsamplestats = lavsamplestats, : lavaan WARNING:
##     The variance-covariance matrix of the estimated parameters (vcov)
##     does not appear to be positive definite! The smallest eigenvalue
##     (= 5.837247e-17) is close to zero. This may be a symptom that the
##     model is not identified.
```

```
sbmods <- cfa(sb, estimator = "MLR", data = pls2, std.lv = T, cluster = "country", group = "cgr", group.equal = c("loadings", "intercepts"))
```

```
## Warning in lav_model_vcov(lavmodel = lavmodel, lavsamplestats = lavsamplestats, : lavaan WARNING:
##     The variance-covariance matrix of the estimated parameters (vcov)
##     does not appear to be positive definite! The smallest eigenvalue
##     (= 1.076553e-14) is close to zero. This may be a symptom that the
##     model is not identified.
```

```
summary(compareFit(sbmodc, sbmodw, sbmods, nested = T))
```

```
## ################### Nested Model Comparison #########################
## Scaled Chi-Squared Difference Test (method = "satorra.bentler.2001")
## 
## lavaan NOTE:
##     The "Chisq" column contains standard test statistics, not the
##     robust test that should be reported per model. A robust difference
##     test is a function of two standard (not robust) statistics.
##  
##        Df    AIC    BIC    Chisq Chisq diff Df diff Pr(>Chisq)    
## sbmodc  8 701353 702256   78.186                                  
## sbmodw 29 701708 702429  475.302     59.465      21  1.539e-05 ***
## sbmods 50 702877 703416 1686.860     85.904      21  8.134e-10 ***
## ---
## Signif. codes:  0 '***' 0.001 '**' 0.01 '*' 0.05 '.' 0.1 ' ' 1
## 
## ####################### Model Fit Indices ###########################
##        chisq.scaled df.scaled pvalue.scaled rmsea.robust cfi.robust tli.robust
## sbmodc      15.130†         8          .057        .029†     1.000†     0.997†
## sbmodw      75.909         29          .000        .043      0.996       .994 
## sbmods     176.539         50          .000        .067       .984       .985 
##         srmr         aic         bic
## sbmodc .004† 701352.676† 702255.810†
## sbmodw .030  701707.791  702428.562 
## sbmods .041  702877.350  703415.757 
## 
## ################## Differences in Fit Indices #######################
##                 df.scaled rmsea.robust cfi.robust tli.robust  srmr      aic
## sbmodw - sbmodc        21        0.014     -0.003     -0.003 0.026  355.116
## sbmods - sbmodw        21        0.023     -0.012     -0.009 0.011 1169.558
##                     bic
## sbmodw - sbmodc 172.752
## sbmods - sbmodw 987.195
```

```
miobj <- modindices(sbmodw, free.remove = F)
miobj %>% filter(op == "=~") %>% group_by(lhs, rhs) %>% summarize(mi = mean(mi)) %>% top_n(3, mi)
```

```
## `summarise()` has grouped output by 'lhs'. You can override using the `.groups` argument.
```

```
## # A tibble: 3 x 3
## # Groups:   lhs [1]
##   lhs   rhs         mi
##   <chr> <chr>    <dbl>
## 1 sb    sbelong1 12.4 
## 2 sb    sbelong3  7.00
## 3 sb    sbelong4 24.5
```

```
sbmodw <- cfa(sb, estimator = "MLR", data = pls2, std.lv = T, cluster = "country", group = "cgr", group.equal = "loadings", group.partial = c("sb =~ sbelong4"))
```

```
## Warning in lav_model_vcov(lavmodel = lavmodel, lavsamplestats = lavsamplestats, : lavaan WARNING:
##     The variance-covariance matrix of the estimated parameters (vcov)
##     does not appear to be positive definite! The smallest eigenvalue
##     (= -8.840667e-17) is smaller than zero. This may be a symptom that
##     the model is not identified.
```

```
sbmods <- cfa(sb, estimator = "MLR", data = pls2, std.lv = T, cluster = "country", group = "cgr", group.equal = c("loadings", "intercepts"), group.partial = c("sb =~ sbelong4"))
```

```
## Warning in lav_model_vcov(lavmodel = lavmodel, lavsamplestats = lavsamplestats, : lavaan WARNING:
##     The variance-covariance matrix of the estimated parameters (vcov)
##     does not appear to be positive definite! The smallest eigenvalue
##     (= 2.107653e-15) is close to zero. This may be a symptom that the
##     model is not identified.
```

```
summary(compareFit(sbmodc, sbmodw, sbmods, nested = T))
```

```
## ################### Nested Model Comparison #########################
## Scaled Chi-Squared Difference Test (method = "satorra.bentler.2001")
## 
## lavaan NOTE:
##     The "Chisq" column contains standard test statistics, not the
##     robust test that should be reported per model. A robust difference
##     test is a function of two standard (not robust) statistics.
##  
##        Df    AIC    BIC    Chisq Chisq diff Df diff Pr(>Chisq)    
## sbmodc  8 701353 702256   78.186                                  
## sbmodw 22 701498 702280  251.780     41.504      14  0.0001481 ***
## sbmods 43 702523 703122 1318.163     70.440      21  2.987e-07 ***
## ---
## Signif. codes:  0 '***' 0.001 '**' 0.01 '*' 0.05 '.' 0.1 ' ' 1
## 
## ####################### Model Fit Indices ###########################
##        chisq.scaled df.scaled pvalue.scaled rmsea.robust cfi.robust tli.robust
## sbmodc      15.130†         8          .057        .029†     1.000†     0.997†
## sbmodw      55.449         22          .000        .036      0.998      0.996 
## sbmods     135.661         43          .000        .062       .988       .987 
##         srmr         aic         bic
## sbmodc .004† 701352.676† 702255.810†
## sbmodw .019  701498.269  702279.828 
## sbmods .034  702522.652  703121.847 
## 
## ################## Differences in Fit Indices #######################
##                 df.scaled rmsea.robust cfi.robust tli.robust  srmr      aic
## sbmodw - sbmodc        14        0.007     -0.001     -0.001 0.015  145.594
## sbmods - sbmodw        21        0.026     -0.010     -0.009 0.015 1024.383
##                     bic
## sbmodw - sbmodc  24.018
## sbmods - sbmodw 842.019
```

```
miobj <- modindices(sbmods, free.remove = F)
miobj %>% filter(op == "~1") %>% group_by(lhs, rhs) %>% summarize(mi = mean(mi)) %>% top_n(3, mi)
```

```
## `summarise()` has grouped output by 'lhs'. You can override using the `.groups` argument.
```

```
## # A tibble: 5 x 3
## # Groups:   lhs [5]
##   lhs      rhs      mi
##   <chr>    <chr> <dbl>
## 1 sb       ""     36.8
## 2 sbelong1 ""     32.1
## 3 sbelong2 ""     39.8
## 4 sbelong3 ""     46.5
## 5 sbelong4 ""     41.0
```

```
sbmods <- cfa(sb, estimator = "MLR", data = pls2, std.lv = T, cluster = "country", group = "cgr", group.equal = c("loadings", "intercepts"), group.partial = c("sb =~ sbelong4", "sbelong3 ~ 1"))
```

```
## Warning in lav_model_vcov(lavmodel = lavmodel, lavsamplestats = lavsamplestats, : lavaan WARNING:
##     The variance-covariance matrix of the estimated parameters (vcov)
##     does not appear to be positive definite! The smallest eigenvalue
##     (= 7.902368e-16) is close to zero. This may be a symptom that the
##     model is not identified.
```

```
summary(compareFit(sbmodc, sbmodw, sbmods, nested = T))
```

```
## ################### Nested Model Comparison #########################
## Scaled Chi-Squared Difference Test (method = "satorra.bentler.2001")
## 
## lavaan NOTE:
##     The "Chisq" column contains standard test statistics, not the
##     robust test that should be reported per model. A robust difference
##     test is a function of two standard (not robust) statistics.
##  
##        Df    AIC    BIC   Chisq Chisq diff Df diff Pr(>Chisq)    
## sbmodc  8 701353 702256  78.186                                  
## sbmodw 22 701498 702280 251.780     41.504      14  0.0001481 ***
## sbmods 36 702106 702766 887.377     36.423      14  0.0009014 ***
## ---
## Signif. codes:  0 '***' 0.001 '**' 0.01 '*' 0.05 '.' 0.1 ' ' 1
## 
## ####################### Model Fit Indices ###########################
##        chisq.scaled df.scaled pvalue.scaled rmsea.robust cfi.robust tli.robust
## sbmodc      15.130†         8          .057        .029†     1.000†     0.997†
## sbmodw      55.449         22          .000        .036      0.998      0.996 
## sbmods      92.811         36          .000        .053       .993       .991 
##         srmr         aic         bic
## sbmodc .004† 701352.676† 702255.810†
## sbmodw .019  701498.269  702279.828 
## sbmods .032  702105.867  702765.849 
## 
## ################## Differences in Fit Indices #######################
##                 df.scaled rmsea.robust cfi.robust tli.robust  srmr     aic
## sbmodw - sbmodc        14        0.007     -0.001     -0.001 0.015 145.594
## sbmods - sbmodw        14        0.017     -0.005     -0.005 0.013 607.597
##                     bic
## sbmodw - sbmodc  24.018
## sbmods - sbmodw 486.021
```

```
belongs <- bind_rows(estimate_lavaan_ten_berge_per_group(sbmods))
```

### Self-control

```
pls2$slfcont3 <- 10-pls2$slfcont3
pls2$slfcont4 <- 10-pls2$slfcont4
tsc <- 'tsc =~ slfcont1 + slfcont2 + slfcont3 + slfcont4'
tscmod <- cfa(tsc, estimator = "MLR", data = pls2, std.lv = T, cluster = "country")
fitmeasures(tscmod, fit.measures = c("chisq.scaled", "df.scaled", "cfi.robust", "rmsea.ci.upper.robust", "srmr"))
```

```
##          chisq.scaled             df.scaled            cfi.robust 
##               204.964                 2.000                 0.906 
## rmsea.ci.upper.robust                  srmr 
##                 0.176                 0.040
```

```
miobj <- modindices(tscmod)
top_n(miobj, 3, mi)
```

```
##        lhs op      rhs       mi    epc sepc.lv sepc.all sepc.nox
## 1 slfcont1 ~~ slfcont2 1349.616  1.394   1.394    0.424    0.424
## 2 slfcont1 ~~ slfcont4 1716.079 -1.803  -1.803   -0.366   -0.366
## 3 slfcont2 ~~ slfcont3 1716.086 -1.350  -1.350   -0.333   -0.333
```

```
tsc <- 'tsc =~ slfcont1 + slfcont2 + slfcont3 + slfcont4
        slfcont1 ~~ slfcont2'
tscmod <- cfa(tsc, estimator = "MLR", data = pls2, std.lv = T, cluster = "country")
fitmeasures(tscmod, fit.measures = c("chisq.scaled", "df.scaled", "cfi.robust", "rmsea.ci.upper.robust", "srmr"))
```

```
##          chisq.scaled             df.scaled            cfi.robust 
##               256.007                 1.000                 0.961 
## rmsea.ci.upper.robust                  srmr 
##                 0.158                 0.024
```

```
semPaths(tscmod, what = "std", edge.label.cex = 1.2, label.cex = 1.2)
```

```
reliability(tscmod)
```

```
##              tsc
## alpha  0.6406318
## omega  0.5978596
## omega2 0.5978596
## omega3 0.5971970
## avevar 0.3061735
```

```
tscmodc <- cfa(tsc, estimator = "MLR", data = pls2, std.lv = T, cluster = "country", group = "cgr")
```

```
## Warning in lav_model_vcov(lavmodel = lavmodel, lavsamplestats = lavsamplestats, : lavaan WARNING:
##     The variance-covariance matrix of the estimated parameters (vcov)
##     does not appear to be positive definite! The smallest eigenvalue
##     (= -3.479893e-16) is smaller than zero. This may be a symptom that
##     the model is not identified.
```

```
tscmodw <- cfa(tsc, estimator = "MLR", data = pls2, std.lv = T, cluster = "country", group = "cgr", group.equal = "loadings")
```

```
## Warning in lav_model_vcov(lavmodel = lavmodel, lavsamplestats = lavsamplestats, : lavaan WARNING:
##     The variance-covariance matrix of the estimated parameters (vcov)
##     does not appear to be positive definite! The smallest eigenvalue
##     (= -2.303417e-16) is smaller than zero. This may be a symptom that
##     the model is not identified.
```

```
tscmods <- cfa(tsc, estimator = "MLR", data = pls2, std.lv = T, cluster = "country", group = "cgr", group.equal = c("loadings", "intercepts"))
```

```
## Warning in lav_model_vcov(lavmodel = lavmodel, lavsamplestats = lavsamplestats, : lavaan WARNING:
##     The variance-covariance matrix of the estimated parameters (vcov)
##     does not appear to be positive definite! The smallest eigenvalue
##     (= 3.506096e-14) is close to zero. This may be a symptom that the
##     model is not identified.
```

```
summary(compareFit(tscmodc, tscmodw, tscmods, nested = T))
```

```
## ################### Nested Model Comparison #########################
## Scaled Chi-Squared Difference Test (method = "satorra.bentler.2001")
## 
## lavaan NOTE:
##     The "Chisq" column contains standard test statistics, not the
##     robust test that should be reported per model. A robust difference
##     test is a function of two standard (not robust) statistics.
##  
##         Df    AIC    BIC   Chisq Chisq diff Df diff Pr(>Chisq)    
## tscmodc  8 796801 797704  874.18                                  
## tscmodw 29 797005 797726 1120.46     39.785      21   0.007897 ** 
## tscmods 50 799610 800149 3767.71    117.316      21  2.213e-15 ***
## ---
## Signif. codes:  0 '***' 0.001 '**' 0.01 '*' 0.05 '.' 0.1 ' ' 1
## 
## ####################### Model Fit Indices ###########################
##         chisq.scaled df.scaled pvalue.scaled rmsea.robust cfi.robust tli.robust
## tscmodc     418.590          8          .000        .140       .964†      .782 
## tscmodw     221.493†        29          .000        .078†      .959       .932†
## tscmods     303.570         50          .000        .107       .867       .872 
##          srmr         aic         bic
## tscmodc .023† 796800.876† 797704.010†
## tscmodw .031  797005.153  797725.924 
## tscmods .059  799610.404  800148.810 
## 
## ################## Differences in Fit Indices #######################
##                   df.scaled rmsea.robust cfi.robust tli.robust  srmr      aic
## tscmodw - tscmodc        21       -0.062     -0.005       0.15 0.009  204.277
## tscmods - tscmodw        21        0.029     -0.092      -0.06 0.027 2605.251
##                        bic
## tscmodw - tscmodc   21.913
## tscmods - tscmodw 2422.887
```

```
miobj <- modindices(tscmods, free.remove = F)
miobj %>% filter(op == "~1") %>% group_by(lhs, rhs) %>% summarize(mi = mean(mi)) %>% top_n(3, mi)
```

```
## `summarise()` has grouped output by 'lhs'. You can override using the `.groups` argument.
```

```
## # A tibble: 5 x 3
## # Groups:   lhs [5]
##   lhs      rhs        mi
##   <chr>    <chr>   <dbl>
## 1 slfcont1 ""    8.21e+1
## 2 slfcont2 ""    1.73e+2
## 3 slfcont3 ""    6.73e+1
## 4 slfcont4 ""    5.23e+1
## 5 tsc      ""    7.12e-9
```

```
tscmods <- cfa(tsc, estimator = "MLR", data = pls2, std.lv = T, cluster = "country", group = "cgr", group.equal = c("loadings", "intercepts"), group.partial = c("slfcont2 ~ 1"))
```

```
## Warning in lav_model_vcov(lavmodel = lavmodel, lavsamplestats = lavsamplestats, : lavaan WARNING:
##     The variance-covariance matrix of the estimated parameters (vcov)
##     does not appear to be positive definite! The smallest eigenvalue
##     (= 1.556611e-14) is close to zero. This may be a symptom that the
##     model is not identified.
```

```
summary(compareFit(tscmodc, tscmodw, tscmods, nested = T))
```

```
## ################### Nested Model Comparison #########################
## Scaled Chi-Squared Difference Test (method = "satorra.bentler.2001")
## 
## lavaan NOTE:
##     The "Chisq" column contains standard test statistics, not the
##     robust test that should be reported per model. A robust difference
##     test is a function of two standard (not robust) statistics.
##  
##         Df    AIC    BIC   Chisq Chisq diff Df diff Pr(>Chisq)    
## tscmodc  8 796801 797704  874.18                                  
## tscmodw 29 797005 797726 1120.46     39.785      21   0.007897 ** 
## tscmods 43 798038 798638 2181.79     45.485      14  3.401e-05 ***
## ---
## Signif. codes:  0 '***' 0.001 '**' 0.01 '*' 0.05 '.' 0.1 ' ' 1
## 
## ####################### Model Fit Indices ###########################
##         chisq.scaled df.scaled pvalue.scaled rmsea.robust cfi.robust tli.robust
## tscmodc     418.590          8          .000        .140       .964†      .782 
## tscmodw     221.493         29          .000        .078†      .959       .932†
## tscmods     198.190†        43          .000        .085       .928       .919 
##          srmr         aic         bic
## tscmodc .023† 796800.876† 797704.010†
## tscmodw .031  797005.153  797725.924 
## tscmods .045  798038.486  798637.680 
## 
## ################## Differences in Fit Indices #######################
##                   df.scaled rmsea.robust cfi.robust tli.robust  srmr      aic
## tscmodw - tscmodc        21       -0.062     -0.005      0.150 0.009  204.277
## tscmods - tscmodw        14        0.007     -0.031     -0.013 0.014 1033.332
##                       bic
## tscmodw - tscmodc  21.913
## tscmods - tscmodw 911.757
```

```
miobj <- modindices(tscmods, free.remove = F)
miobj %>% filter(op == "~1") %>% group_by(lhs, rhs) %>% summarize(mi = mean(mi)) %>% top_n(3, mi)
```

```
## `summarise()` has grouped output by 'lhs'. You can override using the `.groups` argument.
```

```
## # A tibble: 5 x 3
## # Groups:   lhs [5]
##   lhs      rhs         mi
##   <chr>    <chr>    <dbl>
## 1 slfcont1 ""    4.84e+ 1
## 2 slfcont2 ""    5.80e-10
## 3 slfcont3 ""    4.55e+ 1
## 4 slfcont4 ""    5.44e+ 1
## 5 tsc      ""    2.30e- 8
```

```
tscmods <- cfa(tsc, estimator = "MLR", data = pls2, std.lv = T, cluster = "country", group = "cgr", group.equal = c("loadings", "intercepts"), group.partial = c("slfcont2 ~ 1", "slfcont4 ~ 1"))
```

```
## Warning in lav_model_vcov(lavmodel = lavmodel, lavsamplestats = lavsamplestats, : lavaan WARNING:
##     The variance-covariance matrix of the estimated parameters (vcov)
##     does not appear to be positive definite! The smallest eigenvalue
##     (= 8.157437e-16) is close to zero. This may be a symptom that the
##     model is not identified.
```

```
summary(compareFit(tscmodc, tscmodw, tscmods, nested = T))
```

```
## ################### Nested Model Comparison #########################
## Scaled Chi-Squared Difference Test (method = "satorra.bentler.2001")
## 
## lavaan NOTE:
##     The "Chisq" column contains standard test statistics, not the
##     robust test that should be reported per model. A robust difference
##     test is a function of two standard (not robust) statistics.
##  
##         Df    AIC    BIC   Chisq Chisq diff Df diff Pr(>Chisq)   
## tscmodc  8 796801 797704  874.18                                 
## tscmodw 29 797005 797726 1120.46     39.785      21   0.007897 **
## tscmods 36 797406 798066 1535.17     19.117       7   0.007829 **
## ---
## Signif. codes:  0 '***' 0.001 '**' 0.01 '*' 0.05 '.' 0.1 ' ' 1
## 
## ####################### Model Fit Indices ###########################
##         chisq.scaled df.scaled pvalue.scaled rmsea.robust cfi.robust tli.robust
## tscmodc     418.590          8          .000        .140       .964†      .782 
## tscmodw     221.493         29          .000        .078†      .959       .932†
## tscmods     185.112†        36          .000        .079       .948       .930 
##          srmr         aic         bic
## tscmodc .023† 796800.876† 797704.010†
## tscmodw .031  797005.153  797725.924 
## tscmods .039  797405.871  798065.854 
## 
## ################## Differences in Fit Indices #######################
##                   df.scaled rmsea.robust cfi.robust tli.robust  srmr     aic
## tscmodw - tscmodc        21       -0.062     -0.005      0.150 0.009 204.277
## tscmods - tscmodw         7        0.001     -0.011     -0.002 0.008 400.718
##                       bic
## tscmodw - tscmodc  21.913
## tscmods - tscmodw 339.930
```

```
selfcontrols <- bind_rows(estimate_lavaan_ten_berge_per_group(tscmods))
```

### Narcissism

```
nar <- 'nar1 =~ narc2 + narc4 + narc5 
        nar2 =~ narc1 + narc3 + narc6'
narmod <- cfa(nar, estimator = "MLR", data = pls2, std.lv = T, cluster = "country")
fitmeasures(narmod, fit.measures = c("chisq.scaled", "df.scaled", "cfi.robust", "rmsea.ci.upper.robust", "srmr"))
```

```
##          chisq.scaled             df.scaled            cfi.robust 
##               302.825                 8.000                 0.956 
## rmsea.ci.upper.robust                  srmr 
##                 0.103                 0.039
```

```
miobj <- modindices(narmod)
top_n(miobj, 3, mi)
```

```
##     lhs op   rhs       mi    epc sepc.lv sepc.all sepc.nox
## 1  nar1 =~ narc1 2181.008  1.410   1.410    0.496    0.496
## 2  nar1 =~ narc3 1068.467 -0.981  -0.981   -0.332   -0.332
## 3 narc3 ~~ narc6 2181.005  1.979   1.979    0.362    0.362
```

```
nar <- 'admire =~ narc2 + narc4 + narc5 
        rival =~ narc1 + narc3 + narc6
        narc3 ~~ narc6'
narmod <- cfa(nar, estimator = "MLR", data = pls2, std.lv = T, cluster = "country")
fitmeasures(narmod, fit.measures = c("chisq.scaled", "df.scaled", "cfi.robust", "rmsea.ci.upper.robust", "srmr"))
```

```
##          chisq.scaled             df.scaled            cfi.robust 
##                83.097                 7.000                 0.986 
## rmsea.ci.upper.robust                  srmr 
##                 0.068                 0.020
```

```
semPaths(narmod, what = "std", edge.label.cex = 1.2, label.cex = 1.2)
```

```
reliability(narmod)
```

```
##           admire     rival
## alpha  0.7881756 0.6547927
## omega  0.7875758 0.5538435
## omega2 0.7875758 0.5538435
## omega3 0.7865607 0.5549980
## avevar 0.5529436 0.3486456
```

```
narmodc <- cfa(nar, estimator = "MLR", data = pls2, std.lv = T, cluster = "country", group = "cgr")
```

```
## Warning in lav_model_vcov(lavmodel = lavmodel, lavsamplestats = lavsamplestats, : lavaan WARNING:
##     The variance-covariance matrix of the estimated parameters (vcov)
##     does not appear to be positive definite! The smallest eigenvalue
##     (= -2.554828e-15) is smaller than zero. This may be a symptom that
##     the model is not identified.
```

```
narmodw <- cfa(nar, estimator = "MLR", data = pls2, std.lv = T, cluster = "country", group = "cgr", group.equal = "loadings")
```

```
## Warning in lav_model_vcov(lavmodel = lavmodel, lavsamplestats = lavsamplestats, : lavaan WARNING:
##     The variance-covariance matrix of the estimated parameters (vcov)
##     does not appear to be positive definite! The smallest eigenvalue
##     (= -2.003465e-15) is smaller than zero. This may be a symptom that
##     the model is not identified.
```

```
narmods <- cfa(nar, estimator = "MLR", data = pls2, std.lv = T, cluster = "country", group = "cgr", group.equal = c("loadings", "intercepts"))
```

```
## Warning in lav_model_vcov(lavmodel = lavmodel, lavsamplestats = lavsamplestats, : lavaan WARNING:
##     The variance-covariance matrix of the estimated parameters (vcov)
##     does not appear to be positive definite! The smallest eigenvalue
##     (= 9.094308e-15) is close to zero. This may be a symptom that the
##     model is not identified.
```

```
summary(compareFit(narmodc, narmodw, narmods, nested = T))
```

```
## ################### Nested Model Comparison #########################
## Scaled Chi-Squared Difference Test (method = "satorra.bentler.2001")
## 
## lavaan NOTE:
##     The "Chisq" column contains standard test statistics, not the
##     robust test that should be reported per model. A robust difference
##     test is a function of two standard (not robust) statistics.
##  
##          Df     AIC     BIC  Chisq Chisq diff Df diff Pr(>Chisq)    
## narmodc  56 1212987 1214376 1684.6                                  
## narmodw  84 1213721 1214867 2474.3     445.81      28  < 2.2e-16 ***
## narmods 112 1218905 1219808 7714.8     145.23      28  < 2.2e-16 ***
## ---
## Signif. codes:  0 '***' 0.001 '**' 0.01 '*' 0.05 '.' 0.1 ' ' 1
## 
## ####################### Model Fit Indices ###########################
##         chisq.scaled df.scaled pvalue.scaled rmsea.scaled cfi.scaled tli.scaled
## narmodc     448.112†        56          .000        .036         NA         NA 
## narmodw     799.015         84          .000        .039         NA         NA 
## narmods     680.087        112          .000        .030†        NA         NA 
##          srmr          aic          bic
## narmodc .024† 1212986.866† 1214376.303†
## narmodw .040  1213720.541  1214866.827 
## narmods .066  1218905.104  1219808.239 
## 
## ################## Differences in Fit Indices #######################
##                   df.scaled rmsea.scaled cfi.scaled tli.scaled  srmr      aic
## narmodw - narmodc        28        0.004         NA         NA 0.017  733.675
## narmods - narmodw        28       -0.009         NA         NA 0.025 5184.563
##                        bic
## narmodw - narmodc  490.524
## narmods - narmodw 4941.412
```

```
fitmeasures(narmodc, fit.measures = c("chisq.scaled", "df.scaled", "cfi.robust", "rmsea.robust", "srmr"))
```

```
## chisq.scaled    df.scaled   cfi.robust rmsea.robust         srmr 
##      448.112       56.000           NA        0.069        0.024
```

```
fitmeasures(narmodw, fit.measures = c("chisq.scaled", "df.scaled", "cfi.robust", "rmsea.robust", "srmr"))
```

```
## chisq.scaled    df.scaled   cfi.robust rmsea.robust         srmr 
##      799.015       84.000           NA        0.070        0.040
```

```
fitmeasures(narmods, fit.measures = c("chisq.scaled", "df.scaled", "cfi.robust", "rmsea.robust", "srmr"))
```

```
## chisq.scaled    df.scaled   cfi.robust rmsea.robust         srmr 
##      680.087      112.000           NA        0.103        0.066
```

```
miobj <- modindices(narmodw, free.remove = F)
miobj %>% filter(op == "=~") %>% group_by(lhs, rhs) %>% summarize(mi = mean(mi)) %>% top_n(3, mi)
```

```
## `summarise()` has grouped output by 'lhs'. You can override using the `.groups` argument.
```

```
## # A tibble: 6 x 3
## # Groups:   lhs [2]
##   lhs    rhs      mi
##   <chr>  <chr> <dbl>
## 1 admire narc1  43.2
## 2 admire narc3  41.8
## 3 admire narc5  24.0
## 4 rival  narc2  60.5
## 5 rival  narc4  44.6
## 6 rival  narc5  66.0
```

```
narmodw <- cfa(nar, estimator = "MLR", data = pls2, std.lv = T, cluster = "country", group = "cgr", group.equal = "loadings", group.partial = c("admire =~ narc5"))
```

```
## Warning in lav_model_vcov(lavmodel = lavmodel, lavsamplestats = lavsamplestats, : lavaan WARNING:
##     The variance-covariance matrix of the estimated parameters (vcov)
##     does not appear to be positive definite! The smallest eigenvalue
##     (= -1.297370e-15) is smaller than zero. This may be a symptom that
##     the model is not identified.
```

```
narmods <- cfa(nar, estimator = "MLR", data = pls2, std.lv = T, cluster = "country", group = "cgr", group.equal = c("loadings", "intercepts"), group.partial = c("admire =~ narc5"))
```

```
## Warning in lav_model_vcov(lavmodel = lavmodel, lavsamplestats = lavsamplestats, : lavaan WARNING:
##     The variance-covariance matrix of the estimated parameters (vcov)
##     does not appear to be positive definite! The smallest eigenvalue
##     (= 2.715823e-15) is close to zero. This may be a symptom that the
##     model is not identified.
```

```
summary(compareFit(narmodc, narmodw, narmods, nested = T))
```

```
## ################### Nested Model Comparison #########################
## Scaled Chi-Squared Difference Test (method = "satorra.bentler.2001")
## 
## lavaan NOTE:
##     The "Chisq" column contains standard test statistics, not the
##     robust test that should be reported per model. A robust difference
##     test is a function of two standard (not robust) statistics.
##  
##          Df     AIC     BIC  Chisq Chisq diff Df diff Pr(>Chisq)    
## narmodc  56 1212987 1214376 1684.6                                  
## narmodw  77 1213466 1214673 2205.7     510.50      21  < 2.2e-16 ***
## narmods 105 1218053 1219017 6849.0     148.79      28  < 2.2e-16 ***
## ---
## Signif. codes:  0 '***' 0.001 '**' 0.01 '*' 0.05 '.' 0.1 ' ' 1
## 
## ####################### Model Fit Indices ###########################
##         chisq.scaled df.scaled pvalue.scaled rmsea.scaled cfi.scaled tli.scaled
## narmodc     448.112†        56          .000        .036         NA         NA 
## narmodw     732.202         77          .000        .039         NA         NA 
## narmods     650.363        105          .000        .031†        NA         NA 
##          srmr          aic          bic
## narmodc .024† 1212986.866† 1214376.303†
## narmodw .035  1213465.996  1214673.069 
## narmods .063  1218053.309  1219017.230 
## 
## ################## Differences in Fit Indices #######################
##                   df.scaled rmsea.scaled cfi.scaled tli.scaled  srmr      aic
## narmodw - narmodc        21        0.004         NA         NA 0.012  479.130
## narmods - narmodw        28       -0.009         NA         NA 0.027 4587.312
##                        bic
## narmodw - narmodc  296.766
## narmods - narmodw 4344.161
```

```
fitmeasures(narmodc, fit.measures = c("chisq.scaled", "df.scaled", "cfi.robust", "rmsea.robust", "srmr"))
```

```
## chisq.scaled    df.scaled   cfi.robust rmsea.robust         srmr 
##      448.112       56.000           NA        0.069        0.024
```

```
fitmeasures(narmodw, fit.measures = c("chisq.scaled", "df.scaled", "cfi.robust", "rmsea.robust", "srmr"))
```

```
## chisq.scaled    df.scaled   cfi.robust rmsea.robust         srmr 
##      732.202       77.000           NA        0.069        0.035
```

```
fitmeasures(narmods, fit.measures = c("chisq.scaled", "df.scaled", "cfi.robust", "rmsea.robust", "srmr"))
```

```
## chisq.scaled    df.scaled   cfi.robust rmsea.robust         srmr 
##      650.363      105.000           NA        0.100        0.063
```

```
miobj <- modindices(narmods, free.remove = F)
miobj %>% filter(op == "~1") %>% group_by(lhs, rhs) %>% summarize(mi = mean(mi)) %>% top_n(3, mi)
```

```
## `summarise()` has grouped output by 'lhs'. You can override using the `.groups` argument.
```

```
## # A tibble: 8 x 3
## # Groups:   lhs [8]
##   lhs    rhs        mi
##   <chr>  <chr>   <dbl>
## 1 admire ""    6.23e+1
## 2 narc1  ""    1.35e+2
## 3 narc2  ""    7.26e+1
## 4 narc3  ""    1.00e+2
## 5 narc4  ""    8.24e+1
## 6 narc5  ""    6.18e+1
## 7 narc6  ""    1.09e+2
## 8 rival  ""    2.30e-8
```

```
narmods <- cfa(nar, estimator = "MLR", data = pls2, std.lv = T, cluster = "country", group = "cgr", group.equal = c("loadings", "intercepts"), group.partial = c("admire =~ narc5", "narc1 ~ 1"))
```

```
## Warning in lav_model_vcov(lavmodel = lavmodel, lavsamplestats = lavsamplestats, : lavaan WARNING:
##     The variance-covariance matrix of the estimated parameters (vcov)
##     does not appear to be positive definite! The smallest eigenvalue
##     (= 4.067435e-15) is close to zero. This may be a symptom that the
##     model is not identified.
```

```
summary(compareFit(narmodc, narmodw, narmods, nested = T))
```

```
## ################### Nested Model Comparison #########################
## Scaled Chi-Squared Difference Test (method = "satorra.bentler.2001")
## 
## lavaan NOTE:
##     The "Chisq" column contains standard test statistics, not the
##     robust test that should be reported per model. A robust difference
##     test is a function of two standard (not robust) statistics.
##  
##         Df     AIC     BIC  Chisq Chisq diff Df diff Pr(>Chisq)    
## narmodc 56 1212987 1214376 1684.6                                  
## narmodw 77 1213466 1214673 2205.7     510.50      21  < 2.2e-16 ***
## narmods 98 1215942 1216967 4723.7      81.23      21  5.027e-09 ***
## ---
## Signif. codes:  0 '***' 0.001 '**' 0.01 '*' 0.05 '.' 0.1 ' ' 1
## 
## ####################### Model Fit Indices ###########################
##         chisq.scaled df.scaled pvalue.scaled rmsea.scaled cfi.scaled tli.scaled
## narmodc     448.112†        56          .000        .036         NA         NA 
## narmodw     732.202         77          .000        .039         NA         NA 
## narmods     524.299         98          .000        .028†        NA         NA 
##          srmr          aic          bic
## narmodc .024† 1212986.866† 1214376.303†
## narmodw .035  1213465.996  1214673.069 
## narmods .051  1215942.036  1216966.745 
## 
## ################## Differences in Fit Indices #######################
##                   df.scaled rmsea.scaled cfi.scaled tli.scaled  srmr      aic
## narmodw - narmodc        21        0.004         NA         NA 0.012  479.130
## narmods - narmodw        21       -0.011         NA         NA 0.016 2476.039
##                        bic
## narmodw - narmodc  296.766
## narmods - narmodw 2293.676
```

```
fitmeasures(narmodc, fit.measures = c("chisq.scaled", "df.scaled", "cfi.robust", "rmsea.robust", "srmr"))
```

```
## chisq.scaled    df.scaled   cfi.robust rmsea.robust         srmr 
##      448.112       56.000           NA        0.069        0.024
```

```
fitmeasures(narmodw, fit.measures = c("chisq.scaled", "df.scaled", "cfi.robust", "rmsea.robust", "srmr"))
```

```
## chisq.scaled    df.scaled   cfi.robust rmsea.robust         srmr 
##      732.202       77.000           NA        0.069        0.035
```

```
fitmeasures(narmods, fit.measures = c("chisq.scaled", "df.scaled", "cfi.robust", "rmsea.robust", "srmr"))
```

```
## chisq.scaled    df.scaled   cfi.robust rmsea.robust         srmr 
##      524.299       98.000           NA        0.085        0.051
```

```
miobj <- modindices(narmods, free.remove = F)
miobj %>% filter(op == "~1") %>% group_by(lhs, rhs) %>% summarize(mi = mean(mi)) %>% top_n(3, mi)
```

```
## `summarise()` has grouped output by 'lhs'. You can override using the `.groups` argument.
```

```
## # A tibble: 8 x 3
## # Groups:   lhs [8]
##   lhs    rhs        mi
##   <chr>  <chr>   <dbl>
## 1 admire ""    6.36e+1
## 2 narc1  ""    1.66e-9
## 3 narc2  ""    7.27e+1
## 4 narc3  ""    3.62e+1
## 5 narc4  ""    8.24e+1
## 6 narc5  ""    6.19e+1
## 7 narc6  ""    7.20e+1
## 8 rival  ""    2.46e-9
```

```
narmods <- cfa(nar, estimator = "MLR", data = pls2, std.lv = T, cluster = "country", group = "cgr", group.equal = c("loadings", "intercepts"), group.partial = c("admire =~ narc5", "narc1 ~ 1", "narc4 ~ 1"))
```

```
## Warning in lav_model_vcov(lavmodel = lavmodel, lavsamplestats = lavsamplestats, : lavaan WARNING:
##     The variance-covariance matrix of the estimated parameters (vcov)
##     does not appear to be positive definite! The smallest eigenvalue
##     (= -3.196889e-15) is smaller than zero. This may be a symptom that
##     the model is not identified.
```

```
summary(compareFit(narmodc, narmodw, narmods, nested = T))
```

```
## ################### Nested Model Comparison #########################
## Scaled Chi-Squared Difference Test (method = "satorra.bentler.2001")
## 
## lavaan NOTE:
##     The "Chisq" column contains standard test statistics, not the
##     robust test that should be reported per model. A robust difference
##     test is a function of two standard (not robust) statistics.
##  
##         Df     AIC     BIC  Chisq Chisq diff Df diff Pr(>Chisq)    
## narmodc 56 1212987 1214376 1684.6                                  
## narmodw 77 1213466 1214673 2205.7     510.50      21  < 2.2e-16 ***
## narmods 91 1215090 1216175 3857.7      47.43      14  1.633e-05 ***
## ---
## Signif. codes:  0 '***' 0.001 '**' 0.01 '*' 0.05 '.' 0.1 ' ' 1
## 
## ####################### Model Fit Indices ###########################
##         chisq.scaled df.scaled pvalue.scaled rmsea.scaled cfi.scaled tli.scaled
## narmodc     448.112†        56          .000        .036         NA         NA 
## narmodw     732.202         77          .000        .039         NA         NA 
## narmods     487.855         91          .000        .028†        NA         NA 
##          srmr          aic          bic
## narmodc .024† 1212986.866† 1214376.303†
## narmodw .035  1213465.996  1214673.069 
## narmods .047  1215089.972  1216175.470 
## 
## ################## Differences in Fit Indices #######################
##                   df.scaled rmsea.scaled cfi.scaled tli.scaled  srmr      aic
## narmodw - narmodc        21        0.004         NA         NA 0.012  479.130
## narmods - narmodw        14       -0.011         NA         NA 0.012 1623.976
##                        bic
## narmodw - narmodc  296.766
## narmods - narmodw 1502.401
```

```
fitmeasures(narmodc, fit.measures = c("chisq.scaled", "df.scaled", "cfi.robust", "rmsea.robust", "srmr"))
```

```
## chisq.scaled    df.scaled   cfi.robust rmsea.robust         srmr 
##      448.112       56.000           NA        0.069        0.024
```

```
fitmeasures(narmodw, fit.measures = c("chisq.scaled", "df.scaled", "cfi.robust", "rmsea.robust", "srmr"))
```

```
## chisq.scaled    df.scaled   cfi.robust rmsea.robust         srmr 
##      732.202       77.000           NA        0.069        0.035
```

```
fitmeasures(narmods, fit.measures = c("chisq.scaled", "df.scaled", "cfi.robust", "rmsea.robust", "srmr"))
```

```
## chisq.scaled    df.scaled   cfi.robust rmsea.robust         srmr 
##      487.855       91.000           NA        0.079        0.047
```

```
narcissism <- bind_rows(estimate_lavaan_ten_berge_per_group(narmods))
admire <- narcissism$scores[1]
rival <- narcissism$scores[2]
```

### Moral identity

```
pls2$moralid4 <- 10-pls2$moralid4
pls2$moralid7 <- 10-pls2$moralid7

mid <- 'inter =~ moralid2 + moralid10 + moralid1 + moralid4 + moralid7 
        symbol =~ moralid3 + moralid5 + moralid6 + moralid8 + moralid9'
midmod <- cfa(mid, estimator = "MLR", data = pls2, std.lv = T, cluster = "country")
fitmeasures(midmod, fit.measures = c("chisq.scaled", "df.scaled", "cfi.robust", "rmsea.ci.upper.robust", "srmr"))
```

```
##          chisq.scaled             df.scaled            cfi.robust 
##              4316.377                34.000                 0.854 
## rmsea.ci.upper.robust                  srmr 
##                 0.123                 0.082
```

```
miobj <- modindices(midmod)
top_n(miobj, 3, mi)
```

```
##        lhs op      rhs       mi    epc sepc.lv sepc.all sepc.nox
## 1   symbol =~ moralid4 3876.601 -0.861  -0.861   -0.361   -0.361
## 2 moralid4 ~~ moralid7 4935.676  2.065   2.065    0.352    0.352
## 3 moralid8 ~~ moralid9 8050.674  2.770   2.770    0.617    0.617
```

```
mid <- 'inter =~ moralid2 + moralid10 + moralid1 + moralid4 + moralid7 
        symbol =~ moralid3 + moralid5 + moralid6 + moralid8 + moralid9
        moralid8 ~~ moralid9'
midmod <- cfa(mid, estimator = "MLR", data = pls2, std.lv = T, cluster = "country")
fitmeasures(midmod, fit.measures = c("chisq.scaled", "df.scaled", "cfi.robust", "rmsea.ci.upper.robust", "srmr"))
```

```
##          chisq.scaled             df.scaled            cfi.robust 
##              2675.837                33.000                 0.904 
## rmsea.ci.upper.robust                  srmr 
##                 0.102                 0.077
```

```
miobj <- modindices(midmod)
top_n(miobj, 3, mi)
```

```
##        lhs op      rhs       mi    epc sepc.lv sepc.all sepc.nox
## 1   symbol =~ moralid2 1868.496  0.441   0.441    0.217    0.217
## 2   symbol =~ moralid4 3626.240 -0.859  -0.859   -0.360   -0.360
## 3 moralid4 ~~ moralid7 4941.271  2.066   2.066    0.352    0.352
```

```
mid <- 'inter =~ moralid2 + moralid10 + moralid1 + moralid4 + moralid7 
        symbol =~ moralid3 + moralid5 + moralid6 + moralid8 + moralid9
        moralid8 ~~ moralid9
        moralid4 ~~ moralid7'
midmod <- cfa(mid, estimator = "MLR", data = pls2, std.lv = T, cluster = "country")
fitmeasures(midmod, fit.measures = c("chisq.scaled", "df.scaled", "cfi.robust", "rmsea.ci.upper.robust", "srmr"))
```

```
##          chisq.scaled             df.scaled            cfi.robust 
##              1648.005                32.000                 0.939 
## rmsea.ci.upper.robust                  srmr 
##                 0.084                 0.067
```

```
semPaths(midmod, what = "std", edge.label.cex = 1.2, label.cex = 1.2)
```

```
reliability(midmod)
```

```
##            inter    symbol
## alpha  0.7491873 0.8085402
## omega  0.6799495 0.7504470
## omega2 0.6799495 0.7504470
## omega3 0.6735642 0.7466687
## avevar 0.3528674 0.4253743
```

```
midmodc <- cfa(mid, estimator = "MLR", data = pls2, std.lv = T, cluster = "country", group = "cgr")
```

```
## Warning in lav_model_vcov(lavmodel = lavmodel, lavsamplestats = lavsamplestats, : lavaan WARNING:
##     The variance-covariance matrix of the estimated parameters (vcov)
##     does not appear to be positive definite! The smallest eigenvalue
##     (= -1.699690e-15) is smaller than zero. This may be a symptom that
##     the model is not identified.
```

```
midmodw <- cfa(mid, estimator = "MLR", data = pls2, std.lv = T, cluster = "country", group = "cgr", group.equal = "loadings")
```

```
## Warning in lav_model_vcov(lavmodel = lavmodel, lavsamplestats = lavsamplestats, : lavaan WARNING:
##     The variance-covariance matrix of the estimated parameters (vcov)
##     does not appear to be positive definite! The smallest eigenvalue
##     (= -1.015657e-15) is smaller than zero. This may be a symptom that
##     the model is not identified.
```

```
midmods <- cfa(mid, estimator = "MLR", data = pls2, std.lv = T, cluster = "country", group = "cgr", group.equal = c("loadings", "intercepts"))
```

```
## Warning in lav_model_vcov(lavmodel = lavmodel, lavsamplestats = lavsamplestats, : lavaan WARNING:
##     The variance-covariance matrix of the estimated parameters (vcov)
##     does not appear to be positive definite! The smallest eigenvalue
##     (= 4.066101e-15) is close to zero. This may be a symptom that the
##     model is not identified.
```

```
summary(compareFit(midmodc, midmodw, midmods, nested = T))
```

```
## ################### Nested Model Comparison #########################
## Scaled Chi-Squared Difference Test (method = "satorra.bentler.2001")
## 
## lavaan NOTE:
##     The "Chisq" column contains standard test statistics, not the
##     robust test that should be reported per model. A robust difference
##     test is a function of two standard (not robust) statistics.
##  
##          Df     AIC     BIC Chisq Chisq diff Df diff Pr(>Chisq)    
## midmodc 256 1911593 1913886 10483                                  
## midmodw 312 1912324 1914130 11326     136.49      56  1.139e-08 ***
## midmods 368 1915610 1916930 14723     144.54      56  9.260e-10 ***
## ---
## Signif. codes:  0 '***' 0.001 '**' 0.01 '*' 0.05 '.' 0.1 ' ' 1
## 
## ####################### Model Fit Indices ###########################
##         chisq.scaled df.scaled pvalue.scaled rmsea.robust cfi.robust tli.robust
## midmodc    3481.351        256          .000        .083       .933†      .905 
## midmodw    3164.414        312          .000        .077†      .929       .918†
## midmods    2226.889†       368          .000        .078       .915       .917 
##          srmr          aic          bic
## midmodc .068† 1911593.174† 1913885.745†
## midmodw .074  1912324.074  1914130.342 
## midmods .080  1915609.760  1916929.725 
## 
## ################## Differences in Fit Indices #######################
##                   df.scaled rmsea.robust cfi.robust tli.robust  srmr      aic
## midmodw - midmodc        56       -0.006     -0.003      0.013 0.006  730.900
## midmods - midmodw        56        0.001     -0.014     -0.002 0.006 3285.685
##                        bic
## midmodw - midmodc  244.597
## midmods - midmodw 2799.382
```

```
moralids <- bind_rows(estimate_lavaan_ten_berge_per_group(midmods))
internal <- moralids$scores[1]
symbol <- moralids$scores[2]
```

## Two-item measures

Invariance could not be tested with two-item measures in the same way as it is tested in multi-item models. Therefore, our best approximation was to conduct exploratory factor analysis (principal axis factoring) and extract obtained factor scores. Prior to their extraction, we checked whether the correlations of our variables allow extraction of meaningful factors, which was confirmed as values were moderate to high.

```
cor(pls2[, 59:60])
```

```
##            nidentity1 nidentity2
## nidentity1   1.000000   0.688286
## nidentity2   0.688286   1.000000
```

```
cor(pls2[, 75:76])
```

```
##           riskperc1 riskperc2
## riskperc1 1.0000000 0.6642859
## riskperc2 0.6642859 1.0000000
```

```
cor(pls2[, 67:68])
```

```
##           optim1    optim2
## optim1 1.0000000 0.7092559
## optim2 0.7092559 1.0000000
```

```
risk <- fa(pls2[, 75:76], nfactors = 1, fm = "pa")$scores
natid <- fa(pls2[, 59:60], nfactors = 1, fm = "pa")$scores
optim <- fa(pls2[, 67:68], nfactors = 1, fm = "pa")$scores
```

### Single-item measures

Single-item measures were retained in their original form, except in the ase of CRT, urbanization, marital status and employment, which were recoded due to their categorical nature.

```
selfesteem <- pls2$self_esteem
ladders <- pls2$ladder
mor_circles <- pls2$mor_circle
healths <- pls2$health_cond
polid <- pls2$political_ideology
pls2$CRT1 <- ifelse(pls2$CRT1 == 1, 1, 0)
pls2$CRT2 <- ifelse(pls2$CRT2 == 1, 1, 0)
pls2$CRT3 <- ifelse(pls2$CRT3 == 1, 1, 0)
ccrt <- rowSums(pls2[, c("CRT1", "CRT2", "CRT3")])
test_pos <- pls2$tested_positive
know_pos <- pls2$know_tested_positive
urban <- ifelse(pls2$urban == 1, 1, 0) #1-urban
marital <- ifelse(pls2$marital1 == 1, 0, 1) #1- married or in a relationship
employment <- ifelse(pls2$employ_status1 %in% c(1,2,4,5), 1, 0) #1-employed, student or retired
age <- pls2$age
children <- pls2$children
sex <- pls2$sex1
country <- pls2$country
cgr <- pls2$cgr

#### finalizing ####

complete_finalpc <- data.frame(country, cgr, physicals$scores, hygienes$scores, policys$scores, collectivenar,
                             conspiracy, ominds$scores, mcoops$scores, belongs$scores, selfcontrols$scores, 
                             admire, rival, internal, symbol, optim, risk, natid, 
                             selfesteem, ladders, mor_circles, healths, polid, ccrt,
                             test_pos, know_pos, urban, marital, employment, age, children, 
                             sex)
names(complete_finalpc)[c(3,4,5,8,9,10,11,16,17,18)] <- c("phc", "phg", "phs", "om", "mcoop", "belong", "selfcontrol", "optim", "risk", "natid")
```

```
write.csv(complete_finalpc, "cfpc.csv")
```

These data were used in the main analyses, presented in the following sections.

# Random forests

These analyses were originally prepared as a separate script. Therefore, the entire process starts from scratch (i.e., from activation of packages).

## Activating libraries

```
sapply(c("readr", "tidyr", "dplyr", "ggplot2", "ranger"), library, character.only = T)
```

## Preparing trainng and test sets

The following codes were used to prepare the training and test sets. The algorithm randomly assigned 20% of each national sample to test set, which was later used to evaluate R2 and variable importance.

```
cf <- read_csv("cfpc.csv")
```

```
## New names:
## * `` -> ...1
```

```
## Rows: 43651 Columns: 34
```

```
## -- Column specification --------------------------------------------------------
## Delimiter: ","
## chr  (2): country, cgr
## dbl (32): ...1, phc, phg, phs, coln, cons, om, mcoop, belong, selfcontrol, a...
```

```
## 
## i Use `spec()` to retrieve the full column specification for this data.
## i Specify the column types or set `show_col_types = FALSE` to quiet this message.
```

```
sf <- cf[, 2:33]
sf2 <- split.data.frame(sf, sf$country)

outlist <- list()   

for(i in seq_along(sf2)){
  dff <- sf2[[i]]
  set.seed(i)
  mn <- sample.int(nrow(dff), round(.8*nrow(dff), 0))
  dff$w8 <- 0
  dff[mn, "w8"] <- 1
  outlist[[i]] <- dff
}

sf2 <- bind_rows(outlist)
```

## Search for optimal hyperparameters

The quantity of data prevented us from preparing a single script for the entire analysis. The following codes were used to conduct analyses on Isabella cluster and are *not evaluated* in this document.

```
sf3 <- subset(sf2, sf2$cgr == "AFIS")

olss <- list()
outlosst <- list()
for(i in seq(3, 100, 3)){
  outdff <- data.frame(model = character(), r2 = numeric())
  for(j in seq(5, 20, 1)){
    outdff <- data.frame(model = character(), r2 = numeric())
    for(k in c(1000, 2000)){
      rwt <- ranger(phc~.-country-cgr-w8-phs-phg, data = sf3, num.trees = k, importance = "permutation", num.threads = as.integer(Sys.getenv("NSLOTS")), mtry = j, min.node.size = i, seed = 362, case.weights = sf2$w8, holdout = T, splitrule = "variance")
      outdff[k/1000, 1] <- paste0("trees", k, "_mtry", j, "_mns", i)
      outdff[k/1000, 2] <- rwt$r.squared
    }
    outlosst[[j]] <- outdff
  }
  olss[[i]] <- bind_rows(outlosst)
}

outrf_contact <- bind_rows(olss)
write.csv(outrf_contact, "afis_outrf_contact_ori.csv")


olss <- list()
outlosst <- list()
for(i in seq(3, 100, 3)){
  outdff <- data.frame(model = character(), r2 = numeric())
  for(j in seq(5, 20, 1)){
    outdff <- data.frame(model = character(), r2 = numeric())
    for(k in c(1000, 2000)){
      rwt <- ranger(phg~.-country-cgr-w8-phc-phs, data = sf3, num.trees = k, importance = "permutation", num.threads = as.integer(Sys.getenv("NSLOTS")), mtry = j, min.node.size = i, seed = 362, case.weights = sf2$w8, holdout = T, splitrule = "variance")
      outdff[k/1000, 1] <- paste0("trees", k, "_mtry", j, "_mns", i)
      outdff[k/1000, 2] <- rwt$r.squared
    }
    outlosst[[j]] <- outdff
  }
  olss[[i]] <- bind_rows(outlosst)
}

outrf_hygiene <- bind_rows(olss)
write.csv(outrf_hygiene, "afis_outrf_hygiene_ori.csv")

olss <- list()
outlosst <- list()
for(i in seq(3, 100, 3)){
  outdff <- data.frame(model = character(), r2 = numeric())
  for(j in seq(5, 20, 1)){
    outdff <- data.frame(model = character(), r2 = numeric())
    for(k in c(1000, 2000)){
      rwt <- ranger(phs~.-country-cgr-w8-phg-phc, data = sf3, num.trees = k, importance = "permutation", num.threads = as.integer(Sys.getenv("NSLOTS")), mtry = j, min.node.size = i, seed = 362, case.weights = sf2$w8, holdout = T, splitrule = "variance")
      outdff[k/1000, 1] <- paste0("trees", k, "_mtry", j, "_mns", i)
      outdff[k/1000, 2] <- rwt$r.squared
    }
    outlosst[[j]] <- outdff
  }
  olss[[i]] <- bind_rows(outlosst)
}

outrf_policy <- bind_rows(olss)
write.csv(outrf_policy, "afis_outrf_policy_ori.csv")

# 
sf3 <- subset(sf2, sf2$cgr == "CATEU")

olss <- list()
outlosst <- list()
for(i in seq(3, 100, 3)){
  outdff <- data.frame(model = character(), r2 = numeric())
  for(j in seq(5, 20, 1)){
    outdff <- data.frame(model = character(), r2 = numeric())
    for(k in c(1000, 2000)){
      rwt <- ranger(phc~.-country-cgr-w8-phs-phg, data = sf3, num.trees = k, importance = "permutation", num.threads = as.integer(Sys.getenv("NSLOTS")), mtry = j, min.node.size = i, seed = 362, case.weights = sf2$w8, holdout = T, splitrule = "variance")
      outdff[k/1000, 1] <- paste0("trees", k, "_mtry", j, "_mns", i)
      outdff[k/1000, 2] <- rwt$r.squared
    }
    outlosst[[j]] <- outdff
  }
  olss[[i]] <- bind_rows(outlosst)
}

outrf_contact <- bind_rows(olss)
write.csv(outrf_contact, "cateu_outrf_contact_ori.csv")


olss <- list()
outlosst <- list()
for(i in seq(3, 100, 3)){
  outdff <- data.frame(model = character(), r2 = numeric())
  for(j in seq(5, 20, 1)){
    outdff <- data.frame(model = character(), r2 = numeric())
    for(k in c(1000, 2000)){
      rwt <- ranger(phg~.-country-cgr-w8-phc-phs, data = sf3, num.trees = k, importance = "permutation", num.threads = as.integer(Sys.getenv("NSLOTS")), mtry = j, min.node.size = i, seed = 362, case.weights = sf2$w8, holdout = T, splitrule = "variance")
      outdff[k/1000, 1] <- paste0("trees", k, "_mtry", j, "_mns", i)
      outdff[k/1000, 2] <- rwt$r.squared
    }
    outlosst[[j]] <- outdff
  }
  olss[[i]] <- bind_rows(outlosst)
}

outrf_hygiene <- bind_rows(olss)
write.csv(outrf_hygiene, "cateu_outrf_hygiene_ori.csv")

olss <- list()
outlosst <- list()
for(i in seq(3, 100, 3)){
  outdff <- data.frame(model = character(), r2 = numeric())
  for(j in seq(5, 20, 1)){
    outdff <- data.frame(model = character(), r2 = numeric())
    for(k in c(1000, 2000)){
      rwt <- ranger(phs~.-country-cgr-w8-phg-phc, data = sf3, num.trees = k, importance = "permutation", num.threads = as.integer(Sys.getenv("NSLOTS")), mtry = j, min.node.size = i, seed = 362, case.weights = sf2$w8, holdout = T, splitrule = "variance")
      outdff[k/1000, 1] <- paste0("trees", k, "_mtry", j, "_mns", i)
      outdff[k/1000, 2] <- rwt$r.squared
    }
    outlosst[[j]] <- outdff
  }
  olss[[i]] <- bind_rows(outlosst)
}

outrf_policy <- bind_rows(olss)
write.csv(outrf_policy, "cateu_outrf_policy_ori.csv")


# 
sf3 <- subset(sf2, sf2$cgr == "CONF")

olss <- list()
outlosst <- list()
for(i in seq(3, 100, 3)){
  outdff <- data.frame(model = character(), r2 = numeric())
  for(j in seq(5, 20, 1)){
    outdff <- data.frame(model = character(), r2 = numeric())
    for(k in c(1000, 2000)){
      rwt <- ranger(phc~.-country-cgr-w8-phs-phg, data = sf3, num.trees = k, importance = "permutation", num.threads = as.integer(Sys.getenv("NSLOTS")), mtry = j, min.node.size = i, seed = 362, case.weights = sf2$w8, holdout = T, splitrule = "variance")
      outdff[k/1000, 1] <- paste0("trees", k, "_mtry", j, "_mns", i)
      outdff[k/1000, 2] <- rwt$r.squared
    }
    outlosst[[j]] <- outdff
  }
  olss[[i]] <- bind_rows(outlosst)
}

outrf_contact <- bind_rows(olss)
write.csv(outrf_contact, "conf_outrf_contact_ori.csv")


olss <- list()
outlosst <- list()
for(i in seq(3, 100, 3)){
  outdff <- data.frame(model = character(), r2 = numeric())
  for(j in seq(5, 20, 1)){
    outdff <- data.frame(model = character(), r2 = numeric())
    for(k in c(1000, 2000)){
      rwt <- ranger(phg~.-country-cgr-w8-phc-phs, data = sf3, num.trees = k, importance = "permutation", num.threads = as.integer(Sys.getenv("NSLOTS")), mtry = j, min.node.size = i, seed = 362, case.weights = sf2$w8, holdout = T, splitrule = "variance")
      outdff[k/1000, 1] <- paste0("trees", k, "_mtry", j, "_mns", i)
      outdff[k/1000, 2] <- rwt$r.squared
    }
    outlosst[[j]] <- outdff
  }
  olss[[i]] <- bind_rows(outlosst)
}

outrf_hygiene <- bind_rows(olss)
write.csv(outrf_hygiene, "conf_outrf_hygiene_ori.csv")

olss <- list()
outlosst <- list()
for(i in seq(3, 100, 3)){
  outdff <- data.frame(model = character(), r2 = numeric())
  for(j in seq(5, 20, 1)){
    outdff <- data.frame(model = character(), r2 = numeric())
    for(k in c(1000, 2000)){
      rwt <- ranger(phs~.-country-cgr-w8-phg-phc, data = sf3, num.trees = k, importance = "permutation", num.threads = as.integer(Sys.getenv("NSLOTS")), mtry = j, min.node.size = i, seed = 362, case.weights = sf2$w8, holdout = T, splitrule = "variance")
      outdff[k/1000, 1] <- paste0("trees", k, "_mtry", j, "_mns", i)
      outdff[k/1000, 2] <- rwt$r.squared
    }
    outlosst[[j]] <- outdff
  }
  olss[[i]] <- bind_rows(outlosst)
}

outrf_policy <- bind_rows(olss)
write.csv(outrf_policy, "conf_outrf_policy_ori.csv")


# 
sf3 <- subset(sf2, sf2$cgr == "ES")

olss <- list()
outlosst <- list()
for(i in seq(3, 100, 3)){
  outdff <- data.frame(model = character(), r2 = numeric())
  for(j in seq(5, 20, 1)){
    outdff <- data.frame(model = character(), r2 = numeric())
    for(k in c(1000, 2000)){
      rwt <- ranger(phc~.-country-cgr-w8-phs-phg, data = sf3, num.trees = k, importance = "permutation", num.threads = as.integer(Sys.getenv("NSLOTS")), mtry = j, min.node.size = i, seed = 362, case.weights = sf2$w8, holdout = T, splitrule = "variance")
      outdff[k/1000, 1] <- paste0("trees", k, "_mtry", j, "_mns", i)
      outdff[k/1000, 2] <- rwt$r.squared
    }
    outlosst[[j]] <- outdff
  }
  olss[[i]] <- bind_rows(outlosst)
}

outrf_contact <- bind_rows(olss)
write.csv(outrf_contact, "es_outrf_contact_ori.csv")


olss <- list()
outlosst <- list()
for(i in seq(3, 100, 3)){
  outdff <- data.frame(model = character(), r2 = numeric())
  for(j in seq(5, 20, 1)){
    outdff <- data.frame(model = character(), r2 = numeric())
    for(k in c(1000, 2000)){
      rwt <- ranger(phg~.-country-cgr-w8-phc-phs, data = sf3, num.trees = k, importance = "permutation", num.threads = as.integer(Sys.getenv("NSLOTS")), mtry = j, min.node.size = i, seed = 362, case.weights = sf2$w8, holdout = T, splitrule = "variance")
      outdff[k/1000, 1] <- paste0("trees", k, "_mtry", j, "_mns", i)
      outdff[k/1000, 2] <- rwt$r.squared
    }
    outlosst[[j]] <- outdff
  }
  olss[[i]] <- bind_rows(outlosst)
}

outrf_hygiene <- bind_rows(olss)
write.csv(outrf_hygiene, "es_outrf_hygiene_ori.csv")

olss <- list()
outlosst <- list()
for(i in seq(3, 100, 3)){
  outdff <- data.frame(model = character(), r2 = numeric())
  for(j in seq(5, 20, 1)){
    outdff <- data.frame(model = character(), r2 = numeric())
    for(k in c(1000, 2000)){
      rwt <- ranger(phs~.-country-cgr-w8-phg-phc, data = sf3, num.trees = k, importance = "permutation", num.threads = as.integer(Sys.getenv("NSLOTS")), mtry = j, min.node.size = i, seed = 362, case.weights = sf2$w8, holdout = T, splitrule = "variance")
      outdff[k/1000, 1] <- paste0("trees", k, "_mtry", j, "_mns", i)
      outdff[k/1000, 2] <- rwt$r.squared
    }
    outlosst[[j]] <- outdff
  }
  olss[[i]] <- bind_rows(outlosst)
}

outrf_policy <- bind_rows(olss)
write.csv(outrf_policy, "es_outrf_policy_ori.csv")


# 
sf3 <- subset(sf2, sf2$cgr == "LATAM")

olss <- list()
outlosst <- list()
for(i in seq(3, 100, 3)){
  outdff <- data.frame(model = character(), r2 = numeric())
  for(j in seq(5, 20, 1)){
    outdff <- data.frame(model = character(), r2 = numeric())
    for(k in c(1000, 2000)){
      rwt <- ranger(phc~.-country-cgr-w8-phs-phg, data = sf3, num.trees = k, importance = "permutation", num.threads = as.integer(Sys.getenv("NSLOTS")), mtry = j, min.node.size = i, seed = 362, case.weights = sf2$w8, holdout = T, splitrule = "variance")
      outdff[k/1000, 1] <- paste0("trees", k, "_mtry", j, "_mns", i)
      outdff[k/1000, 2] <- rwt$r.squared
    }
    outlosst[[j]] <- outdff
  }
  olss[[i]] <- bind_rows(outlosst)
}

outrf_contact <- bind_rows(olss)
write.csv(outrf_contact, "latam_outrf_contact_ori.csv")


olss <- list()
outlosst <- list()
for(i in seq(3, 100, 3)){
  outdff <- data.frame(model = character(), r2 = numeric())
  for(j in seq(5, 20, 1)){
    outdff <- data.frame(model = character(), r2 = numeric())
    for(k in c(1000, 2000)){
      rwt <- ranger(phg~.-country-cgr-w8-phc-phs, data = sf3, num.trees = k, importance = "permutation", num.threads = as.integer(Sys.getenv("NSLOTS")), mtry = j, min.node.size = i, seed = 362, case.weights = sf2$w8, holdout = T, splitrule = "variance")
      outdff[k/1000, 1] <- paste0("trees", k, "_mtry", j, "_mns", i)
      outdff[k/1000, 2] <- rwt$r.squared
    }
    outlosst[[j]] <- outdff
  }
  olss[[i]] <- bind_rows(outlosst)
}

outrf_hygiene <- bind_rows(olss)
write.csv(outrf_hygiene, "latam_outrf_hygiene_ori.csv")

olss <- list()
outlosst <- list()
for(i in seq(3, 100, 3)){
  outdff <- data.frame(model = character(), r2 = numeric())
  for(j in seq(5, 20, 1)){
    outdff <- data.frame(model = character(), r2 = numeric())
    for(k in c(1000, 2000)){
      rwt <- ranger(phs~.-country-cgr-w8-phg-phc, data = sf3, num.trees = k, importance = "permutation", num.threads = as.integer(Sys.getenv("NSLOTS")), mtry = j, min.node.size = i, seed = 362, case.weights = sf2$w8, holdout = T, splitrule = "variance")
      outdff[k/1000, 1] <- paste0("trees", k, "_mtry", j, "_mns", i)
      outdff[k/1000, 2] <- rwt$r.squared
    }
    outlosst[[j]] <- outdff
  }
  olss[[i]] <- bind_rows(outlosst)
}

outrf_policy <- bind_rows(olss)
write.csv(outrf_policy, "latam_outrf_policy_ori.csv")


# 
sf3 <- subset(sf2, sf2$cgr == "ORT")

olss <- list()
outlosst <- list()
for(i in seq(3, 100, 3)){
  outdff <- data.frame(model = character(), r2 = numeric())
  for(j in seq(5, 20, 1)){
    outdff <- data.frame(model = character(), r2 = numeric())
    for(k in c(1000, 2000)){
      rwt <- ranger(phc~.-country-cgr-w8-phs-phg, data = sf3, num.trees = k, importance = "permutation", num.threads = as.integer(Sys.getenv("NSLOTS")), mtry = j, min.node.size = i, seed = 362, case.weights = sf2$w8, holdout = T, splitrule = "variance")
      outdff[k/1000, 1] <- paste0("trees", k, "_mtry", j, "_mns", i)
      outdff[k/1000, 2] <- rwt$r.squared
    }
    outlosst[[j]] <- outdff
  }
  olss[[i]] <- bind_rows(outlosst)
}

outrf_contact <- bind_rows(olss)
write.csv(outrf_contact, "ort_outrf_contact_ori.csv")


olss <- list()
outlosst <- list()
for(i in seq(3, 100, 3)){
  outdff <- data.frame(model = character(), r2 = numeric())
  for(j in seq(5, 20, 1)){
    outdff <- data.frame(model = character(), r2 = numeric())
    for(k in c(1000, 2000)){
      rwt <- ranger(phg~.-country-cgr-w8-phc-phs, data = sf3, num.trees = k, importance = "permutation", num.threads = as.integer(Sys.getenv("NSLOTS")), mtry = j, min.node.size = i, seed = 362, case.weights = sf2$w8, holdout = T, splitrule = "variance")
      outdff[k/1000, 1] <- paste0("trees", k, "_mtry", j, "_mns", i)
      outdff[k/1000, 2] <- rwt$r.squared
    }
    outlosst[[j]] <- outdff
  }
  olss[[i]] <- bind_rows(outlosst)
}

outrf_hygiene <- bind_rows(olss)
write.csv(outrf_hygiene, "ort_outrf_hygiene_ori.csv")

olss <- list()
outlosst <- list()
for(i in seq(3, 100, 3)){
  outdff <- data.frame(model = character(), r2 = numeric())
  for(j in seq(5, 20, 1)){
    outdff <- data.frame(model = character(), r2 = numeric())
    for(k in c(1000, 2000)){
      rwt <- ranger(phs~.-country-cgr-w8-phg-phc, data = sf3, num.trees = k, importance = "permutation", num.threads = as.integer(Sys.getenv("NSLOTS")), mtry = j, min.node.size = i, seed = 362, case.weights = sf2$w8, holdout = T, splitrule = "variance")
      outdff[k/1000, 1] <- paste0("trees", k, "_mtry", j, "_mns", i)
      outdff[k/1000, 2] <- rwt$r.squared
    }
    outlosst[[j]] <- outdff
  }
  olss[[i]] <- bind_rows(outlosst)
}

outrf_policy <- bind_rows(olss)
write.csv(outrf_policy, "ort_outrf_policy_ori.csv")


# 
sf3 <- subset(sf2, sf2$cgr == "PROTEU")

olss <- list()
outlosst <- list()
for(i in seq(3, 100, 3)){
  outdff <- data.frame(model = character(), r2 = numeric())
  for(j in seq(5, 20, 1)){
    outdff <- data.frame(model = character(), r2 = numeric())
    for(k in c(1000, 2000)){
      rwt <- ranger(phc~.-country-cgr-w8-phs-phg, data = sf3, num.trees = k, importance = "permutation", num.threads = as.integer(Sys.getenv("NSLOTS")), mtry = j, min.node.size = i, seed = 362, case.weights = sf2$w8, holdout = T, splitrule = "variance")
      outdff[k/1000, 1] <- paste0("trees", k, "_mtry", j, "_mns", i)
      outdff[k/1000, 2] <- rwt$r.squared
    }
    outlosst[[j]] <- outdff
  }
  olss[[i]] <- bind_rows(outlosst)
}

outrf_contact <- bind_rows(olss)
write.csv(outrf_contact, "proteu_outrf_contact_ori.csv")


olss <- list()
outlosst <- list()
for(i in seq(3, 100, 3)){
  outdff <- data.frame(model = character(), r2 = numeric())
  for(j in seq(5, 20, 1)){
    outdff <- data.frame(model = character(), r2 = numeric())
    for(k in c(1000, 2000)){
      rwt <- ranger(phg~.-country-cgr-w8-phc-phs, data = sf3, num.trees = k, importance = "permutation", num.threads = as.integer(Sys.getenv("NSLOTS")), mtry = j, min.node.size = i, seed = 362, case.weights = sf2$w8, holdout = T, splitrule = "variance")
      outdff[k/1000, 1] <- paste0("trees", k, "_mtry", j, "_mns", i)
      outdff[k/1000, 2] <- rwt$r.squared
    }
    outlosst[[j]] <- outdff
  }
  olss[[i]] <- bind_rows(outlosst)
}

outrf_hygiene <- bind_rows(olss)
write.csv(outrf_hygiene, "proteu_outrf_hygiene_ori.csv")

olss <- list()
outlosst <- list()
for(i in seq(3, 100, 3)){
  outdff <- data.frame(model = character(), r2 = numeric())
  for(j in seq(5, 20, 1)){
    outdff <- data.frame(model = character(), r2 = numeric())
    for(k in c(1000, 2000)){
      rwt <- ranger(phs~.-country-cgr-w8-phg-phc, data = sf3, num.trees = k, importance = "permutation", num.threads = as.integer(Sys.getenv("NSLOTS")), mtry = j, min.node.size = i, seed = 362, case.weights = sf2$w8, holdout = T, splitrule = "variance")
      outdff[k/1000, 1] <- paste0("trees", k, "_mtry", j, "_mns", i)
      outdff[k/1000, 2] <- rwt$r.squared
    }
    outlosst[[j]] <- outdff
  }
  olss[[i]] <- bind_rows(outlosst)
}

outrf_policy <- bind_rows(olss)
write.csv(outrf_policy, "proteu_outrf_policy_ori.csv")


# 
sf3 <- subset(sf2, sf2$cgr == "SA")

olss <- list()
outlosst <- list()
for(i in seq(3, 100, 3)){
  outdff <- data.frame(model = character(), r2 = numeric())
  for(j in seq(5, 20, 1)){
    outdff <- data.frame(model = character(), r2 = numeric())
    for(k in c(1000, 2000)){
      rwt <- ranger(phc~.-country-cgr-w8-phs-phg, data = sf3, num.trees = k, importance = "permutation", num.threads = as.integer(Sys.getenv("NSLOTS")), mtry = j, min.node.size = i, seed = 362, case.weights = sf2$w8, holdout = T, splitrule = "variance")
      outdff[k/1000, 1] <- paste0("trees", k, "_mtry", j, "_mns", i)
      outdff[k/1000, 2] <- rwt$r.squared
    }
    outlosst[[j]] <- outdff
  }
  olss[[i]] <- bind_rows(outlosst)
}

outrf_contact <- bind_rows(olss)
write.csv(outrf_contact, "sa_outrf_contact_ori.csv")


olss <- list()
outlosst <- list()
for(i in seq(3, 100, 3)){
  outdff <- data.frame(model = character(), r2 = numeric())
  for(j in seq(5, 20, 1)){
    outdff <- data.frame(model = character(), r2 = numeric())
    for(k in c(1000, 2000)){
      rwt <- ranger(phg~.-country-cgr-w8-phc-phs, data = sf3, num.trees = k, importance = "permutation", num.threads = as.integer(Sys.getenv("NSLOTS")), mtry = j, min.node.size = i, seed = 362, case.weights = sf2$w8, holdout = T, splitrule = "variance")
      outdff[k/1000, 1] <- paste0("trees", k, "_mtry", j, "_mns", i)
      outdff[k/1000, 2] <- rwt$r.squared
    }
    outlosst[[j]] <- outdff
  }
  olss[[i]] <- bind_rows(outlosst)
}

outrf_hygiene <- bind_rows(olss)
write.csv(outrf_hygiene, "sa_outrf_hygiene_ori.csv")

olss <- list()
outlosst <- list()
for(i in seq(3, 100, 3)){
  outdff <- data.frame(model = character(), r2 = numeric())
  for(j in seq(5, 20, 1)){
    outdff <- data.frame(model = character(), r2 = numeric())
    for(k in c(1000, 2000)){
      rwt <- ranger(phs~.-country-cgr-w8-phg-phc, data = sf3, num.trees = k, importance = "permutation", num.threads = as.integer(Sys.getenv("NSLOTS")), mtry = j, min.node.size = i, seed = 362, case.weights = sf2$w8, holdout = T, splitrule = "variance")
      outdff[k/1000, 1] <- paste0("trees", k, "_mtry", j, "_mns", i)
      outdff[k/1000, 2] <- rwt$r.squared
    }
    outlosst[[j]] <- outdff
  }
  olss[[i]] <- bind_rows(outlosst)
}

outrf_policy <- bind_rows(olss)
write.csv(outrf_policy, "sa_outrf_policy_ori.csv")
```

### Output

As the analyses were prepared elsewhere, the following lines of code read in the results which are relevant in choosing the optimal model.

```
afis_p <- read_csv("afis_outrf_policy_ori.csv")
```

```
## New names:
## * `` -> ...1
```

```
## Rows: 1056 Columns: 3
```

```
## -- Column specification --------------------------------------------------------
## Delimiter: ","
## chr (1): model
## dbl (2): ...1, r2
```

```
## 
## i Use `spec()` to retrieve the full column specification for this data.
## i Specify the column types or set `show_col_types = FALSE` to quiet this message.
```

```
afis_h <- read_csv("afis_outrf_hygiene_ori.csv")
```

```
## New names:
## * `` -> ...1
```

```
## Rows: 1056 Columns: 3
```

```
## -- Column specification --------------------------------------------------------
## Delimiter: ","
## chr (1): model
## dbl (2): ...1, r2
```

```
## 
## i Use `spec()` to retrieve the full column specification for this data.
## i Specify the column types or set `show_col_types = FALSE` to quiet this message.
```

```
afis_c <- read_csv("afis_outrf_contact_ori.csv")
```

```
## New names:
## * `` -> ...1
```

```
## Rows: 1056 Columns: 3
```

```
## -- Column specification --------------------------------------------------------
## Delimiter: ","
## chr (1): model
## dbl (2): ...1, r2
```

```
## 
## i Use `spec()` to retrieve the full column specification for this data.
## i Specify the column types or set `show_col_types = FALSE` to quiet this message.
```

```
sa_p <- read_csv("sa_outrf_policy_ori.csv")
```

```
## New names:
## * `` -> ...1
```

```
## Rows: 1056 Columns: 3
```

```
## -- Column specification --------------------------------------------------------
## Delimiter: ","
## chr (1): model
## dbl (2): ...1, r2
```

```
## 
## i Use `spec()` to retrieve the full column specification for this data.
## i Specify the column types or set `show_col_types = FALSE` to quiet this message.
```

```
sa_h <- read_csv("sa_outrf_hygiene_ori.csv")
```

```
## New names:
## * `` -> ...1
```

```
## Rows: 1056 Columns: 3
```

```
## -- Column specification --------------------------------------------------------
## Delimiter: ","
## chr (1): model
## dbl (2): ...1, r2
```

```
## 
## i Use `spec()` to retrieve the full column specification for this data.
## i Specify the column types or set `show_col_types = FALSE` to quiet this message.
```

```
sa_c <- read_csv("sa_outrf_contact_ori.csv")
```

```
## New names:
## * `` -> ...1
```

```
## Rows: 1056 Columns: 3
```

```
## -- Column specification --------------------------------------------------------
## Delimiter: ","
## chr (1): model
## dbl (2): ...1, r2
```

```
## 
## i Use `spec()` to retrieve the full column specification for this data.
## i Specify the column types or set `show_col_types = FALSE` to quiet this message.
```

```
cateu_p <- read_csv("cateu_outrf_policy_ori.csv")
```

```
## New names:
## * `` -> ...1
```

```
## Rows: 1056 Columns: 3
```

```
## -- Column specification --------------------------------------------------------
## Delimiter: ","
## chr (1): model
## dbl (2): ...1, r2
```

```
## 
## i Use `spec()` to retrieve the full column specification for this data.
## i Specify the column types or set `show_col_types = FALSE` to quiet this message.
```

```
cateu_h <- read_csv("cateu_outrf_hygiene_ori.csv")
```

```
## New names:
## * `` -> ...1
```

```
## Rows: 1056 Columns: 3
```

```
## -- Column specification --------------------------------------------------------
## Delimiter: ","
## chr (1): model
## dbl (2): ...1, r2
```

```
## 
## i Use `spec()` to retrieve the full column specification for this data.
## i Specify the column types or set `show_col_types = FALSE` to quiet this message.
```

```
cateu_c <- read_csv("cateu_outrf_contact_ori.csv")
```

```
## New names:
## * `` -> ...1
```

```
## Rows: 1056 Columns: 3
```

```
## -- Column specification --------------------------------------------------------
## Delimiter: ","
## chr (1): model
## dbl (2): ...1, r2
```

```
## 
## i Use `spec()` to retrieve the full column specification for this data.
## i Specify the column types or set `show_col_types = FALSE` to quiet this message.
```

```
conf_p <- read_csv("conf_outrf_policy_ori.csv")
```

```
## New names:
## * `` -> ...1
```

```
## Rows: 1056 Columns: 3
```

```
## -- Column specification --------------------------------------------------------
## Delimiter: ","
## chr (1): model
## dbl (2): ...1, r2
```

```
## 
## i Use `spec()` to retrieve the full column specification for this data.
## i Specify the column types or set `show_col_types = FALSE` to quiet this message.
```

```
conf_h <- read_csv("conf_outrf_hygiene_ori.csv")
```

```
## New names:
## * `` -> ...1
```

```
## Rows: 1056 Columns: 3
```

```
## -- Column specification --------------------------------------------------------
## Delimiter: ","
## chr (1): model
## dbl (2): ...1, r2
```

```
## 
## i Use `spec()` to retrieve the full column specification for this data.
## i Specify the column types or set `show_col_types = FALSE` to quiet this message.
```

```
conf_c <- read_csv("conf_outrf_contact_ori.csv")
```

```
## New names:
## * `` -> ...1
```

```
## Rows: 1056 Columns: 3
```

```
## -- Column specification --------------------------------------------------------
## Delimiter: ","
## chr (1): model
## dbl (2): ...1, r2
```

```
## 
## i Use `spec()` to retrieve the full column specification for this data.
## i Specify the column types or set `show_col_types = FALSE` to quiet this message.
```

```
es_p <- read_csv("es_outrf_policy_ori.csv")
```

```
## New names:
## * `` -> ...1
```

```
## Rows: 1056 Columns: 3
```

```
## -- Column specification --------------------------------------------------------
## Delimiter: ","
## chr (1): model
## dbl (2): ...1, r2
```

```
## 
## i Use `spec()` to retrieve the full column specification for this data.
## i Specify the column types or set `show_col_types = FALSE` to quiet this message.
```

```
es_h <- read_csv("es_outrf_hygiene_ori.csv")
```

```
## New names:
## * `` -> ...1
```

```
## Rows: 1056 Columns: 3
```

```
## -- Column specification --------------------------------------------------------
## Delimiter: ","
## chr (1): model
## dbl (2): ...1, r2
```

```
## 
## i Use `spec()` to retrieve the full column specification for this data.
## i Specify the column types or set `show_col_types = FALSE` to quiet this message.
```

```
es_c <- read_csv("es_outrf_contact_ori.csv")
```

```
## New names:
## * `` -> ...1
```

```
## Rows: 1056 Columns: 3
```

```
## -- Column specification --------------------------------------------------------
## Delimiter: ","
## chr (1): model
## dbl (2): ...1, r2
```

```
## 
## i Use `spec()` to retrieve the full column specification for this data.
## i Specify the column types or set `show_col_types = FALSE` to quiet this message.
```

```
latam_p <- read_csv("latam_outrf_policy_ori.csv")
```

```
## New names:
## * `` -> ...1
```

```
## Rows: 1056 Columns: 3
```

```
## -- Column specification --------------------------------------------------------
## Delimiter: ","
## chr (1): model
## dbl (2): ...1, r2
```

```
## 
## i Use `spec()` to retrieve the full column specification for this data.
## i Specify the column types or set `show_col_types = FALSE` to quiet this message.
```

```
latam_h <- read_csv("latam_outrf_hygiene_ori.csv")
```

```
## New names:
## * `` -> ...1
```

```
## Rows: 1056 Columns: 3
```

```
## -- Column specification --------------------------------------------------------
## Delimiter: ","
## chr (1): model
## dbl (2): ...1, r2
```

```
## 
## i Use `spec()` to retrieve the full column specification for this data.
## i Specify the column types or set `show_col_types = FALSE` to quiet this message.
```

```
latam_c <- read_csv("latam_outrf_contact_ori.csv")
```

```
## New names:
## * `` -> ...1
```

```
## Rows: 1056 Columns: 3
```

```
## -- Column specification --------------------------------------------------------
## Delimiter: ","
## chr (1): model
## dbl (2): ...1, r2
```

```
## 
## i Use `spec()` to retrieve the full column specification for this data.
## i Specify the column types or set `show_col_types = FALSE` to quiet this message.
```

```
ort_p <- read_csv("ort_outrf_policy_ori.csv")
```

```
## New names:
## * `` -> ...1
```

```
## Rows: 1056 Columns: 3
```

```
## -- Column specification --------------------------------------------------------
## Delimiter: ","
## chr (1): model
## dbl (2): ...1, r2
```

```
## 
## i Use `spec()` to retrieve the full column specification for this data.
## i Specify the column types or set `show_col_types = FALSE` to quiet this message.
```

```
ort_h <- read_csv("ort_outrf_hygiene_ori.csv")
```

```
## New names:
## * `` -> ...1
```

```
## Rows: 1056 Columns: 3
```

```
## -- Column specification --------------------------------------------------------
## Delimiter: ","
## chr (1): model
## dbl (2): ...1, r2
```

```
## 
## i Use `spec()` to retrieve the full column specification for this data.
## i Specify the column types or set `show_col_types = FALSE` to quiet this message.
```

```
ort_c <- read_csv("ort_outrf_contact_ori.csv")
```

```
## New names:
## * `` -> ...1
```

```
## Rows: 1056 Columns: 3
```

```
## -- Column specification --------------------------------------------------------
## Delimiter: ","
## chr (1): model
## dbl (2): ...1, r2
```

```
## 
## i Use `spec()` to retrieve the full column specification for this data.
## i Specify the column types or set `show_col_types = FALSE` to quiet this message.
```

```
proteu_p <- read_csv("proteu_outrf_policy_ori.csv")
```

```
## New names:
## * `` -> ...1
```

```
## Rows: 1056 Columns: 3
```

```
## -- Column specification --------------------------------------------------------
## Delimiter: ","
## chr (1): model
## dbl (2): ...1, r2
```

```
## 
## i Use `spec()` to retrieve the full column specification for this data.
## i Specify the column types or set `show_col_types = FALSE` to quiet this message.
```

```
proteu_h <- read_csv("proteu_outrf_hygiene_ori.csv")
```

```
## New names:
## * `` -> ...1
```

```
## Rows: 1056 Columns: 3
```

```
## -- Column specification --------------------------------------------------------
## Delimiter: ","
## chr (1): model
## dbl (2): ...1, r2
```

```
## 
## i Use `spec()` to retrieve the full column specification for this data.
## i Specify the column types or set `show_col_types = FALSE` to quiet this message.
```

```
proteu_c <- read_csv("proteu_outrf_contact_ori.csv")
```

```
## New names:
## * `` -> ...1
```

```
## Rows: 1056 Columns: 3
```

```
## -- Column specification --------------------------------------------------------
## Delimiter: ","
## chr (1): model
## dbl (2): ...1, r2
```

```
## 
## i Use `spec()` to retrieve the full column specification for this data.
## i Specify the column types or set `show_col_types = FALSE` to quiet this message.
```

## Variable importance plots

This section presents variable importance plots for each region of Inglehart-Welzel (2020) cultural map. Firstly we check which model provided optimal results with respect to *R2*, followed by calculating permutation variable importance based on these optimal models.

### African-Islamic countries

```
library(plotly)
```

```
## 
## Attaching package: 'plotly'
```

```
## The following object is masked from 'package:ggplot2':
## 
##     last_plot
```

```
## The following object is masked from 'package:stats':
## 
##     filter
```

```
## The following object is masked from 'package:graphics':
## 
##     layout
```

```
afis_c[which.max(afis_c$r2), ] #.131
```

```
## # A tibble: 1 x 3
##    ...1 model                    r2
##   <dbl> <chr>                 <dbl>
## 1   105 trees1000_mtry9_mns12 0.131
```

```
afis_p[which.max(afis_p$r2), ] #-.0225 - highly suboptimal model
```

```
## # A tibble: 1 x 3
##    ...1 model                     r2
##   <dbl> <chr>                  <dbl>
## 1    34 trees2000_mtry5_mns6 -0.0225
```

```
afis_h[which.max(afis_h$r2), ] #.201
```

```
## # A tibble: 1 x 3
##    ...1 model                   r2
##   <dbl> <chr>                <dbl>
## 1    71 trees1000_mtry8_mns9 0.201
```

```
sf3 <- subset(sf2, cgr == "AFIS")
afis_con <- ranger(phc~.-country-cgr-w8-phg-phs, data = sf3, num.trees = 1000, importance = "permutation", num.threads = 10, mtry = 9, min.node.size = 12, seed = 362, case.weights = sf3$w8, holdout = T, splitrule = "variance")
afis_hyg <- ranger(phg~.-country-cgr-w8-phc-phs, data = sf3, num.trees = 1000, importance = "permutation", num.threads = 10, mtry = 8, min.node.size = 9, seed = 362, case.weights = sf3$w8, holdout = T, splitrule = "variance")
afis_pol <- ranger(phs~.-country-cgr-w8-phc-phg, data = sf3, num.trees = 2000, importance = "permutation", num.threads = 10, mtry = 5, min.node.size = 6, seed = 362, case.weights = sf3$w8, holdout = T, splitrule = "variance")
```

### Catholic European countries

```
cateu_c[which.max(cateu_c$r2), ] #.195
```

```
## # A tibble: 1 x 3
##    ...1 model                     r2
##   <dbl> <chr>                  <dbl>
## 1   813 trees1000_mtry11_mns78 0.195
```

```
cateu_p[which.max(cateu_p$r2), ] #.167
```

```
## # A tibble: 1 x 3
##    ...1 model                   r2
##   <dbl> <chr>                <dbl>
## 1    34 trees2000_mtry5_mns6 0.167
```

```
cateu_h[which.max(cateu_h$r2), ] #.163
```

```
## # A tibble: 1 x 3
##    ...1 model                    r2
##   <dbl> <chr>                 <dbl>
## 1   295 trees1000_mtry8_mns30 0.163
```

```
sf3 <- subset(sf2, cgr == "CATEU")
cateu_con <- ranger(phc~.-country-cgr-w8-phg-phs, data = sf3, num.trees = 1000, importance = "permutation", num.threads = 10, mtry = 11, min.node.size = 78, seed = 362, case.weights = sf3$w8, holdout = T, splitrule = "variance")
cateu_hyg <- ranger(phg~.-country-cgr-w8-phc-phs, data = sf3, num.trees = 1000, importance = "permutation", num.threads = 10, mtry = 8, min.node.size = 30, seed = 362, case.weights = sf3$w8, holdout = T, splitrule = "variance")
cateu_pol <- ranger(phs~.-country-cgr-w8-phc-phg, data = sf3, num.trees = 2000, importance = "permutation", num.threads = 10, mtry = 5, min.node.size = 6, seed = 362, case.weights = sf3$w8, holdout = T, splitrule = "variance")
```

### Protestant European countries

```
proteu_c[which.max(proteu_c$r2), ] #.105
```

```
## # A tibble: 1 x 3
##    ...1 model                    r2
##   <dbl> <chr>                 <dbl>
## 1   129 trees1000_mtry5_mns15 0.105
```

```
proteu_p[which.max(proteu_p$r2), ] #.112
```

```
## # A tibble: 1 x 3
##    ...1 model                   r2
##   <dbl> <chr>                <dbl>
## 1     6 trees2000_mtry7_mns3 0.112
```

```
proteu_h[which.max(proteu_h$r2), ] #.116
```

```
## # A tibble: 1 x 3
##    ...1 model                   r2
##   <dbl> <chr>                <dbl>
## 1    33 trees1000_mtry5_mns6 0.116
```

```
sf3 <- subset(sf2, cgr == "PROTEU")
proteu_con <- ranger(phc~.-country-cgr-w8-phg-phs, data = sf3, num.trees = 1000, importance = "permutation", num.threads = 10, mtry = 5, min.node.size = 15, seed = 362, case.weights = sf3$w8, holdout = T, splitrule = "variance")
proteu_hyg <- ranger(phg~.-country-cgr-w8-phc-phs, data = sf3, num.trees = 2000, importance = "permutation", num.threads = 10, mtry = 7, min.node.size = 3, seed = 362, case.weights = sf3$w8, holdout = T, splitrule = "variance")
proteu_pol <- ranger(phs~.-country-cgr-w8-phc-phg, data = sf3, num.trees = 1000, importance = "permutation", num.threads = 10, mtry = 5, min.node.size = 6, seed = 362, case.weights = sf3$w8, holdout = T, splitrule = "variance")
```

### English-speaking countries

```
es_c[which.max(es_c$r2), ] #.247
```

```
## # A tibble: 1 x 3
##    ...1 model                    r2
##   <dbl> <chr>                 <dbl>
## 1   581 trees1000_mtry7_mns57 0.247
```

```
es_p[which.max(es_p$r2), ] #.082
```

```
## # A tibble: 1 x 3
##    ...1 model                     r2
##   <dbl> <chr>                  <dbl>
## 1   610 trees2000_mtry5_mns60 0.0824
```

```
es_h[which.max(es_h$r2), ] #.187
```

```
## # A tibble: 1 x 3
##    ...1 model                    r2
##   <dbl> <chr>                 <dbl>
## 1   195 trees1000_mtry6_mns21 0.187
```

```
sf3 <- subset(sf2, cgr == "ES")
es_con <- ranger(phc~.-country-cgr-w8-phg-phs, data = sf3, num.trees = 1000, importance = "permutation", num.threads = 10, mtry = 7, min.node.size = 57, seed = 362, case.weights = sf3$w8, holdout = T, splitrule = "variance")
es_hyg <- ranger(phg~.-country-cgr-w8-phc-phs, data = sf3, num.trees = 1000, importance = "permutation", num.threads = 10, mtry = 6, min.node.size = 21, seed = 362, case.weights = sf3$w8, holdout = T, splitrule = "variance")
es_pol <- ranger(phs~.-country-cgr-w8-phc-phg, data = sf3, num.trees = 2000, importance = "permutation", num.threads = 10, mtry = 5, min.node.size = 60, seed = 362, case.weights = sf3$w8, holdout = T, splitrule = "variance")
```

### West and South Asian countries

```
sa_c[which.max(sa_c$r2), ] #.105
```

```
## # A tibble: 1 x 3
##    ...1 model                   r2
##   <dbl> <chr>                <dbl>
## 1    36 trees2000_mtry6_mns6 0.105
```

```
sa_p[which.max(sa_p$r2), ] #.305
```

```
## # A tibble: 1 x 3
##    ...1 model                   r2
##   <dbl> <chr>                <dbl>
## 1     1 trees1000_mtry5_mns3 0.305
```

```
sa_h[which.max(sa_h$r2), ] #.217
```

```
## # A tibble: 1 x 3
##    ...1 model                   r2
##   <dbl> <chr>                <dbl>
## 1     1 trees1000_mtry5_mns3 0.217
```

```
sf3 <- subset(sf2, cgr == "SA")
sa_con <- ranger(phc~.-country-cgr-w8-phg-phs, data = sf3, num.trees = 2000, importance = "permutation", num.threads = 10, mtry = 6, min.node.size = 6, seed = 362, case.weights = sf3$w8, holdout = T, splitrule = "variance")
sa_hyg <- ranger(phg~.-country-cgr-w8-phc-phs, data = sf3, num.trees = 1000, importance = "permutation", num.threads = 10, mtry = 5, min.node.size = 3, seed = 362, case.weights = sf3$w8, holdout = T, splitrule = "variance")
sa_pol <- ranger(phs~.-country-cgr-w8-phc-phg, data = sf3, num.trees = 1000, importance = "permutation", num.threads = 10, mtry = 5, min.node.size = 3, seed = 362, case.weights = sf3$w8, holdout = T, splitrule = "variance")
```

### Orthodox European countries

```
ort_c[which.max(ort_c$r2), ] #.062
```

```
## # A tibble: 1 x 3
##    ...1 model                    r2
##   <dbl> <chr>                 <dbl>
## 1    69 trees1000_mtry7_mns9 0.0619
```

```
ort_p[which.max(ort_p$r2), ] #.114
```

```
## # A tibble: 1 x 3
##    ...1 model                     r2
##   <dbl> <chr>                  <dbl>
## 1   543 trees1000_mtry20_mns51 0.114
```

```
ort_h[which.max(ort_h$r2), ] #.126
```

```
## # A tibble: 1 x 3
##    ...1 model                   r2
##   <dbl> <chr>                <dbl>
## 1     1 trees1000_mtry5_mns3 0.126
```

```
sf3 <- subset(sf2, cgr == "ORT")
ort_con <- ranger(phc~.-country-cgr-w8-phg-phs, data = sf3, num.trees = 1000, importance = "permutation", num.threads = 10, mtry = 7, min.node.size = 9, seed = 362, case.weights = sf3$w8, holdout = T, splitrule = "variance")
ort_hyg <- ranger(phg~.-country-cgr-w8-phc-phs, data = sf3, num.trees = 1000, importance = "permutation", num.threads = 10, mtry = 5, min.node.size = 3, seed = 362, case.weights = sf3$w8, holdout = T, splitrule = "variance")
ort_pol <- ranger(phs~.-country-cgr-w8-phc-phg, data = sf3, num.trees = 1000, importance = "permutation", num.threads = 10, mtry = 20, min.node.size = 51, seed = 362, case.weights = sf3$w8, holdout = T, splitrule = "variance")
```

### Latin American countries

```
latam_c[which.max(latam_c$r2), ] #.093
```

```
## # A tibble: 1 x 3
##    ...1 model                     r2
##   <dbl> <chr>                  <dbl>
## 1   962 trees2000_mtry5_mns93 0.0934
```

```
latam_p[which.max(latam_p$r2), ] #.084
```

```
## # A tibble: 1 x 3
##    ...1 model                     r2
##   <dbl> <chr>                  <dbl>
## 1   711 trees1000_mtry8_mns69 0.0846
```

```
latam_h[which.max(latam_h$r2), ] #.138
```

```
## # A tibble: 1 x 3
##    ...1 model                    r2
##   <dbl> <chr>                 <dbl>
## 1   257 trees1000_mtry5_mns27 0.138
```

```
sf3 <- subset(sf2, cgr == "LATAM")
latam_con <- ranger(phc~.-country-cgr-w8-phg-phs, data = sf3, num.trees = 2000, importance = "permutation", num.threads = 10, mtry = 5, min.node.size = 93, seed = 362, case.weights = sf3$w8, holdout = T, splitrule = "variance")
latam_hyg <- ranger(phg~.-country-cgr-w8-phc-phs, data = sf3, num.trees = 1000, importance = "permutation", num.threads = 10, mtry = 5, min.node.size = 27, seed = 362, case.weights = sf3$w8, holdout = T, splitrule = "variance")
latam_pol <- ranger(phs~.-country-cgr-w8-phc-phg, data = sf3, num.trees = 1000, importance = "permutation", num.threads = 10, mtry = 8, min.node.size = 69, seed = 362, case.weights = sf3$w8, holdout = T, splitrule = "variance")
```

### Confucian countries

```
conf_c[which.max(conf_c$r2), ] #.220
```

```
## # A tibble: 1 x 3
##    ...1 model                    r2
##   <dbl> <chr>                 <dbl>
## 1    13 trees1000_mtry11_mns3 0.220
```

```
conf_p[which.max(conf_p$r2), ] #.176
```

```
## # A tibble: 1 x 3
##    ...1 model                    r2
##   <dbl> <chr>                 <dbl>
## 1    45 trees1000_mtry11_mns6 0.176
```

```
conf_h[which.max(conf_h$r2), ] #.281
```

```
## # A tibble: 1 x 3
##    ...1 model                    r2
##   <dbl> <chr>                 <dbl>
## 1    12 trees2000_mtry10_mns3 0.281
```

```
sf3 <- subset(sf2, cgr == "SA")
conf_con <- ranger(phc~.-country-cgr-w8-phg-phs, data = sf3, num.trees = 1000, importance = "permutation", num.threads = 10, mtry = 11, min.node.size = 3, seed = 362, case.weights = sf3$w8, holdout = T, splitrule = "variance")
conf_hyg <- ranger(phg~.-country-cgr-w8-phc-phs, data = sf3, num.trees = 2000, importance = "permutation", num.threads = 10, mtry = 10, min.node.size = 3, seed = 362, case.weights = sf3$w8, holdout = T, splitrule = "variance")
conf_pol <- ranger(phs~.-country-cgr-w8-phc-phg, data = sf3, num.trees = 1000, importance = "permutation", num.threads = 10, mtry = 11, min.node.size = 6, seed = 362, case.weights = sf3$w8, holdout = T, splitrule = "variance")
```

# Variable importance plots

The following plot represent permutation variable importance of studied predictors. More about permutation variable importance can be found here. Although the importance estimates across models are not directly comparable as they are bound to error estimate, the substantial differences in patterns could suggest that importance of our predictors varies across cultural regions.

Firstly, one can notice the difference in relative heights of columns, which is lowest among Latin American countries. This is in line with somewhat lower *R2* values established in these countries and indicates that our predictors were less effective in this region.

Secondly, it is easy to notice a relatively similar patterns across the cultural map: variables ranging from CRT to moral circle on the x-axis of the plot exhibit a very limited contribution to the accuracy of prediction. On the other hand, contribution of remaining predictors seems to substantially vary, both with respect to the specific criterion and cultural region. Nevertheless, variables reflecting morality, social belonging, open-mindedness and collective narcissism seem to be among the most substantial predictors of attitudinal and behavioral responses to COVID-19 in our data set.

The plot is interactive - depending on the interest or purpose, readers can turn on and off specific columns, zoom on variables of interest etc.

```
vimpp_c <- data.frame(afis_contact = afis_con$variable.importance, afis_hygiene = afis_hyg$variable.importance, afis_policy = afis_pol$variable.importance,
                         conf_contact = conf_con$variable.importance, conf_hygiene = conf_hyg$variable.importance, conf_policy = conf_pol$variable.importance,
                         cateu_contact = cateu_con$variable.importance, cateu_hygiene = cateu_hyg$variable.importance, cateu_policy = cateu_pol$variable.importance,
                         proteu_contact = proteu_con$variable.importance, proteu_hygiene = proteu_hyg$variable.importance, proteu_policy = proteu_pol$variable.importance,
                         sa_contact = sa_con$variable.importance, sa_hygiene = sa_hyg$variable.importance, sa_policy = sa_pol$variable.importance,
                         es_contact = es_con$variable.importance, es_hygiene = es_hyg$variable.importance, es_policy = es_pol$variable.importance,
                         latam_contact = latam_con$variable.importance, latam_hygiene = latam_hyg$variable.importance, latam_policy = latam_pol$variable.importance,
                         ort_contact = ort_con$variable.importance, ort_hygiene = ort_hyg$variable.importance, ort_policy = ort_pol$variable.importance)

vimpp_c$var <- c("collective narcissism", "conspiracy theories", "open-mindedness", "morality - cooperation", "belonging", "selfcontrol", "narcissism - admiration", "narcissism - rivalry", "moral identity - internalization", "moral identity - symbolization", "optimism", "risk perception", "national identity", "self-esteem", "SES", "moral circle", "health", "political ideology", "CRT", "tested positive", "knows positive", "urban residence", "marital status", "employment status", "age", "children", "sex")
vp2 <- pivot_longer(vimpp_c, 1:24)
vp2 <- separate(vp2, "name", c("region", "criterion"), sep = "_")
vp2$region <- factor(vp2$region, levels = c("afis", "ort", "cateu", "proteu", "latam", "es", "sa", "conf"), labels = c("African-Islamic", "Orthodox Europe", "Catholic Europe", "Protestant Europe", "Latin America", "English-Speaking", "West and South Asia", "Confucian"))
```

```
plt <- ggplot(vp2, aes(x = var, y = value, fill = criterion)) + 
        geom_bar(stat = "identity", color = "black", position = "dodge") +
        theme_bw() +
        theme(axis.text.x = element_text(angle = 90, vjust = .5, hjust = 1, size = 12), text = element_text(family = "serif", size = 12)) +
        xlab("") +
        ylab("permutation importance") +
        scale_fill_manual("criterion", values = c("white", "gray50", "black")) + 
        facet_wrap(.~region, nrow = 4)
ggplotly(plt)
```

# Partial dependence plots

Finally, we visualized our findings in the form of partial dependence plots, which can help with interpretation of the relationships between predictors and our three criteria. Relationships with **avoiding contact** are shown in **red**, relationships with **maintaining hygiene** are colored in **blue**, while relationships with **policy support** are colored in **green**.

## African-Islamic countries

Partial dependence plot

## Orthodox Europe

Partial dependence plot

## Catholic Europe

Partial dependence plot

## Protestant Europe

Partial dependence plot

## English-Speaking

Partial dependence plot

## Latin America

Partial dependence plot

## Confucian

Partial dependence plot

## West and South Asia

Partial dependence plot

# SessionInfo

```
sessionInfo()
```

```
## R version 4.1.2 (2021-11-01)
## Platform: x86_64-w64-mingw32/x64 (64-bit)
## Running under: Windows 10 x64 (build 22000)
## 
## Matrix products: default
## 
## locale:
## [1] LC_COLLATE=Croatian_Croatia.1250  LC_CTYPE=Croatian_Croatia.1250   
## [3] LC_MONETARY=Croatian_Croatia.1250 LC_NUMERIC=C                     
## [5] LC_TIME=Croatian_Croatia.1250    
## system code page: 1252
## 
## attached base packages:
## [1] stats     graphics  grDevices utils     datasets  methods   base     
## 
## other attached packages:
##  [1] plotly_4.9.4.1 ranger_0.13.1  ggplot2_3.3.5  tidyr_1.1.3    seminr_2.1.0  
##  [6] readr_2.0.2    semPlot_1.1.2  semTools_0.5-5 lavaan_0.6-9   psych_2.1.6   
## [11] dplyr_1.0.7   
## 
## loaded via a namespace (and not attached):
##   [1] TH.data_1.1-0       minqa_1.2.4         colorspace_2.0-2   
##   [4] ellipsis_0.3.2      estimability_1.3    htmlTable_2.2.1    
##   [7] corpcor_1.6.9       base64enc_0.1-3     rstudioapi_0.13    
##  [10] bit64_4.0.5         fansi_0.5.0         mvtnorm_1.1-2      
##  [13] codetools_0.2-18    splines_4.1.2       mnormt_2.0.2       
##  [16] knitr_1.33          glasso_1.11         Formula_1.2-4      
##  [19] jsonlite_1.7.2      nloptr_1.2.2.2      cluster_2.1.2      
##  [22] png_0.1-7           regsem_1.8.0        httr_1.4.2         
##  [25] compiler_4.1.2      emmeans_1.6.1       backports_1.2.1    
##  [28] lazyeval_0.2.2      assertthat_0.2.1    Matrix_1.3-4       
##  [31] fastmap_1.1.0       cli_3.0.1           htmltools_0.5.2    
##  [34] tools_4.1.2         igraph_1.2.6        OpenMx_2.19.6      
##  [37] coda_0.19-4         gtable_0.3.0        glue_1.4.2         
##  [40] reshape2_1.4.4      Rcpp_1.0.7          carData_3.0-4      
##  [43] jquerylib_0.1.4     vctrs_0.3.8         nlme_3.1-153       
##  [46] lisrelToR_0.1.4     crosstalk_1.1.1     xfun_0.24          
##  [49] stringr_1.4.0       openxlsx_4.2.4      lme4_1.1-27.1      
##  [52] lifecycle_1.0.0     gtools_3.9.2        XML_3.99-0.6       
##  [55] MASS_7.3-54         zoo_1.8-9           scales_1.1.1       
##  [58] vroom_1.5.5         hms_1.1.0           kutils_1.70        
##  [61] parallel_4.1.2      sandwich_3.0-1      RColorBrewer_1.1-2 
##  [64] yaml_2.2.1          pbapply_1.4-3       gridExtra_2.3      
##  [67] sass_0.4.0          rpart_4.1-15        latticeExtra_0.6-29
##  [70] stringi_1.6.2       highr_0.9           sem_3.1-11         
##  [73] checkmate_2.0.0     boot_1.3-28         zip_2.2.0          
##  [76] truncnorm_1.0-8     rlang_0.4.11        pkgconfig_2.0.3    
##  [79] Rsolnp_1.16         arm_1.11-2          evaluate_0.14      
##  [82] lattice_0.20-45     purrr_0.3.4         labeling_0.4.2     
##  [85] htmlwidgets_1.5.3   bit_4.0.4           tidyselect_1.1.1   
##  [88] plyr_1.8.6          magrittr_2.0.1      R6_2.5.1           
##  [91] generics_0.1.0      Hmisc_4.5-0         multcomp_1.4-17    
##  [94] DBI_1.1.1           withr_2.4.2         pillar_1.6.2       
##  [97] foreign_0.8-81      rockchalk_1.8.144   survival_3.2-13    
## [100] abind_1.4-5         nnet_7.3-16         tibble_3.1.4       
## [103] crayon_1.4.1        fdrtool_1.2.16      utf8_1.2.2         
## [106] tmvnsim_1.0-2       tzdb_0.1.2          rmarkdown_2.9      
## [109] jpeg_0.1-8.1        grid_4.1.2          qgraph_1.6.9       
## [112] data.table_1.14.0   pbivnorm_0.6.0      matrixcalc_1.0-4   
## [115] digest_0.6.27       xtable_1.8-4        mi_1.0             
## [118] RcppParallel_5.1.4  stats4_4.1.2        munsell_0.5.0      
## [121] viridisLite_0.4.0   bslib_0.3.1
```

# Thank you for your attention!
