# Supplementary material for: Predicting attitudinal and behavioral responses to COVID-19 pandemic using machine learning
Source: PNAS Nexus. 2022 Jul 5;1(3):pgac093. doi: 10.1093/pnasnexus/pgac093 (PMC9381137; doi:10.1093/pnasnexus/pgac093)
Supplement: pgac093_Supplemental_Files [file pgac093_supplemental_files.zip › Supplementary_materials_A._Theoretical_basis.docx]

# Appendix A. Theoretical and empirical basis of the choice of predictors

This supplementary file contains a description of theoretical contributions of specific factors to the prediction of COVID-19 responses. Additionally, it also contains some empirical outcomes of recent studies that used these factors in the prediction of COVID-19 responses. Due to the relatively large number of included constructs, we aimed at providing brief information on each of them rather than creating a detailed overview. First, the role of morality is described, followed by constructs from social, personality and cognitive psychology, socio-demographic constructs and a brief discussion on potential country-level differences.

M**orality** is an important factor in understanding how people follow regulations in stressful social scenarios, activating selfish versus collaborative behaviours (Gino et al., 2016; Van Bavel et al., 2020). Furthermore, the prosocial promotion of help and others' care is partially determined by moral attitudes (Decety et al., 2016; Zaki et al., 2012). Here we considered three different dimensions of morality: identity (self-identification process based on moral prosocial attributes) (Aquino & Reed, 2002), circle/expansiveness (i.e., the range of entities what a person considers worthy of moral concern and treatment) (Waytz et al., 2019) and cooperation (indexing different cooperative behaviours such as helping groups) (Curry et al., 2019). There is some initial evidence that segments in the general public (specifically in Germany) that can be distinguished based on their agreement with different claims about the COVID-19 pandemic (both true and false assumptions about the course of the infection or the handling of the infection or the pandemic and risk claims) also differ with regards to the importance of moral identity and the breath of their moral circle (Rothmund et al., 2020). However, their influence on behavioural responses to pandemic scenarios is largely unknown.

Self-isolation and physical distancing have been widely recommended across countries as some of the most effective measures to prevent transmission of the virus. However, this has posed a major challenge to people's social life, belongingness to social groups and connection with others (Baumeister & Leary, 1995). **Belonging to social groups** and development of connections with others enhances people's coping skills when they encounter adversities, making them more resilient and competent to regulate their emotions (Jetten et al., 2012; Williams et al., 2018). Community-belonging, in particular, has been found to positively contribute to beneficial health-behaviour change making it a relevant factor to consider in public health interventions and prevention strategies (Hystad & Carpiano, 2012). On the other hand, loneliness and isolation from others cause stress with negative effects on people's mental and physical health (Hawkley & Cacioppo, 2010). In the context of COVID-19 pandemic, there is some evidence that social exclusion is predictive of endorsing COVID-19 conspiracy theories (Stoica & Umbres, 2021) which in turn may have adverse consequences such as risk denial and irresponsible behaviour (van Mulukom et al., 2020). Overall, the outlined findings suggest social belonging may be relevant to the pandemic response.

**National identification** refers to the importance of being a member of a nation to a person's self-identity (Postmes et al., 2012). Classic social identity theories suggest that identification with a particular group predicts conformity to group norms and collective actions that would advance the group's welfare (Tajfel, 1978; van Zomeren et al., 2008). When facing a global crisis, such as a pandemic, government officials often frame the crisis as a shared national problem (e.g., "Together we fight the virus") (Gkinopoulos & Hegarty, 2018). The activated national identities thus play a crucial role in motivating collective efforts to engage in public health support (Van Bavel et al., 2020).

COVID-19 is a public health and policy issue, often a divisive topic, that might be subject to **politicization and polarization**. Similar to other scientific issues of public interest, most notably global warming (Dunlap & Jacques, 2013; Luo & Zhao, 2019; McCright & Dunlap, 2011; Whitmarsh, 2011), there is evidence that party affiliation and self-placed ideological orientation plays a relevant role in explaining COVID-19 related attitudes and behaviour, at least in some countries, such as the US and Canada. Namely, findings indicate that liberals and Democrats are more likely to be concerned of the virus health impact, they see lockdown as an appropriate measure, also have more faith in scientists, are better at discerning between real and fake COVID-19 related news, and report increased preventive behaviour compared to Republicans and conservatives (Calvillo et al., 2020; Pennycook et al., 2020a; 2020b; Pickup et al., 2020; Rothgerber et al., 2020; van Holm et al., 2020). Research utilizing objective behavioural measures, i.e., GPS-based data, data on internet searches, and debit card transactions, provide additional evidence of the partisan and ideological divide concerning public response to the pandemic (Allcott et al., 2020; Barrios & Hochberg, 2020; Gollwitzer et al., 2020; Painter & Qiu, 2020). Other research also suggests that ideology is a relevant factor in predicting what kind of measures will be supported around the world. Left oriented policies prioritize social cohesion, protection of the vulnerable groups (elderly, lower strata, ethnic minorities, migrants etc.), while right-oriented policies tend to prioritize the economy and scapegoat minorities as transmitters of the disease ([Stavrakakis & Katsampekis, 2020)](http://populismus.gr/wp-content/uploads/2020/06/interventions-7-populism-pandemic-UPLOAD.pdf). At the same time, research regarding the role of partisan and ideological orientation in understanding attitudes and behaviours relating to the COVID-19 pandemic in other countries is needed.

Along these lines, both **collective** and **individual narcissism** are of interest. **Individual grandiose narcissism** is characterized by affective-motivational, cognitive and behavioural processes that involve the needs for admiration and rivalry to maintain a positive self-concept (Back et al., 2013). **Collective narcissism** is an extension of individual grandiose narcissism onto the intergroup level involving a grandiose view of the ingroup that requires constant external validation (Golec de Zavala et al., 2009). COVID-19 can be considered as a threat to self and group, and findings have shown individual and collective narcissism to be associated with attitudes and behaviours during the COVID-19 pandemic (Nowak et al., 2020). Individual narcissism correlates negatively with agreeableness (Bradlee & Emmons, 1992; Miller & Maples, 2011) and positively with impulsivity (Jones & Paulhus, 2011; Vazire & Funder, 2006) and risk-taking tendencies (Crysel et al., 2013), and recent findings in the context of the COVID-19 pandemic indicate it is negatively associated with preventive behaviour and positively with hoarding via coronavirus-related health beliefs (e.g. perceived susceptibility, Nowak et al., 2020) and lower compliance with governmental restrictions (Zajenkowski et al., 2020). As to collective narcissism, it was found to correlate positively with perceived outgroup threat to the ingroup's image, prejudice, conspiracy theories relating to the outgroup (Cichocka et al., 2016; Golec de Zavala et al., 2013; Marchlewska et al., 2013). Furthermore, in the context of the current crises, it is shown to be related to hoarding behaviour (Nowak et al., 2020), but the evidence is mixed regarding the relationship with preventive behaviours. Namely, in one study, although it was found to be a positive predictor of handwashing, it did not predict physical distancing (Sternisko et al., 2020). On the other hand, in another study, collective narcissism was unrelated to preventive behaviours (disinfecting, handwashing, limiting leaving home; Nowak et al., 2020).

**Self-control** has been found to affect adherence to physical distancing measures during the current pandemic (Wolff et al., 2020). Self-control constitutes a trait that helps people overcome demanding responses to multifaceted threats and complicated situations in order to reach the desired goal (Shenhav et al., 2013). Thus, in order to adhere to guidelines regarding physical distancing during the pandemic, people need self-control. Responding to demanding or threatening situations with self-control can be an effortful process, which may signal the potential cost of applying self-control (Shenhav et al., 2017). As Kurzban et al. (2013) suggested, self-control is applied only if its benefit outweighs its cost. Thus, it becomes evident how attitudes and behaviours are directly associated with self-control in responses to COVID-19 related guidelines. People can perceive specific benefits when adhering to physical distancing measures, thus exerting high self-control (Wolff & Martarelli, 2020). On the other hand, people with low self-control might be less keen to adhere to physical distancing measures, because adherence per se is experienced as an action that demands high levels of self-control (Wolff et al., 2020).

High **self-esteem** has been associated with a number of positive outcomes and characteristics, such as increased psychological well-being (Paradise & Kernis, 2002) and higher emotional intelligence (Schutte et al., 2002). It has also been shown that individuals high in self-esteem underestimate the negative personal consequences of risky health behaviour (Gerrard et al., 2001), which, in the context of the pandemic, could lead to such individuals underestimating the risk of non-compliance with prevention guidelines and policies. On the other hand, low self-esteem has been shown to be related to externalizing problems, including antisocial behaviour (Donnellan et al. 2005), which too could lead such individuals to refrain from supporting public health behaviours. In either case, more research and evidence are needed to understand the role of self-esteem.

Several works explored the **role of optimism** regarding epidemic related attitudes and behaviour. As a trait, optimism has been linked to a greater capacity to cope with stressful situations (Scheier et al., 1986), and it was associated with vigilance during the SARS epidemic, whereas pessimism was associated with anxiety (Xie et al., 2011). In addition, beliefs in conspiracy theories have been linked to pessimism (Furnham, 2013). During the COVID-19 pandemic, evidence has been found that optimism is associated with lower levels of fear and higher levels of preventive behaviour, and pessimism with higher levels of fear (Jovančević & Milićević, 2020). A study on a Turkish adult sample found evidence for mediating effects of optimism and pessimism on the relationship of COVID-19 related stress as well as psychological inflexibility with psychological problems, indicating that higher optimism and lower pessimism can reduce their negative effects on psychological problems (Arslan et al., 2020). On the other hand, previous findings suggest that unrealistically optimistic individuals, inclined to underestimate own susceptibility to infectious diseases and other illnesses, may exhibit less interest in risk reduction actions (Weinstein, 1987; 1989). This **optimism bias** has been observed in the context of the COVID-19 pandemic in Italy and Romania (Druica et al., 2020), the United States (Wise et al., 2020), as well as in Poland (Dolinski et al., 2020). A UK-based study found that people display unrealistic optimism regarding COVID-19 related risks in the short term, but unrealistic pessimism when projecting themselves one-year ahead (Asimakopoulou et al., 2020). Hence, in present work, we opted to measure trait optimism as well as unrealistic optimism regarding the COVID-19 pandemic, by comparing the perceived likelihood that one gets infected with the perceived likelihood that others get infected.

The COVID-19 issue is a complex scientific, public health and policy issue. How people assess COVID-19 risks and whether they support COVID-19 policies and guidelines involves both autonomously cued, typically intuitive and fast ("type 1") processes as well as effortful, typically resource-demanding cognitive ("type 2") processes of risk assessment and decision making (Stanovich et al., 2016). **Risk perception** has been widely investigated in the context of threatening and catastrophic situations and events (such as natural hazards and epidemics) and motivation for mitigation behaviour (e.g., Bubeck et al., 2012; Liao et al., 2019; Wachinger et al., 2013; Xu et al., 2018). Overall, the findings provide ground to assume that subjective assessment of the probability and consequences of adverse outcomes might be linked to psychological and behavioural responses in the context of a new threat surrounded with much uncertainty and unpredictability that is COVID-19. Indeed, recent studies have suggested its positive effect on adherence to preventive behaviour (Dryhurst et al., 2020; Yildirim et al., 2021). However, research has informed us that risk perception is a complex process influenced by a multitude of factors relating to both perceiving hazards (e.g., novelty, controllability, dread) and perceivers themselves (e.g., expertise, scientific reasoning ability, reasoning styles, the tendency towards optimism bias), as well as their interplay (Wachinger et al., 2013; Gaissmaier, 2019). Along these lines, personal experience of various hazards and proximity to such experience may play a relevant role. On the one side, it could reinforce protective and preventive behaviour, as the experience itself becomes a tangible instead of an abstract risk (Harvatt et al., 2011; Norris et al., 1999; Weinstein, 1989). Conversely, seldom experienced hazards and direct experience of low severity can lead to a false sense of security and constrain motivation for mitigation behaviour (Harvatt et al., 2011; Scolobig et al., 2012; Weinstein, 1989). In the context of the current pandemic, one such experiential factor that could drive risk perceptions and affect COVID-19 related attitudes and behaviours is direct **experience of being tested positive** or **experience in knowing someone who tested positive for COVID-19**. There is already some evidence indicating that having direct experience with the coronavirus is related to higher COVID-19 risk perception (Dryhurst et al., 2020) and that experience (direct and indirect) is a distal predictor (through affective attitude and risk perception) of protective behaviours (Savadori & Lauriola, 2020). These findings generally speak to the relevance of affective/experiential way of risk perception, as one of the two fundamental pathways within the modern frameworks of risk perception and management (Loewenstein et al., 2001; Slovic et al., 2004). Namely, the vividness of the experienced negative consequences might elicit strong affective reactions and engage the fast, effortless, efficient affective/experiential processing that can influence subsequent actions and decisions (Bronfman et al., 2020; Loewenstein et al., 2001; Weber, 2006).

At the same time, cognitive-analytical processes are also relevant in judgement and decision‐making circumstances (Stanovich et al., 2016). **Two cognitive factors**, indicative of sophistication in cognitive-analytical processing that may prove relevant in the context of COVID-19 are a person's capability to process available information with reasoned thinking (i.e., **cognitive reflection**) and willingness to take in such information (i.e., **open-mindedness**). Namely, previous research provides some evidence that **cognitive reflection**, ability and tendency of overriding impulsive responses and engaging in reflective thinking (Frederick, 2005) affects individual's risk-taking attitudes and behaviours in various domains (Frederick et al., 2005; Thoma et al., 2015). Furthermore, recent studies indicate that lower levels of cognitive reflection are related to beliefs in various pseudo-scientific practices and misinformation and conspiracy theories regarding COVID-19 (Alper et al., 2020; Čavojová et al., 2020; Erceg et al., 2020; Stanley et al., 2020; Teovanović et al., 2021). These findings are consistent with a continuously growing body of evidence showing that more analytic and cognitively sophisticated individuals are likely to be less prone to epistemically suspect beliefs (Pennycook et al., 2020c), and more likely to be inclined to scientifically founded beliefs (McPhetres & Pennycook, 2019). On the other hand, evidence on the relationship with adherence to preventive behaviours is somewhat mixed. Some of the studies found positive associations with cognitive reflection (Stanley et al., 2020; Teovanović et al., 2021), some failed to find direct associations with cognitive reflection (Alper et al., 2020; Čavojová et al., 2020; Erceg et al., 2020), and a couple found a significant indirect effect of cognitive reflection on adherence to preventive behaviours via COVID-19 conspiracy beliefs (Erceg et al., 2020; Stanley et al., 2020).

**Open-mindedness,** a disposition or cognitive style of respecting others' intellectual capabilities while recognizing the limitations of one's own, and openness to acquiring new information and knowledge from others regardless of the social status; Alfano et al., 2017) is another cognitive factor worth investigating in the current context. For example, prior work notes that when exposed to a health threat message, open-minded people tend to accept the message and become aware of the negative consequences of non-compliance (Pietersma & Dijkstra, 2012). Moreover, in the context of the current crisis, a study utilizing a variant of the open-mindedness scale (Erceg et al., 2020) found a positive association between open-mindedness and more responsible behaviour. Overall, there are theoretical and empirical reasons for evaluating the role of risk perception, personal experience and indicators of analytical thinking in the effective public response to a complex global crisis.

**Conspiracy theories** associated with COVID-19 pandemic are widespread and relate to a wide range of issues, ranging from nature and COVID-19 origin, different treatments, preventive measures, consequences etc., indexed even on a Wikipedia page^[[1]](#footnote-1)^. The findings indicating that inclination towards such conspiracies might have detrimental consequences are accumulating as reviewed in a preprint article (van Mulukom et al., 2020). This is expected since previous studies suggest that conspiracy theories tend to spread during social and public-health crises (Jolley & Douglas, 2014), including past pandemic (i.e., Zika virus; Klofstad et al., 2019), are modulated by social and group behaviours (Sternisko et al., 2020), and are associated with harmful health-related behaviours (Bogart et al., 2010; Brainard et al., 2020; Grebe & Nattrass, 2012; Klofstad et al., 2019).

**Socio-demographic factors**, such as sex, age, marital status, socioeconomic status, or urban/rural residency, have considerable predictive value on a large number of attitudes and behaviours, ranging from voting to health (e.g., Cassese & Barnes, 2019; Contoyannis & Jones, 2004). As such, these factors may play a substantial role in differences in attitudes and behaviours relating to the COVID-19 pandemic. For example, older age (especially being over 70) has proven to be a relevant risk factor for experiencing severe COVID-19 disease outcomes (Jordan et al., 2020), and it seems plausible to assume that behaviour and COVID-19 policy support may be related to age as well. One might think that this would be particularly pronounced for younger and older age groups, as both groups face rather distinct risk profiles. Furthermore, the urban/rural divide, as well as socioeconomic status, more generally could also be related to COVID-19 attitudes and behaviours because population density and access to healthcare, which are both directly related to residence and socioeconomic status in many parts of the world, might impact the spread of the virus and overall health outcomes in any given region.

**References**

Alfano, M., Iurino, K., Stey, P., Robinson, B., Christen, M., Yu, F., & Lapsley, D. (2017). Development and validation of a multi-dimensional measure of intellectual humility. *PLoS ONE*, *12*(8), e0182950. <https://doi.org/10.1371/journal.pone.0182950>

Allcott, H., Boxell, L., Conway, J., Gentzkow, M., Thaler, M., & Yang, D. (2020). Polarization and public health: Partisan differences in social distancing during the coronavirus pandemic. *Journal of Public Economics*, *191*, 104254. <https://doi.org/10.1016/j.jpubeco.2020.104254>

Alper, S., Bayrak, F., & Yilmaz, O. (2020). Psychological correlates of COVID-19 conspiracy beliefs and preventive measures: Evidence from Turkey. *Current Psychology (New Brunswick, N.j.)*, 1–10. <https://doi.org/10.1007/s12144-020-00903-0>

Aquino, K., & Reed, A. (2002). The self-importance of moral identity. *Journal of Personality and Social Psychology*, *83*(6), 1423–1440. [https://doi.org/10.1037//0022-3514.83.6.1423](https://doi.org/10.1037/0022-3514.83.6.1423)

Arslan, G., Yıldırım, M., Tanhan, A., Buluş, M., & Allen, K.-A. (2020). Coronavirus Stress, Optimism-Pessimism, Psychological Inflexibility, and Psychological Health: Psychometric Properties of the Coronavirus Stress Measure. *International Journal of Mental Health and Addiction*, 1–17. <https://doi.org/10.1007/s11469-020-00337-6>

Asimakopoulou, K., Hoorens, V., Speed, E., Coulson, N. S., Antoniszczak, D., Collyer, F., Deschrijver, E., Dubbin, L., Faulks, D., Forsyth, R., Goltsi, V., Harsløf, I., Larsen, K., Manaras, I., Olczak-Kowalczyk, D., Willis, K., Xenou, T., & Scambler, S. (2020). Comparative optimism about infection and recovery from COVID-19; Implications for adherence with lockdown advice. *Health Expectations*, *23*(6), 1502–1511. <https://doi.org/10.1111/hex.13134>

Back, M. D., Küfner, A. C. P., Dufner, M., Gerlach, T. M., Rauthmann, J. F., & Denissen, J. J. A. (2013). Narcissistic admiration and rivalry: Disentangling the bright and dark sides of narcissism. *Journal of Personality and Social Psychology*, *105*(6), 1013–1037. <https://doi.org/10.1037/a0034431>

Barrios, J. M., & Hochberg, Y. (2020). *Risk Perception Through the Lens of Politics in the Time of the COVID-19 Pandemic* (Working Paper No. 27008; Working Paper Series). National Bureau of Economic Research. <https://doi.org/10.3386/w27008>

Baumeister, R. F., & Leary, M. R. (1995). The need to belong: Desire for interpersonal attachments as a fundamental human motivation. *Psychological Bulletin*, *117*(3), 497–529. <https://doi.org/10.1037/0033-2909.117.3.497>

Bogart, L. M., Wagner, G., Galvan, F. H., & Banks, D. (2010). Conspiracy beliefs about HIV are related to antiretroviral treatment nonadherence among african american men with HIV. *Journal of Acquired Immune Deficiency Syndromes (1999)*, *53*(5), 648–655. <https://doi.org/10.1097/QAI.0b013e3181c57dbc>

Bradlee, P. M., & Emmons, R. A. (1992). Locating narcissism within the interpersonal circumplex and the five-factor model. *Personality and Individual Differences*, *13*(7), 821–830. <https://doi.org/10.1016/0191-8869(92)90056-U>

Brainard, J., & Hunter, P. R. (2020). Misinformation making a disease outbreak worse: Outcomes compared for influenza, monkeypox, and norovirus. *SIMULATION*, *96*(4), 365–374. <https://doi.org/10.1177/0037549719885021>

Bronfman, N. C., Cisternas, P. C., Repetto, P. B., Castañeda, J. V., & Guic, E. (2020). Understanding the Relationship Between Direct Experience and Risk Perception of Natural Hazards. *Risk Analysis*, *40*(10), 2057–2070. <https://doi.org/10.1111/risa.13526>

Bubeck, P., Botzen, W. J. W., & Aerts, J. C. J. H. (2012). A review of risk perceptions and other factors that influence flood mitigation behavior. *Risk Analysis: An Official Publication of the Society for Risk Analysis*, *32*(9), 1481–1495. <https://doi.org/10.1111/j.1539-6924.2011.01783.x>

Calvillo, D. P., Ross, B. J., Garcia, R. J. B., Smelter, T. J., & Rutchick, A. M. (2020). Political Ideology Predicts Perceptions of the Threat of COVID-19 (and Susceptibility to Fake News About It). *Social Psychological and Personality Science*, *11*(8), 1119–1128. <https://doi.org/10.1177/1948550620940539>

Cassese, E. C., & Barnes, T. D. (2019). Reconciling Sexism and Women’s Support for Republican Candidates: A Look at Gender, Class, and Whiteness in the 2012 and 2016 Presidential Races. *Political Behavior*, *41*(3), 677–700. <https://doi.org/10.1007/s11109-018-9468-2>

Čavojová, V., Šrol, J., & Ballová Mikušková, E. (2020). How scientific reasoning correlates with health-related beliefs and behaviors during the COVID-19 pandemic? *Journal of Health Psychology*, 1359105320962266. <https://doi.org/10.1177/1359105320962266>

Cichocka, A., Marchlewska, M., Golec de Zavala, A., & Olechowski, M. (2016). “They will not control us”: Ingroup positivity and belief in intergroup conspiracies. *British Journal of Psychology*, *107*(3), 556–576. <https://doi.org/10.1111/bjop.12158>

Contoyannis, P., & Jones, A. M. (2004). Socio-economic status, health and lifestyle. *Journal of Health Economics*, *23*(5), 965–995. <https://doi.org/10.1016/j.jhealeco.2004.02.001>

Crysel, L. C., Crosier, B. S., & Webster, G. D. (2013). The Dark Triad and risk behavior. *Personality and Individual Differences*, *54*(1), 35–40. <https://doi.org/10.1016/j.paid.2012.07.029>

Curry, O. S., Jones Chesters, M., & Van Lissa, C. J. (2019). Mapping morality with a compass: Testing the theory of ‘morality-as-cooperation’ with a new questionnaire. *Journal of Research in Personality*, *78*, 106–124. <https://doi.org/10.1016/j.jrp.2018.10.008>

Decety, J., Bartal, I., Uzefovsky, F., & Knafo-Noam, A. (2016). Empathy as a driver of prosocial behaviour: Highly conserved neurobehavioural mechanisms across species. *Philosophical Transactions of the Royal Society B: Biological Sciences*, *371*, 20150077. <https://doi.org/10.1098/rstb.2015.0077>

Dolinski, D., Dolinska, B., Zmaczynska-Witek, B., Banach, M., & Kulesza, W. (2020). Unrealistic Optimism in the Time of Coronavirus Pandemic: May It Help to Kill, If So—Whom: Disease or the Person? *Journal of Clinical Medicine*, *9*(5), 1464. <https://doi.org/10.3390/jcm9051464>

Donnellan, M. B., Trzesniewski, K. H., Robins, R. W., Moffitt, T. E., & Caspi, A. (2005). Low Self-Esteem Is Related to Aggression, Antisocial Behavior, and Delinquency. *Psychological Science*, *16*(4), 328–335. <https://doi.org/10.1111/j.0956-7976.2005.01535.x>

Druică, E., Musso, F., & Ianole-Călin, R. (2020). Optimism Bias during the Covid-19 Pandemic: Empirical Evidence from Romania and Italy. *Games*, *11*(3), 39. <https://doi.org/10.3390/g11030039>

Dryhurst, S., Schneider, C. R., Kerr, J., Freeman, A. L. J., Recchia, G., Bles, A. M. van der, Spiegelhalter, D., & Linden, S. van der. (2020). Risk perceptions of COVID-19 around the world. *Journal of Risk Research*, *23*(7–8), 994–1006. <https://doi.org/10.1080/13669877.2020.1758193>

Dunlap, R. E., & Jacques, P. J. (2013). Climate Change Denial Books and Conservative Think Tanks: Exploring the Connection. *American Behavioral Scientist*, *57*(6), 699–731. <https://doi.org/10.1177/0002764213477096>

Erceg, N., Ružojčić, M., & Galić, Z. (2020). Misbehaving in the Corona crisis: The role of anxiety and unfounded beliefs. *Current Psychology*. <https://doi.org/10.1007/s12144-020-01040-4>

Frederick, S. (2005). Cognitive reflection and decision making. *Journal of Economic Perspectives*, *19*(4), 25–42. <https://doi.org/10.1257/089533005775196732>

Furnham, A. (2013). Commercial conspiracy theories: A pilot study. *Frontiers in Psychology*, *4*. <https://doi.org/10.3389/fpsyg.2013.00379>

Gaissmaier, W. (2019). A Cognitive-Ecological Perspective on Risk Perception and Medical Decision Making. *Medical Decision Making: An International Journal of the Society for Medical Decision Making*, *39*(7), 723–726. <https://doi.org/10.1177/0272989X19876267>

Gerrard, M., Gibbons, F., Reis-Bergan, M., & Russell, D. (2001). Self-Esteem, Self-Serving Cognitions, and Health Risk Behavior. *Journal of Personality*, *68*, 1177–1201. <https://doi.org/10.1111/1467-6494.00131>

Gino, F., Norton, M. I., & Weber, R. A. (2016). Motivated Bayesians: Feeling Moral While Acting Egoistically. *Journal of Economic Perspectives*, *30*(3), 189–212. <https://doi.org/10.1257/jep.30.3.189>

Gkinopoulos, T., & Hegarty, P. (2018). Commemoration in crisis: A discursive analysis of who ‘we’ and ‘they’ have been or become in ceremonial political speeches before and during the Greek financial downturn. *British Journal of Social Psychology*, *57*(3), 591–609. <https://doi.org/10.1111/bjso.12244>

Golec de Zavala, A., Cichocka, A., & Iskra-Golec, I. (2013). Collective Narcissism Moderates the Effect of In-Group Image Threat on Intergroup Hostility. *Journal of Personality and Social Psychology*, *104*. <https://doi.org/10.1037/a0032215>

Golec de Zavala, A., Cichocka, A., Eidelson, R., & Jayawickreme, N. (2009). Collective narcissism and its social consequences. *Journal of Personality and Social Psychology*, *97*(6), 1074–1096. <https://doi.org/10.1037/a0016904>

Gollwitzer, A., Martel, C., Brady, W. J., Pärnamets, P., Freedman, I. G., Knowles, E. D., & Van Bavel, J. J. (2020). Partisan differences in physical distancing are linked to health outcomes during the COVID-19 pandemic. *Nature Human Behaviour*, *4*(11), 1186–1197. <https://doi.org/10.1038/s41562-020-00977-7>

Grebe, E., & Nattrass, N. (2012). AIDS conspiracy beliefs and unsafe sex in Cape Town. *AIDS and Behavior*, *16*(3), 761–773. <https://doi.org/10.1007/s10461-011-9958-2>

Harvatt, J., Petts, J., & Chilvers, J. (2011). Understanding householder responses to natural hazards: Flooding and sea‐level rise comparisons. *Journal of Risk Research*, *14*(1), 63–83. <https://doi.org/10.1080/13669877.2010.503935>

Hawkley, L. C., & Cacioppo, J. T. (2010). Loneliness matters: A theoretical and empirical review of consequences and mechanisms. *Annals of Behavioral Medicine: A Publication of the Society of Behavioral Medicine*, *40*(2), 218–227. <https://doi.org/10.1007/s12160-010-9210-8>

Hystad, P., & Carpiano, R. M. (2012). Sense of community-belonging and health-behaviour change in Canada. *Journal of Epidemiology and Community Health*, *66*(3), 277–283. <https://doi.org/10.1136/jech.2009.103556>

Jetten, J., Haslam, C., & Haslam, S. A. (Eds.). (2012). *The social cure: Identity, health and well-being* (pp. xvii, 390). Psychology Press.

Jolley, D., & Douglas, K. M. (2014). The effects of anti-vaccine conspiracy theories on vaccination intentions. *PloS One*, *9*(2), e89177. <https://doi.org/10.1371/journal.pone.0089177>

Jones, D. N., & Paulhus, D. L. (2011). The role of impulsivity in the Dark Triad of personality. *Personality and Individual Differences*, *51*(5), 679–682. <https://doi.org/10.1016/j.paid.2011.04.011>

Jordan, R. E., Adab, P., & Cheng, K. K. (2020). Covid-19: Risk factors for severe disease and death. *BMJ*, *368*, m1198. <https://doi.org/10.1136/bmj.m1198>

Jovančević, A., & Milićević, N. (2020). Optimism-pessimism, conspiracy theories and general trust as factors contributing to COVID-19 related behavior – A cross-cultural study. *Personality and Individual Differences*, *167*, 110216. <https://doi.org/10.1016/j.paid.2020.110216>

Klofstad, C. A., Uscinski, J. E., Connolly, J. M., & West, J. P. (2019). What drives people to believe in Zika conspiracy theories? *Palgrave Communications*, *5*(1), 1–8. <https://doi.org/10.1057/s41599-019-0243-8>

Kurzban, R., Duckworth, A., Kable, J. W., & Myers, J. (2013). An opportunity cost model of subjective effort and task performance. *The Behavioral and Brain Sciences*, *36*(6), 661–679. <https://doi.org/10.1017/S0140525X12003196>

Liao, Q., Wu, P., Wing Tak Lam, W., Cowling, B. J., & Fielding, R. (2019). Trajectories of public psycho-behavioural responses relating to influenza A(H7N9) over the winter of 2014-15 in Hong Kong. *Psychology & Health*, *34*(2), 162–180. <https://doi.org/10.1080/08870446.2018.1515436>

Loewenstein, G. F., Weber, E. U., Hsee, C. K., & Welch, N. (2001). Risk as feelings. *Psychological Bulletin*, *127*(2), 267–286. <https://doi.org/10.1037/0033-2909.127.2.267>

Luo, Y., & Zhao, J. (2019). Motivated Attention in Climate Change Perception and Action. *Frontiers in Psychology*, *10*, 1541. <https://doi.org/10.3389/fpsyg.2019.01541>

Marchlewska, M., Cichocka, A., Jaworska, M., Zavala, A. G. de, & Bilewicz, M. (2020). Superficial ingroup love? Collective narcissism predicts ingroup image defense, outgroup prejudice, and lower ingroup loyalty. *British Journal of Social Psychology*, *59*(4), 857–875. <https://doi.org/10.1111/bjso.12367>

McCright, A. M., & Dunlap, R. E. (2011). The Politicization of Climate Change and Polarization in the American Public’s Views of Global Warming, 2001–2010. *The Sociological Quarterly*, *52*(2), 155–194. <https://doi.org/10.1111/j.1533-8525.2011.01198.x>

McPhetres, J., Bago, B., & Pennycook, G. (2019). *Science beliefs, political ideology, and cognitive sophistication*. OSF Preprints. <https://doi.org/10.31219/osf.io/ad9v7>

Miller, J. D., & Maples, J. (2011). Trait personality models of narcissistic personality disorder, grandiose narcissism, and vulnerable narcissism. In *The handbook of narcissism and narcissistic personality disorder: Theoretical approaches, empirical findings, and treatments* (pp. 71–88). John Wiley & Sons, Inc.

Mulukom, V. van, Pummerer, L., Alper, S., Bai, (Max) Hui, Cavojova, V., Farias, J. E. M., Kay, C. S., Lazarevic, L., Lobato, E. J. C., Marinthe, G., Banai, I. P., Šrol, J., & Zezelj, I. (2020). *COVID-19 conspiracy beliefs are predicted by epistemic and socio-existential motives: A dual pathway model and systematic review*. PsyArXiv. <https://doi.org/10.31234/osf.io/u8yah>

Norris, F. H., Smith, T., & Kaniasty, K. (1999). Revisiting the Experience–Behavior Hypothesis: The Effects of Hurricane Hugo on Hazard Preparedness and Other Self-Protective Acts. *Basic and Applied Social Psychology*, *21*(1), 37–47. <https://doi.org/10.1207/s15324834basp2101_4>

Nowak, B., Brzóska, P., Piotrowski, J., Sedikides, C., Żemojtel-Piotrowska, M., & Jonason, P. K. (2020). Adaptive and maladaptive behavior during the COVID-19 pandemic: The roles of Dark Triad traits, collective narcissism, and health beliefs. *Personality and Individual Differences*, *167*, 110232. <https://doi.org/10.1016/j.paid.2020.110232>

Painter, M., & Qiu, T. (2021). Political beliefs affect compliance with government mandates. *Journal of Economic Behavior & Organization*, *185*, 688–701. <https://doi.org/10.1016/j.jebo.2021.03.019>

Paradise, A. W., & Kernis, M. H. (2002). Self-esteem and Psychological Well-being: Implications of Fragile Self-esteem. *Journal of Social and Clinical Psychology*, *21*(4), 345–361. <https://doi.org/10.1521/jscp.21.4.345.22598>

Pennycook, G., McPhetres, J., Bago, B., & Rand, D. (2020a). *Beliefs about COVID-19 in Canada, the U.K., and the U.S.A.: A novel test of political polarization and motivated reasoning*. PsyArXiv. <https://doi.org/10.31234/osf.io/zhjkp>

Pennycook, G., McPhetres, J., Zhang, Y., Lu, J. G., & Rand, D. G. (2020b). Fighting COVID-19 Misinformation on Social Media: Experimental Evidence for a Scalable Accuracy-Nudge Intervention. *Psychological Science*, *31*(7), 770–780. <https://doi.org/10.1177/0956797620939054>

Pickup, M., Stecula, D., & Linden, C. van der. (2020). Novel Coronavirus, Old Partisanship: COVID-19 Attitudes and Behaviours in the United States and Canada. *Canadian Journal of Political Science/Revue Canadienne de Science Politique*, *53*(2), 357–364. <https://doi.org/10.1017/S0008423920000463>

Pietersma, S., & Dijkstra, A. (2012). Cognitive self-affirmation inclination: An individual difference in dealing with self-threats. *The British Journal of Social Psychology*, *51*(1), 33–51. <https://doi.org/10.1348/014466610X533768>

Postmes, T., Haslam, S. A., & Jans, L. (2013). A single-item measure of social identification: Reliability, validity, and utility. *The British Journal of Social Psychology*, *52*(4), 597–617. <https://doi.org/10.1111/bjso.12006>

Rothgerber, H., Wilson, T., Whaley, D., Rosenfeld, D. L., Humphrey, M., Moore, A., & Bihl, A. (2020). *Politicizing the COVID-19 Pandemic: Ideological Differences in Adherence to Social Distancing*. PsyArXiv. <https://doi.org/10.31234/osf.io/k23cv>

Rothmund, T., Farkhari, F., Azevedo, F., & Ziemer, C.-T. (2020). *Scientific Trust, Risk Assessment, and Conspiracy Beliefs about COVID-19—Four Patterns of Consensus and Disagreement between Scientific Experts and the German Public*. PsyArXiv. <https://doi.org/10.31234/osf.io/4nzuy>

Savadori, L., & Lauriola, M. (2020). Risk Perception and Protective Behaviors During the Rise of the COVID-19 Outbreak in Italy. *Frontiers in Psychology*, *11*, 577331. <https://doi.org/10.3389/fpsyg.2020.577331>

Scheier, M. F., Weintraub, J. K., & Carver, C. S. (1986). Coping with stress: Divergent strategies of optimists and pessimists. *Journal of Personality and Social Psychology*, *51*(6), 1257–1264. [https://doi.org/10.1037//0022-3514.51.6.1257](https://doi.org/10.1037/0022-3514.51.6.1257)

Schutte, N. S., Malouff, J. M., Simunek, M., McKenley, J., & Hollander, S. (2002). Characteristic emotional intelligence and emotional well-being. *Cognition and Emotion*, *16*(6), 769–785. <https://doi.org/10.1080/02699930143000482>

Scolobig, A., De Marchi, B., & Borga, M. (2012). The missing link between flood risk awareness and preparedness: Findings from case studies in an Alpine Region. *Natural Hazards*, *63*(2), 499–520. <https://doi.org/10.1007/s11069-012-0161-1>

Shenhav, A. (2017). The Perils of Losing Control: Why Self-Control Is Not Just Another Value-Based Decision. *Psychological Inquiry*, *28*(2–3), 148–152. <https://doi.org/10.1080/1047840X.2017.1337407>

Shenhav, A., Botvinick, M. M., & Cohen, J. D. (2013). The expected value of control: An integrative theory of anterior cingulate cortex function. *Neuron*, *79*(2), 217–240. <https://doi.org/10.1016/j.neuron.2013.07.007>

Slovic, P., Finucane, M. L., Peters, E., & MacGregor, D. G. (2004). Risk as Analysis and Risk as Feelings: Some Thoughts about Affect, Reason, Risk, and Rationality. *Risk Analysis*, *24*(2), 311–322. <https://doi.org/10.1111/j.0272-4332.2004.00433.x>

Stanley, M. L., Barr, N., Peters, K., & Seli, P. (2020). Analytic-thinking predicts hoax beliefs and helping behaviors in response to the COVID-19 pandemic. *Thinking & Reasoning*, *0*(0), 1–14. <https://doi.org/10.1080/13546783.2020.1813806>

Stanovich, K. E., West, R. F., & Toplak, M. E. (2016). *The Rationality Quotient*. The MIT Press. <https://mitpress.mit.edu/books/rationality-quotient>

Stavrakakis, Y., & Katsambekis, G. (2020). *Populism and the pandemic: A collaborative report* [Report]. Loughborough University. <https://repository.lboro.ac.uk/articles/report/Populism_and_the_pandemic_A_collaborative_report/12546284/1>

Sternisko, A., Cichocka, A., Cislak, A., & Bavel, J. J. V. (2020). *Collective narcissism predicts the belief and dissemination of conspiracy theories during the COVID-19 pandemic*. PsyArXiv. <https://doi.org/10.31234/osf.io/4c6av>

Stoica, C. A., & Umbreș, R. (2021). Suspicious minds in times of crisis: Determinants of Romanians’ beliefs in COVID-19 conspiracy theories. *European Societies*, *23*(sup1), S246–S261. <https://doi.org/10.1080/14616696.2020.1823450>

Tajfel, H. (Ed.). (1978). *Differentiation between social groups: Studies in the social psychology of intergroup relations* (pp. xv, 474). Academic Press.

Teovanović, P., Lukić, P., Zupan, Z., Lazić, A., Ninković, M., & Žeželj, I. (2021). Irrational beliefs differentially predict adherence to guidelines and pseudoscientific practices during the COVID-19 pandemic. *Applied Cognitive Psychology*, *35*(2), 486–496. <https://doi.org/10.1002/acp.3770>

Thoma, V., White, E., Panigrahi, A., Strowger, V., & Anderson, I. (2015). Good thinking or gut feeling? Cognitive reflection and intuition in traders, bankers and financial non-experts. *PloS One*, *10*(4), e0123202. <https://doi.org/10.1371/journal.pone.0123202>

Van Bavel, J. J., Baicker, K., Boggio, P. S., Capraro, V., Cichocka, A., Cikara, M., Crockett, M. J., Crum, A. J., Douglas, K. M., Druckman, J. N., Drury, J., Dube, O., Ellemers, N., Finkel, E. J., Fowler, J. H., Gelfand, M., Han, S., Haslam, S. A., Jetten, J., … Willer, R. (2020). Using social and behavioural science to support COVID-19 pandemic response. *Nature Human Behaviour*, *4*(5), 460–471. <https://doi.org/10.1038/s41562-020-0884-z>

van Holm, E. J., Monaghan, J., Shahar, D. C., Messina, J. P., & Surprenant, C. (2020). *The Impact of Political Ideology on Concern and Behavior During COVID-19* (SSRN Scholarly Paper ID 3573224). Social Science Research Network. <https://doi.org/10.2139/ssrn.3573224>

van Zomeren, M., Postmes, T., & Spears, R. (2008). Toward an integrative social identity model of collective action: A quantitative research synthesis of three socio-psychological perspectives. *Psychological Bulletin*, *134*(4), 504–535. <https://doi.org/10.1037/0033-2909.134.4.504>

Vazire, S., & Funder, D. (2006). Impulsivity and the Self-Defeating Behavior of Narcissists. *Personality and Social Psychology Review : An Official Journal of the Society for Personality and Social Psychology, Inc*, *10*, 154–165. <https://doi.org/10.1207/s15327957pspr1002_4>

Wachinger, G., Renn, O., Begg, C., & Kuhlicke, C. (2013). The risk perception paradox—Implications for governance and communication of natural hazards. *Risk Analysis: An Official Publication of the Society for Risk Analysis*, *33*(6), 1049–1065. <https://doi.org/10.1111/j.1539-6924.2012.01942.x>

Waytz, A., Iyer, R., Young, L., Haidt, J., & Graham, J. (2019). Ideological differences in the expanse of the moral circle. *Nature Communications*, *10*(1), 4389. <https://doi.org/10.1038/s41467-019-12227-0>

Weber, E. U. (2006). Experience-Based and Description-Based Perceptions of Long-Term Risk: Why Global Warming does not Scare us (Yet). *Climatic Change*, *77*(1), 103–120. <https://doi.org/10.1007/s10584-006-9060-3>

Weinstein, N. D. (1987). Unrealistic optimism about susceptibility to health problems: Conclusions from a community-wide sample. *Journal of Behavioral Medicine*, *10*(5), 481–500. <https://doi.org/10.1007/BF00846146>

Weinstein, N. D. (1989). Effects of personal experience on self-protective behavior. *Psychological Bulletin*, *105*(1), 31–50. <https://doi.org/10.1037/0033-2909.105.1.31>

Whitmarsh, L. (2011). Scepticism and uncertainty about climate change: Dimensions, determinants and change over time. *Global Environmental Change*, *21*(2), 690–700. <https://doi.org/10.1016/j.gloenvcha.2011.01.016>

Williams, W. C., Morelli, S. A., Ong, D. C., & Zaki, J. (2018). Interpersonal emotion regulation: Implications for affiliation, perceived support, relationships, and well-being. *Journal of Personality and Social Psychology*, *115*(2), 224–254. <https://doi.org/10.1037/pspi0000132>

Wise, T., Zbozinek, T. D., Michelini, G., Hagan, C. C., & Mobbs, D. (n.d.). Changes in risk perception and self-reported protective behaviour during the first week of the COVID-19 pandemic in the United States. *Royal Society Open Science*, *7*(9), 200742. <https://doi.org/10.1098/rsos.200742>

Wolff, W., & Martarelli, C. S. (2020). Bored Into Depletion? Toward a Tentative Integration of Perceived Self-Control Exertion and Boredom as Guiding Signals for Goal-Directed Behavior. *Perspectives on Psychological Science*, *15*(5), 1272–1283. <https://doi.org/10.1177/1745691620921394>

Wolff, W., Martarelli, C. S., Schüler, J., & Bieleke, M. (2020). High Boredom Proneness and Low Trait Self-Control Impair Adherence to Social Distancing Guidelines during the COVID-19 Pandemic. *International Journal of Environmental Research and Public Health*, *17*(15), 5420. <https://doi.org/10.3390/ijerph17155420>

Xie, X.-F., Stone, E., Zheng, R., & Zhang, R.-G. (2011). The ‘Typhoon Eye Effect’: Determinants of distress during the SARS epidemic. *Journal of Risk Research*, *14*(9), 1091–1107. <https://doi.org/10.1080/13669877.2011.571790>

Xu, D., Peng, L., Liu, S., & Wang, X. (2018). Influences of Risk Perception and Sense of Place on Landslide Disaster Preparedness in Southwestern China. *International Journal of Disaster Risk Science*, *9*(2), 167–180. <https://doi.org/10.1007/s13753-018-0170-0>

Yıldırım, M., Geçer, E., & Akgül, Ö. (2021). The impacts of vulnerability, perceived risk, and fear on preventive behaviours against COVID-19. *Psychology, Health & Medicine*, *26*(1), 35–43. <https://doi.org/10.1080/13548506.2020.1776891>

Zajenkowski, M., Jonason, P. K., Leniarska, M., & Kozakiewicz, Z. (2020). Who complies with the restrictions to reduce the spread of COVID-19?: Personality and perceptions of the COVID-19 situation. *Personality and Individual Differences*, *166*, 110199. <https://doi.org/10.1016/j.paid.2020.110199>

Zaki, J., Ochsner, K. N., & Ochsner, K. (2012). The neuroscience of empathy: Progress, pitfalls and promise. *Nature Neuroscience*, *15*(5), 675–680. <https://doi.org/10.1038/nn.3085>

1. <https://en.wikipedia.org/wiki/Misinformation_related_to_the_COVID-19_pandemic#Conspiracy_theories> [↑](#footnote-ref-1)
